# Polydosetins & pullularins—bioactive tetramic acids & cyclodepsipeptides from the endophytic and nematophagous fungus *Polydomus karssenii*

**DOI:** 10.1007/s13659-025-00579-8

**Published:** 2026-02-03

**Authors:** Natalia A. Llanos-López, Jan-Peer Wennrich, Janette Miled, Samad Ashrafi, Wolfgang Maier, Frank Surup, Marc Stadler

**Affiliations:** 1 Department of Microbial Drugs, Helmholtz Centre for Infection Research (HZI) and German Centre for Infection Research (DZIF), DZIF Partner Site Hannover-Brunswick, Germany, Inhoffenstrasse 7, 38124 Brunswick, Germany; 2 Institute of Microbiology, Technische Universität Braunschweig, Spielmannstraße 7, 38106 Brunswick, Germany; 3 Institute for Epidemiology and Pathogen Diagnostics, Julius Kühn Institute (JKI) - Federal Research Centre for Cultivated Plants, Messeweg 11–12, 38104 Brunswick, Germany; 4 Department of Zoology and Entomology, University of the Free State, Bloemfontein, 9300 South Africa; 5 Laboratory of Fungal Genetics and Metabolism, Institute of Microbiology of the Czech Academy of Sciences, Prague, 14220 Czechia

**Keywords:** *Polydomus*, Tetramic acids, Cyclodepsipeptides, Antimicrobial, Nematicidal

## Abstract

In course of investigating the endophytic and nematode-associated fungus *Polydomus karssenii* for the production of secondary metabolites, seven previously undescribed natural products were isolated from liquid and solid-state fermentations. 1D and 2D NMR spectroscopy, together with HR-ESI–MS data, enabled the elucidation of the planar structures of 3-decalinoyltetramic acids polydosetins A–E (**1**–**5**) and cyclodepsipeptides pullularins G and H (**6** and **7**). The relative configurations of the decalin moiety of **1**–**5** were determined based on ROESY correlations and ^1^H–^1^H coupling constants. The configuration of the side chains was established through a detailed *J*-resolved analysis (Murata’s method) in combination with chemical shift comparison to model compounds. Absolute stereochemistry of **1**–**5** was assigned based on ECD data, and confirmed by Mosher’s method utilizing **3**. Finally, the absolute configuration of amino acid residues in **6** and **7** was determined through advanced Marfey’s method. Bioassays revealed that compounds **1**, **3**, **5**, and **7** were active against Gram-positive bacteria, **3** and **5** exhibited antifungal activity, and **1** and **2** showed nematicidal effects. These results underscore the untapped chemical potential of *P. karssenii* and highlight the importance of exploring nematode-associated fungi as sources of new natural products with potential antimicrobial and nematicidal properties.

**Supplementary Information:**

The online version contains supplementary material available at 10.1007/s13659-025-00579-8.

## Introduction

Natural products constitute a rich reservoir of structurally diverse molecules with therapeutic potential, and fungi serve as an invaluable source of these bioactive compounds [1]. Several fungi have been extensively studied for the production of secondary metabolites, including common soil molds such as *Aspergillus* and *Penicillium* in filamentous fungi, while the majority of the fungal kingdom remains largely unexplored [2, 3]. Among these are nematode-associated fungi, of which only a few species have been studied to date, including *Laburnicola nematophila* and *Polyphilus sieberi* [4, 5]. Both species produce bioactive secondary metabolites. Dactylfungin derivatives from *L. nematophila* exhibit activity against azole-resistant *Aspergillus fumigatus*, while talaroderxine D from *P. sieberi* inhibits the formation of *Staphylococcus aureus* biofilms [4, 5]. Thus, these two species exemplarily demonstrate the broad biosynthetic potential of nematode-associated fungi.

The nematode-associated genus *Polydomus* was recently introduced by Ashafi et al. (2023) as a monophyletic lineage within the family Phaeosphaeriaceae (Pleosporales, Dothideomycetes) [6]. *Polydomus karssenii*, isolated from eggs of the cereal cyst nematode *Heterodera filipjevi* and from the plant *Microthlaspi perfoliatum*, is a fungus with a bifunctional lifestyle, functioning both as a plant-inhabiting dark-septate endophyte and as a nematode parasite [6]. Prior to its formal description, the metabolites ophiotine, xanthomide Z, arthrichitin, and arthrichitins B and C were isolated from a single strain of *P. karssenii*, of which ophiotine showed nematicidal activity [7]. A subsequent metabolite profile study across multiple strains of this species revealed the production of a nearly identical set of secondary metabolites among the studied strains [6]. However, the chemical diversity of *P. karssenii* remains only partially explored, and the majority of its compounds have yet to be purified and structurally characterized.

Here, we report the isolation and characterization of seven previously undescribed tetramic acids and cyclodepsipeptides obtained from two strains of *P. karssenii*. One strain (JKI 73120) was isolated as an endophytic fungus from the roots of *M. perfoliatum*, whereas the other strain (DSM 111209) was obtained as the infecting fungal agent of eggs of the plant-parasitic nematode *H. filipjevi*. In this study, we also evaluated the antimicrobial, cytotoxic, and nematicidal activities of the newly discovered secondary metabolites.

## Materials and methods

### Chromatography and spectral methods

Crude extracts from *P. karssenii* strains JKI 73120 and DSM 111209 were diluted to a concentration of 4.5 mg/mL in a solution of methanol (MeOH) and acetone (1:1), while fractions and pure compounds were diluted to a concentration of 1 mg/mL in the same solution. The samples were analyzed using an analytical HPLC system (UltiMate^®^ 3000 series uHPLC, Thermo Fisher Scientific^®^, MA, USA) coupled to an ion trap mass spectrometer (amaZon speed™, Bruker, Billerica, MA, USA), operating in both positive and negative ionization modes. Pure compounds were further analyzed by high-resolution mass spectrometry (HR-ESI–MS) at a concentration of 0.1 mg/mL in the same solvent system, using a trapped ion mobility quadrupole time-of-flight mass spectrometer (timsTOF Pro 2, Bruker Daltonics, Bremen, Germany) coupled to an Agilent 1290 series HPLC–UV system (Agilent Technologies, Santa Clara, CA, USA). Both systems employed a C18 Acquity^®^ UPLC BEH column (2.1 × 50 mm, 1.7 μm; Waters, Milford, MA, USA) (temperature of the column: 40 °C) as the stationary phase. The mobile phase consisted of Milli-Q water + 0.1% formic acid (FA) as solvent A, and acetonitrile (MeCN) + 0.1% FA as solvent B, with an injection volume of 2 μL. The gradient elution started at 5% B for 0.5 min, then increased to 100% B over 19.5 min, and was maintained at 100% B for 5 min at a flow rate of 0.6 mL/min. UV–Vis spectral data were detected using a Diode-Array Detector (DAD) at 210 nm and 190–600 nm.

UV/Vis, electronic circular dichroism (ECD), and optical rotation (OR) spectra of the pure compounds were recorded in methanol Uvasol^®^ using a Shimadzu UV-2450 UV–Vis spectrophotometer (Shimadzu, Kyoto, Japan), a Jasco J-815 spectropolarimeter (JASCO, Pfungstadt, Germany), and an Anton Paar MCP-150 polarimeter (Anton Paar OptoTec GmbH, Seelze, Germany), respectively.

Nuclear magnetic resonance (NMR) spectra were recorded using a Bruker Avance III 500 MHz spectrometer (Bruker Daltonics^®^, Bremen, Germany; ^1^H-NMR: 500 MHz, ^13^C-NMR: 125 MHz), a Bruker Avance III 600 MHz spectrometer (^1^H-NMR: 600 MHz, ^13^C-NMR: 150 MHz), and a Bruker Avance III 700 MHz spectrometer (^1^H-NMR: 700 MHz, ^13^C-NMR: 175 MHz). Chemical shifts were referenced to the solvent peaks DMSO–*d*_6_ (*δ*_H_ 2.50, *δ*_C_ 39.51), acetone–*d*_6_ (*δ*_H_ 2.05, *δ*_C_ 29.32), CH_3_OH–*d*_4_ (*δ*_H_ 3.31, *δ*_C_ 49.15), pyridine–*d*_5_ (*δ*_H_ 7.22, *δ*_C_ 123.87), or CHCl_3_–*d* (*δ*_H_ 7.27, *δ*_C_ 77.00).

### Origin of fungal strains

The morphological characteristics and multilocus phylogeny of *P. karssenii* strains JKI 73120 and DSM 111209 were previously described in detail by Ashrafi et al. (2023). Strain JKI 73120 was isolated as an endophyte from the roots of *M. perfoliatum*, whereas strain DSM 111209 was obtained from infected eggs of the plant-parasitic nematode *H. filipjevi*. The strains were maintained on yeast malt agar (YM agar, malt extract 10 g/L, yeast extract 4 g/L, D-glucose 4 g/L, agar 20 g/L, pH 6.3 before autoclaving) at 23 °C in the dark. Both strains are preserved in the fungal strain collection of the Julius Kühn Institute (JKI) in Braunschweig, Germany, while DSM 111209 is also deposited at the Leibniz Institute DSMZ in Braunschweig, Germany.

### Cultivation of *P. karssenii* strains and extraction

Precultures of *P. karssenii* strains JKI 73120 and DSM 111209 were prepared in individual 250 mL Erlenmeyer flasks, each containing 100 mL of Q6 ½ medium (D-glucose 2.5 g/L, glycerol 10 g/L, cottonseed flour 5 g/L; pH 7.2 before autoclaving). Each flask was inoculated with three 0.25 cm^2^ mycelial plugs grown on YM 6.3 agar. Cultivation was carried out over five days at 23 °C and 140 rpm in the dark. The resulting precultures were homogenized using an Ultra-Turrax (T25 easy clean digital, IKA) equipped with an S25 N-25F dispersing tool at 5000 rpm for 30 s.

For the scale-up fermentation aimed at secondary metabolite production, strain JKI 73120 was subjected to solid-state fermentation in autoclaved brown rice-based (BRFT) and whole oat-based (WOFT) media. Each medium was prepared with 100 mL of a solution containing KH₂PO₄ (0.5 g/L), sodium tartrate (0.5 g/L), and yeast extract (1 g/L), along with 28 g of cereal substrate. The cultivation was carried out in twelve 500 mL Erlenmeyer flasks per medium, each flask inoculated with 6 mL of the preculture and incubated for 2, 4, and 6 weeks (four flasks for each time point) at room temperature. The cultivation was terminated after 2, 4, and 6 weeks, and each flask was harvested with 3 × 250 mL of acetone, thoroughly mixed, and processed according to the previously described protocol [8]. The resulting extracts were defatted through liquid–liquid partitioning with *n*-heptane and MeOH. Both phases were evaporated until dryness and analyzed using HPLC–DAD/MS.

Strain DSM 111209 was cultivated in liquid Q6 ½ medium, using twelve 1-L flasks filled with 400 mL of medium and inoculated with 2 mL of preculture. The cultures were incubated at 23 °C and 140 rpm in the dark. Fermentation progress was monitored by measuring the free glucose concentration using Medi-Test Glucose test strips (Macherey–Nagel, Düren, Germany). Complete glucose consumption occurred after 8 days, and fermentation ended 2 days after glucose was depleted. The mycelium and supernatant were separated by filtration, and both phases were extracted according to the previously established method [8]. Secondary metabolites from the supernatant were recovered using 2% (*m/v*) of AmberLite™ XAD16N adsorbent resin (Sigma-Aldrich, Deisenhofen, Germany) as previously described [8].

### Isolation of compounds 1–7

#### Isolation of compounds from *P. karssenii* strain JKI 73120

The methanol extracts from strain JKI 73120, cultivated in BRFT and WOFT for 2–6 weeks (see Sect. 2.3), were combined based on similar HPLC–DAD/MS profiles, yielding 3.2 g of crude material. This extract was fractionated using normal-phase flash chromatography, followed by multiple rounds of preparative reversed-phase (RP) HPLC, which resulted in the isolation of compounds **2**, **3**, and **6**. Similarly, the *n*-heptane extracts (3.6 g) from both media, which also exhibited comparable metabolite profiles, were combined and subjected to normal-phase flash chromatography. Major pooled fractions were subsequently purified by preparative and semipreparative RP-HPLC, yielding compounds **3**, **5**, and **7**. Details of chromatographic conditions, gradient elutions, retention times, and yields are provided in the Supplementary Information (Scheme S1, Tables S3–S11).

#### Isolation of compounds from *P. karssenii* strain DSM 111209

The mycelium extract (877 mg) obtained from the fermentation of strain DSM 111209 in Q6 ½ medium (see Sect. 2.3) was subjected to flash chromatography separation using a Büchi C-850 Flash Prep system (Büchi Labortechnik AG, Flawil, Switzerland) operating in normal-phase mode. Separation was performed on a 12 g silica cartridge (FlashPure ID Silica, Büchi). Three solvents served as the mobile phase: solvent A was *n*-heptane with 0.1% FA, solvent B contained 63% *n*-heptane, 35.5% TBME, and 1.5% MeOH with 0.1% FA, and solvent C was MeOH with 0.1% FA. The flow rate was maintained at 60 mL/min. The gradient was programmed as follows: isocratic elution at 0% AB for 3 min, followed by a linear gradient to 100% AB over 15 min, and then an isocratic hold at 100% AB for 3 min. The mobile phase was switched to a BC system, starting with 50% BC for 2 min, followed by a linear gradient to 100% BC over 1 min, and maintained at 100% BC for 5 min. Fractions were monitored using UV detection at wavelengths of 210, 254, 300, and 350 nm and collected based on their chromatographic profiles. This separation yielded a fraction of 420 mg with a retention time (*t*_R_) of 22.1–23.7 min and was further purified by RP-HPLC on the same equipment, employing a Gemini 10 µm C18 110 Å column (250 × 50 mm, Phenomenex) as the stationary phase. A binary solvent system, flowing at 50 mL/min, consisting of solvent A (Milli-Q water + 0.1% FA) and solvent B (MeCN + 0.1% FA) was used. A two-stage gradient program was implemented: starting with 50% B held isocratically for 5 min, followed by a linear gradient to 100% B over 40 min, which was then maintained for 10 min. From this separation, a fraction of 60 mg with a *t*_R_ = 42.1–45.2 min was collected and further separated to obtain compounds **1** (8.0 mg, *t*_R_ = 17.1 min) and **4** (6 mg, *t*_R_ = 23.1 min) by preparative RP-HPLC (PLC 2050, Gilson). Separation was performed using a Luna 5 μm C18 110 Å column (250 × 21.2 mm; Phenomenex). The mobile phase (solvents A and B) was maintained at a flow rate of 20 mL/min. The separation was eluted with 65% B for 5 min, then ramped linearly to 90% B over 40 min, followed by a rapid increase to 100% B over 2 min, which was maintained for an additional 10 min.

In parallel with the purification of the mycelial extract, the crude extract from the supernatant (248 mg), recovered using XAD16N adsorbent resin (Sect. 2.3), was subjected to fractionation. The Büchi C-850 Flash Prep system, configured in reversed-phase mode, was utilized for the separation with a Gemini 10 µm C18 110 Å column (250 × 50 mm, Phenomenex). Solvents A and B were used, with the flow rate set to 50 mL/min. The following gradient was applied: 50% B for 5 min, a linear increase to 100% B over 40 min, and then held at 100% B for 10 min. Two subfractions were collected: 5 mg (*t*_R_ = 34.0–34.6 min) and 40 mg (*t*_R_ = 35.1–39.8 min), which underwent further separation. Compound **2** (4.8 mg, *t*_R_ = 17.1 min) was purified from the first subfraction using a semipreparative RP-HPLC system (1200 Infinity Series, Agilent Technologies Deutschland GmbH, Böblingen, Germany). The system was equipped with an XBridge BEH 5 μm C18 column (250 × 10 mm; Waters, Eschborn, Germany). The mobile phase consisted of solvents A and B, maintained at a flow rate of 5 mL/min. The chromatographic profile consisted of an initial isocratic step at 50% B for 3 min, followed by a linear gradient to 70% B over 16 min, a 2-min increase to 100% B, and a final isocratic step at 100% B for 3 min. From the second subfraction, compound **1** (6.8 mg, *t*_R_ = 24.8 min) was isolated using a preparative RP-HPLC system (PLC 2050, Gilson), equipped with a Luna 5 μm C18 110 Å column (250 × 21.2 mm; Phenomenex). Solvents A and B were employed with a flow rate of 20 mL/min. The gradient elution started with an isocratic condition at 60% B for 30 min, followed by a linear increase to 100% B over 5 min, and was maintained at 100% B for 10 min. A detailed description of the separation conditions is provided in the Supporting Information appendix (Scheme S2, Tables S12–S17).

Polydosetin A (**1**): brown solid; $[\alpha]^{20}_{{\rm D}}$ − 4.2 (*c* 0.1, MeOH); UV/Vis (MeOH): λmax (log *ε*) = 289 (4.0), 202 (4.2) nm; ECD (MeOH, λ (nm) (Δε), c = 4.3 × 10^ − ^^4^ M): 273 (−10.3), 252 (−6.6), 235 (−7.4), 223 (−5.5), 200 (−51.2); ^1^H NMR (700 MHz, CH_3_OH–*d*_4_): see Table 2; ^1^H NMR data (700 MHz, acetone–*d*_6_; 500 MHz, chloroform–*d*; 700 MHz, pyridine–*d*_5_; 700 MHz, DMSO–*d*_6_): see Table S2; ^13^C NMR (175 MHz, CH_3_OH–*d*_4_): see Table 1; ^13^C NMR data (175 MHz, aceton–*d*_6_; 125 MHz, chloroform–*d*; 175 MHz, pyridine–*d*_5_; 175 MHz, DMSO–*d*_6_): see Table S1; HRESI-MS: *m/z* 462.2460 [M + H]^+^ (calcd. 462.2492 for C_25_H_36_NO_7_), 484.2277 [M + Na]^+^ (calcd. 484.2311 for C_25_H_35_NNaO_7_).

**Table 1:** ^13^C NMR data of polydosetins A–E in CH_3_OH–*d*_4_

pos.	1^a^	2^b^	3^a^	4^b^	5^b^
1	201.6, C	201.5, C	201.4, C	203.3, C	202.8, C
2	49.6, C	51.2, C	52.2, C	52.6, C	52.5, C
3	57.5, CH	50.6, CH	57.5, CH	57.5, CH	57.3, CH
4	133.0, C	133.8, C	133.1, C	132.9, C	132.9, C
5	128.2, CH	127.1, CH	128.2, CH	128.2, CH	128.3, CH
6	33.7, CH	34.3, CH	33.8, CH	33.7, CH	33.7, CH
7	41.2, CH_2_	41.2, CH_2_	41.3, CH_2_	41.2, CH_2_	41.3, CH_2_
8	29.5, CH	29.5, CH	29.5, CH	29.5, CH_2_	29.5, CH
9	34.1, CH_2_	33.9, CH_2_	34.1, CH_2_	34.1, CH_2_	34.1, CH_2_
10	24.5, CH_2_	24.0, CH_2_	24.5, CH_2_	24.4, CH_2_	24.5, CH_2_
11	43.2, CH	42.5, CH	43.2, CH	43.2, CH_2_	43.3, CH
12	15.7, CH_3_	14.4, CH_3_	15.7, CH_3_	15.6, CH_3_	15.7, CH_3_
13	136.0, C	132.4, CH	136.3, C	136.4, C	136.5, C
14	125.9, CH	128.9, CH	125.7, CH	125.6, CH	125.4, CH
15	13.8, CH_3_	18.2, CH_3_	13.8, CH_3_	13.8, CH_3_	13.7, CH_3_
16	19.4, CH_3_	19.4, CH_3_	19.4, CH_3_	19.4, CH_3_	19.4, CH_3_
17	23.0, CH_3_	22.7, CH_3_	23.2, CH_3_	23.0, CH_3_	23.0, CH_3_
18	14.7, CH_3_		14.6, CH_3_	14.6, CH_3_	14.4, CH_3_
2’	n.o	n.o	181.5, C	n.o	n.o
3’	n.o	101.2, C	102.5, C	n.o	n.o
4’	n.o	n.o	193.9, C	195.3, C	194.5, C
5’	63.2, CH	n.o	63.7, CH	n.o	60.9, CH
6’	72.8, CH	72.9, CH	73.0, CH	69.0, CH	28.2, CH_2_
7’	74.5, CH	74.5, CH	74.4, CH	38.6, CH_2_	30.2, CH_2_
8’	176.0, C	176.0, C	174.8, C	177.5, C	176.6, C
8’–CH_3_			52.7, CH_3_		

n.o. not observed, ^a^ 700 MHz, ^b^ 500 MHz

Polydosetin B (**2**): yellow solid; $[\alpha]^{20}_{{\rm D}}$ − 4.0 (*c* 0.1, MeOH); UV/Vis (MeOH): λmax (log *ε*) = 288 (4.0), 204 (4.1) nm; ECD (MeOH, λ (nm) (Δε), c = 4.5 × 10^−4^ M): 279 (− 9.0), 250 (− 3.6), 234 (− 5.0), 223 (− 4.2), 198 (− 43.0); ^1^H NMR (500 MHz, CH_3_OH–*d*_4_): see Table 2; ^13^C NMR (125 MHz, CH_3_OH–*d*_4_): see Table 1; HRESI-MS: *m/z* 448.2309 [M + H]^+^ (calcd. 448.2335 for C_24_H_34_NO_7_), 470.2124 [M + Na]^+^ (calcd. 470.2155 for C_24_H_33_NNaO_7_, 895.4497 [2M + H]^+^ (calcd. 895.4592 for C_48_H_67_N_2_O_14_).

**Table 2:** ^1^H NMR data of polydosetins A–E in CH_3_OH–*d*_4_

Pos	**1**^a^	**2**^b^	**3**^b^	**4**^b^	**5**^b^
3	3.30, br s	3.32, m	3.32, m	3.32, m	3.35, m
5	5.22, s	5.23, s	5.22, s	5.23, s	5.23, s
6	2.07, m	2.08, m	2.06, m	2.08, m	2.07, m
7	1.64, m 1.47, m	1.61, m 1.44, m	1.64, m 1.48, m	1.65, m 1.48, m	1.65, m 1.48, m
8	2.07, m	2.08, m	2.07, m	2.08, m	2.07, m
9	1.69, m 1.54, br d (13.0)	1.73, m 1.55, m	1.69, m	1.69, m 1.55, m	1.68, m 1.54, m
10	1.62, m 1.22, m	1.78, m 1.24, m	1.61, m	1.63, m 1.24, m	1.61, m 1.22, m
11	1.89, br t (10.2)	1.70, m	1.89, br t (10.4)	1.90, m	1.90, m
12	1.49, s	1.44, s	1.48, s	1.49, s	1.49, s
13		5.13, m			
14	5.25, br s	5.27, m	5.25, br s	5.25, m	5.24, m
15	1.46, m	1.55, br d (6.7)	1.45, m	1.46, m	1.47, m
16	1.05, d (7.3)	1.04, d (7.3)	1.04, d (7.3)	1.05, d (7.3)	1.05, d (7.3)
17	1.50, br s	1.58, m	1.50, br s	1.51, m	1.51, m
18	1.46, m		1.46, br s	1.46, m	1.46, m
1’NH	n.o	n.o	4.63, br s	n.o	n.o
5’	n.o	3.90, br s	4.00, br s	3.98, br s	3.90, br t (4.7)
6’	4.17, dd (6.0,2.0)	4.17, dd (6.3,2.0)	4.13, dd (7.5,2.0)	4.28, dd (9.6, 3.4)	2.13, m 1.88, m
7’	4.23, d (6.0)	4.23, d (6.3)	4.22, d (7.5)	2.13, m 1.84, m	2.36, m
8’OMe			3.77, s		

n.o. not observed, ^a^ 175 MHz, ^b^ 125 MHz

Polydosetin C (**3**): yellow amorphous solid; $[\alpha]^{20}_{{\rm D}}$ − 2.3 (*c* 0.1, MeOH); UV/Vis (MeOH): λmax (log *ε*) = 288 (3.8), 251 (3.7) nm, 202 (4.0) nm; ECD (MeOH, λ (nm) (Δε), c = 4.2 × 10 ^−^ ^4^ M): 278 (− 7.7), 253 (− 4.6), 234 (− 5.5), 224 (− 3.4), 199 (− 34.7), 194 (− 26.4); ^1^H NMR (700 MHz, CH_3_OH–*d*_4_): see Table 2; ^13^C NMR (125 MHz, CH_3_OH–*d*_4_): see Table 1; HRESI-MS: *m/z* 476.2618 [M + H]^+^ (calcd. 476.2643 for C_26_H_38_NO_7_), 498.2432 [M + Na]^+^ (calcd. 498.2468 for C_26_H_37_NNaO_7_), 951.5112 [2M + H]^+^ (calcd. 951.5218 for C_52_H_75_N_2_O_14_.

Polydosetin D (**4**): yellow solid; $[\alpha]^{20}_{{\rm D}}$ − 3.6 (*c* 0.1, MeOH); UV/Vis (MeOH): λmax (log *ε*) = 288 (4.0), 254 (3.8) nm, 203 (4.2) nm; ECD (MeOH, λ (nm) (Δε), c = 4.5 × 10 ^−^ ^4^ M): 272 (−8.6), 253 (−0.1), 241 (−5.4), 223 (−4.2), 199 (−47.4); ^1^H NMR (500 MHz, CH_3_OH–*d*_4_): see Table 2; ^13^C NMR (125 MHz, CH_3_OH–*d*_4_): see Table 1; HRESI-MS: *m/z* 446.1941 [M + H]^+^ (calcd. 446.2543 for C_25_H_36_NO_6_), 468.1829 [M + Na]^+^ (calcd. 468.2362 for C_25_H_35_NNaO_6_).

Polydosetin E (**5**): yellow oily; $[\alpha]^{20}_{{\rm D}}$ − 5.3 (*c* 0.1, MeOH); UV/Vis (MeOH): λmax (log *ε*) = 288 (4.3), 254 (4.1) nm, 203 (4.5) nm; ECD (MeOH, λ (nm) (Δε), c = 2.3 × 10^ −^ ^4^ M): 273 (−8.0), 245 (−3.9), 244 (−16.5), 230 (−3.4), 200 (−46.1); ^1^H NMR (500 MHz, CH_3_OH–*d*_4_): see Table 2; ^13^C NMR (125 MHz, CH_3_OH–*d*_4_): see Table 1; HRESI-MS: *m/z* 430.2584 [M + H]^+^ (calcd. 430.2593 for C_25_H_36_NO_5_), 452.2399 [M + Na]^+^ (calcd. 452.2413 for C_25_H_35_NNaO_5_), 859.5050 [2M + H]^+^ (calcd. 859.5109 for C_50_H_71_N_2_O_10_).

Pullularin G (**6**): yellow solid; $[\alpha]^{20}_{{\rm D}}$ − 4.2 (*c* 0.1, MeOH); UV/Vis (MeOH): λmax (log *ε*) = 278 (3.2), 201 (4.6) nm; ECD (MeOH, λ (nm) (Δε), c = 2.4 × 10 ^−^ ^4^ M): 285 (− 2.2), 260 (− 0.9), 228 (− 36.7), 210 (− 14.8), 208 (− 15.6), 194 (+ 53.3); ^1^H NMR (500 MHz, DMSO–*d*_6_) & ^13^C NMR (125 MHz, DMSO–*d*_6_): see Table 3; HRESI-MS: *m/z* 818.4650 [M + H]^+^ (calcd. 818.4704 for C_45_H_64_N_5_O_9_), 840.4467 [M + Na]^+^ (calcd. 840.4523 for C_45_H_63_N_5_NaO_9_).

**Table 3:** NMR data of pullularins G (^1^H 500 MHz, ^13^C 125 MHz) and H (^1^H 700 MHz, ^13^C 175 MHz) in DMSO-*d*_6_

	**6**		**7**	
pos	*δ*_C_, mult	*δ*_H_, mult	*δ*_C_, mult	*δ*_H_, mult
3-Ph-Lac			3-Ph-Lac	
1	165.3, C		165.3, C	
2	71.3, CH	5.61, ps t (7.0)	71.1, CH	5.61, ps t (7.0)
3	36.1, CH_2_	3.06, dd (13.3, 7.5)	36.1, CH_2_	3.06, dd (13.3, 7.4)
		2.86, dd (13.3, 6.7)		2.83, dd (13.3, 6.7)
4	136.6, C		136.3, C	
5,9	129.7, CH	7.21, m	129.7, CH	7.20, m
6,8	128.2, CH	7.24, m	128.1, CH	7.24, m
7	126.4, CH	7.17, m	126.4, CH	7.17, m
Pro			Pro	
1	172.4, C		171.7, C	
2	58.3, CH	4.47, m	58.2, CH	4.32, dd (8.7, 2.5)
3	29.4, CH_2_	2.04, m 1.69, m	29.1, CH_2_	2.05, m 1.67, m
4	23.9, CH_2_	1.79, m	23.8, CH_2_	1.79, m
5	45.8, CH_2_	3.66, m 3.23, m	45.7, CH_2_	3.68, m 3.25, m
Ser			Ala	
1	171.2, C		173.7, C	
2	52.6, CH	4.47, m	44.4, CH	4.69, m
3	61.3, CH_2_	3.56, m	17.1, CH_3_	1.23, d (6.7)
2-NH		9.20, br s		8.87, d (4.1)
*N*-Me-Ile			*N*-Me-Val	
1	168.0, C		168.1, C	
2	64.8, CH	4.56, d (10.7)	65.0, CH	4.54, d (10.8)
3	32.0, CH	2.05, m	25.6, CH	2.50, m
4	24.2, CH_2_	1.28, m 0.91, m	19.5, CH_3_	0.99, d (6.4)
5	12.0, CH_3_	0.92, t (7.3)	18.5, CH_3_	0.81, d (6.9)
6	16,3, CH_3_	0.93, d (6.4)		
2-*N*-Me	28.5, CH_3_	2.48, s	28.4, CH_3_	2.52, m
*O*-preny-Tyr				
1	167.6, C		167.6, C	
2	51.9, CH	4.68, ddd (9.9,8.4,6.7)	51.9, CH	4.67, m
3	36.6, CH_2_	2.86, dd (13.1 10.3) 2.48, m	36.6, CH_2_	2.87, dd (13.1 10.3) 2.50, m
4	129.7, C		129.7, C	
5,9	129.9, CH	7.01, br d (8.8)	129.9, CH	7.02, br d (8.8)
6,8	114.1, CH	6.76, br d (8.8)	114.1, CH	6.76, br d (8.8)
7	156.8, C		156.8, C	
1’	64.1, CH_2_	4.42, m	64.1, CH_2_	4.43, d (6.7)
2’	120.1, CH	5.37, m	120.1, CH	5.58, tspt (6.7, 1.4)
3’	136.8, C		136.8, C	
4’	25.4, CH_3_	1.71, br s	25.4, CH_3_	1.72, br s
5’	18.0, CH_3_	1.67, br s	17.9, CH_3_	1.67, br s
*N*-Me-Leu			*N*-Me-Leu	
1	169.4, C		169.3, C	
2	61.2, CH	3.48, dd (8.7, 5.3)	61.2, CH	3.44, dd (8.6, 5.4)
3	37.2, CH_2_	1.42, m 1.27, m	37.1, CH_2_	1.43, m 1.27, m
4	23.9, CH	1.03, m	23.9, CH	1.02, m
5	21.8, CH_3_	0.73, d (6.6)	21.8, CH_3_	0.72, d (6.6)
6	22.9, CH_3_	0.58, d (6.6)	22.9, CH_3_	0.57, d (6.6)
2-*N-*Me	37.4, CH_3_	2.99, s	37.4, CH_3_	2.98, s

Pullularin H (**7**): yellow solid; $[\alpha]^{20}_{{\rm D}}$ + 2.8 (*c* 0.1, MeOH);); UV/Vis (MeOH): λmax (log *ε*) = 280 (2.9), 202 (4.3) nm; ECD (MeOH, λ (nm) (Δε), c = 2.5 × 10^−^ ^4^ M): 285 (−2.1), 268 (−1.4), 230 (−35.4), 210 (−14.3), 208 (−15.0), 194 (+ 51.3); ^1^H NMR (700 MHz, DMSO–*d*_6_) & ^13^C NMR (175 MHz, DMSO–*d*_6_): see Table 3; HRESI-MS: *m/z* 788.4555 [M + H]^+^ (calcd. 788.4598 for C_44_H_62_N_5_O_8_), 810.4368 [M + Na]^+^ (calcd. 810.4418 for C_44_H_61_N_5_NaO_8_).

### Advanced Marfey’s analysis of compounds 6 and 7

The stereochemistry of the amino acid residues in pullularins G and H (**6** and **7)** was determined using Marfey’s method, adapted from Viehrig et al. [9]. For hydrolysis, **6** and **7** (0.1 mg each) were treated separately with 500 µL of 6 N HCl and heated at 90 °C for 18 h. The hydrolysates were dried under vacuum and dissolved in 200 µL of Milli-Q water. To each solution, 20 µL of 1 M NaHCO₃ and 100 µL of acetone containing 1% derivatization agent Nα-(2,4-dinitro-5-fluorophenyl)-L-alaninamide (FDAA) were added. The mixtures were incubated at 40 °C for 40 min and then evaporated to dryness under vacuum. The residues were suspended in 1 mL of MeOH and analyzed by LC–ESI–MS under the same conditions as previously described. The enantiomeric configuration of the amino acids was determined by comparing their retention times with those of authenticated L- and D-amino acid standards derivatized in parallel. The standards included ^L^-proline, ^D^-proline, *N*-methyl-^L^-leucine, *N*-methyl-^D^-leucine, *N*-methyl-L-isoleucine, ^L^-serine, and ^DL^-serine.

### Derivatization of 3 with *R*- and *S*-MTPA-chloride

Polydosetin C (**3**, 2 mg) was dissolved in 0.7 mL dry pyridine–*d*_5_ and treated with *R*–MTPA chloride to obtain the *S*–MTPA ester of **3**. After stirring at 25 °C for 18 h, the mixture was directly subjected to NMR analysis. ^1^H NMR of 3-*S*-MTPA ester (700 MHz, pyridine–*d*_5_) similar to **3**, but: *δ*_H_ 6.16 (dd, *J* = 6.2, 4.1 Hz, H–6’), 5.24 (d, *J* = 6.2 Hz, H–7’), 5.24 (d, *J* = 6.2 Hz, H–7’), 4.50 (d, *J* = 4.1 Hz, H–5’) ppm.

The *R*–MTPA ester of **3** was obtained analogously by using *S*–MTPA chloride, with ^1^H NMR data (700 MHz, pyridine–*d*_5_) similar to **3**, but: *δ*_H_ 6.24 (dd, *J* = 6.0, 3.5 Hz, H–6’), 5.15 (d, *J* = 6.0 Hz, H–7’), 5.24 (d, *J* = 6.2 Hz, H–7’), 4.71 (d, *J* = 3.5 Hz, H–5’) ppm.

### Antimicrobial assay

Antimicrobial activity was assessed using a serial dilution assay to determine the minimum inhibitory concentration (MIC) following a previously established procedure [10]. Compounds **1**–**7** were tested against the fungi *Schizosaccharomyces pombe* (DSM 70572), *Wickerhamomyces anomalus* (DSM 6766), *Mucor hiemalis* (DSM 2656), *Candida albicans* (DSM 1665), and *Rhodosporidium toruloides* (DSM 10134); the Gram-positive bacteria *Bacillus subtilis* (DSM 10), *Mycobacterium smegmatis* (ATCC 700084), and *Staphylococcus aureus* (DSM 346); and the Gram-negative bacteria *Acinetobacter baumannii* (DSM 30008), *Chromobacterium violaceum* (DSM 30191), *Escherichia coli* (DSM 1116), and *Pseudomonas aeruginosa* (DSM 19882). Ciprofloxacin, oxytetracycline, kanamycin, and gentamycin were used as positive controls for bacterial strains, while nystatin served as the positive control for fungal strains.

### Cytotoxicity assay

The in vitro cytotoxic activity of compounds **1**–**7** was evaluated using the colorimetric tetrazolium dye MTT (3-(4,5-dimethylthiazol-2-yl)-2,5-diphenyltetrazolium bromide) assay as previously described [10]. The mammalian cell lines used in this study were obtained from the DSMZ and included mouse fibroblasts (L929) and endocervical adenocarcinoma cells (KB3.1). Epothilone B was employed as the positive control. The half-maximal inhibitory concentration (IC_50_), corresponding to 50% inhibition of cell growth relative to the control, was calculated.

### Nematode assay against *Caenorhabditis elegans*

#### Nematode culture and synchronization

Synchronization of the nematode population was performed according to a previously described method with minor modifications [11]. *C. elegans* (N2 wild-type strain, obtained from the Caenorhabditis Genetics Center) was cultured monoxenically on nematode growth medium (NGM; per liter: 3 g NaCl, 2.5 g peptone, 20 g agar, 1 mL 1 M CaCl_2_, 25 mL 1 M [pH 6.0] KPO_4_, 1 mL 1 M MgSO_4_, and 1 mL cholesterol [5 mg/mL in ethanol]) seeded with *E. coli* OP50 as the food source. Plates were incubated at 19 °C for ~ 120 h until a high density of eggs was achieved. For synchronization, nematodes and eggs were rinsed from the plates three times with 5 mL 0.9% NaCl into a 15 mL Falcon tube, centrifuged at 600 rpm for 3 min, and the supernatant was discarded. This procedure was repeated until a clear suspension of nematodes was obtained. After the last washing step, the pellet suspended in 2 mL 0.9% NaCl was mixed with 5 mL of bleaching solution (1 mL sodium hypochlorite solution, 0.5 mL 5 M NaOH, and 3.5 mL Milli-Q water). The mixture was incubated for 3 min with gentle swirling and monitored microscopically until adult worms were digested, leaving eggs intact. The reaction was stopped by adding 8 mL 0.9% NaCl, followed by centrifugation at 2500 rpm for 2 min. Eggs were washed three times with 13 mL 0.9% NaCl using centrifugation at 2500 rpm for 2 min each time. If adult residues remained, the bleaching process was repeated with 1 mL bleaching solution for 1 min, followed by washing as described above. After the final wash, the egg suspension was adjusted to 2 mL, diluted with 5 mL 0.9% NaCl, and transferred to a 50 mL Falcon tube. Eggs were incubated in a horizontal position on a shaker at 23 °C and 80 rpm for 18 h to allow hatching. The hatched larvae were centrifuged at 600 rpm for 3 min, the supernatant discarded, and the pellet transferred to fresh NGM plates seeded with *E. coli* OP50. Plates were incubated at 19 °C for 48 h to reach the fourth larval (L4) and adult stages. Nematodes were then rinsed from the plates as described above and adjusted with 0.9% NaCl to a final concentration of 1000 nematodes/mL.

#### Nematode assay procedure

The nematicidal activity of compounds **1**–**7** was evaluated against *C. elegans* following a previously described method [11]. Test compounds were dissolved in MeOH at a stock concentration of 0.15 mg/mL. Aliquots of 200 µL, 100 µL, and 20 µL were transferred into a 48-well microtiter plate to yield final assay concentrations of 100 µg/mL, 50 µg/mL, and 10 µg/mL, respectively. MeOH (100 μL) served as a negative control, while ivermectin (1 µg/mL) was used as the positive control. Compounds were tested in triplicate, and controls in six replicates. Following solvent evaporation under nitrogen, 300 µL of synchronized nematode suspension (1000 nematodes/mL) was added to each well, and the plates were incubated at 23 °C and 150 rpm for 18 h. After incubation, live (motile) and dead (erect and immobile) nematodes were counted from a 30 µL drop, in triplicate, and mortality rates were calculated. The observed mortality rate was corrected using the Schneider-Orelli formula [12]. Compounds were classified as active if they induced ≥ 50% mortality at 100 μg/mL.

## Results and discussion

The solid-state cultures of *P. karssenii* strain JKI 73120 grown in BRFT and WOFT media were fractionated using chromatographic techniques, starting with normal-phase flash chromatography and followed by preparative and semipreparative RP-HPLC of the fractions of interest. This procedure led to the isolation of five previously undescribed natural products: the tetramic acids **2**, **3**, and **5**, and the cyclodepsipeptides **6** and **7**. In parallel, fermentation extracts of strain DSM 111209 cultivated in liquid Q6 ½ medium were subjected to the same fractionation workflow, resulting in the isolation of three new tetramic acids, **1**, **2**, and **4**.

### Structure elucidation of polydosetins A–E (1–5)

**Fig. 1:**
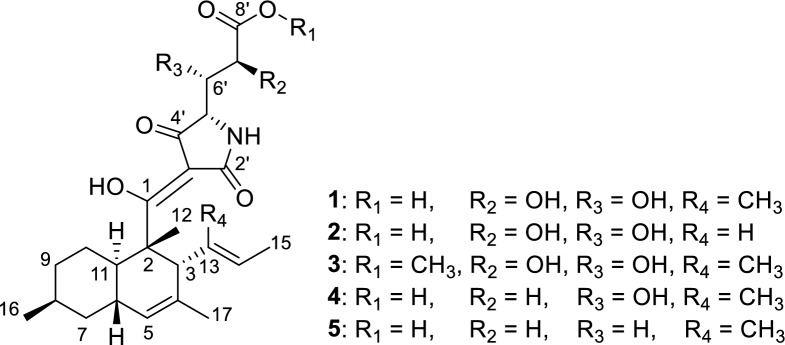
Chemical structures of polydosetins A–E (**1**–**5**)

Compound **1** was assigned the molecular formula C_25_H_35_NO_5_ based on its quasimolecular ion peak cluster at *m/z* 462.2485 in the HRESIMS spectrum, corresponding to 9 degrees of unsaturation. Initial ^1^H and ^13^C NMR spectra recorded in CHCl_3_–*d* showed broad, poorly resolved signals that impeded interpretation. Therefore, additional NMR spectra were also acquired in CH_3_OH–*d*_4_, acetone–*d*₆, pyridine–*d*_5_ and DMSO–*d*₆ (see Tables S1and S2), with acetone–*d*₆ providing the most informative dataset. Still several signals were notably broad or duplicated, likely due to tautomerism or conformational exchange. ^1^H and HSQC NMR spectra revealed the presence of five methyl groups, three methylene groups, two olefinic methines, and six aliphatic methines, of which two were oxymethines and one was an azamethine. The ^13^C NMR spectrum further displayed three unsaturated ketones/oxyolefins (C–1, C–2’, C–4’), one carboxylic carbon (C–8’), three sp^2^-hybridized quaternary carbons (C–10, C–13, C–3’), and one sp^3^-hybridized nonprotonated carbon. COSY and HMBC correlations allowed the assembly of two substructures: a) a bicyclic decalin part, substituted at C–3 with a 1-methylprop-1-enyl group, and b) a 4-amino-2,3,5-trihydroxypentanoyl moiety (see Figure S39 for details). It was not possible to connect these two subunits, since no HMBC correlations were observed between those subunits, nor were HMBC correlations observed to two nonprotonated carbons (C–2’, C–3’). However, a literature search involving these fragments suggested that **1** belongs to the class of decanoyltetramic acids [13]. This compound class is known to undergo multi-sided tautomeric exchange, which leads to signal broadening and often to the absence of expected NMR signals and 2D NMR correlations [14]. Based on all spectroscopic data, the planar structure of **1** was proposed as shown in Fig. 1, for which we suggest the name polydosetin A.

**Fig. 2:**
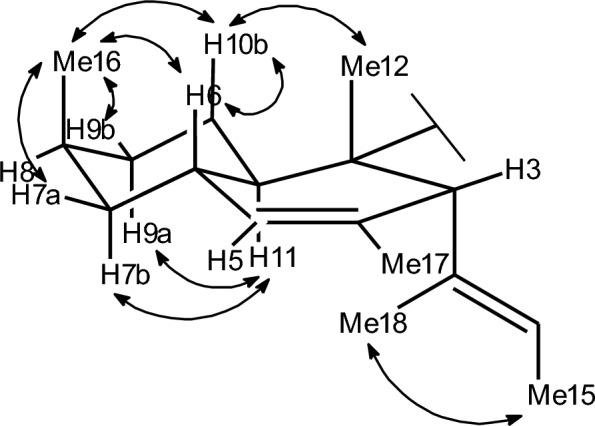
Key ROESY correlations utilized for the structure elucidation of **1**

We utilized a multitude of methods to assign the stereochemistry of all asymmetric centers of **1**. The absolute configurations at C–2 and C–5′ were determined by the comparison of ECD spectroscopic data with those of the known data of structurally related compounds trichosetin and *epi*-trichosetin; minima at both 230 and 280 nm indicate a 2*S*,5’*S* configuration [15]. The ROESY correlations between H_3_–12 and H–3, as well as between H_3_–12 and H–6, specified that the stereocenters C–2, C–3, and C–6 are conserved relative to hymenosetin (Fig. 2). The large coupling constant of ^3^*J*_H6,H11_ = 10.2 Hz, observed in the pseudo triplet of H–11, confirmed the *trans* configuration of these protons. A detailed comparison of the ^13^C NMR chemical shifts with those of hymenosetin indicated a high degree of similarity, thereby confirming that the stereogenic centers possess a common configuration. However, comparative analysis of the ^13^C NMR chemical shifts with those of the closely related hymenosetin [16], CJ-17,572  [17], and colposetin [18] revealed significant shift differences at C–8 and C–16, suggesting an inversed stereochemistry at this position. Since the C–6 and C–8 signals overlapped in acetone–*d*_6_, impeding the investigation, ROESY correlations were analyzed in methanol–*d*_4_. Strong 1,3-diaxial ROESY correlations between H_3_–16, H–6, and H–10b confirmed the assumed 8*S* configuration. The relative configuration of C–6′/C–7 was assigned by a *J*-based configurational analysis. Despite the measurement of HSQC-Hecade and *J*-HMBC NMR experiments in multiple solvents, only the coupling constants ^3^*J*_H6′,H7′_ = 6.0 Hz, ^2^*J*_H7′,C6′_ = 5.0 Hz and ^2^*J*_H6′,C7′_ = 1.9 Hz could be extracted from data obtained in acetone–*d*_6_. Because the ^3^*J*(H6′,H7′) coupling constant is classified as ‘medium’ in the sense of Matsumori et al. (1999) [19], a straightforward analysis based on a single rotamer is not possible. Rather, the observed data indicate an equilibrium of conformations. Nevertheless, the combination of a ‘large’ ^2^*J*(H7′,C6′) and a ‘small’ ^2^*J*(H6′,C7′) provides a clear diagnostic signature for the dihydroxy configuration as *erythro*. As only ^3^*J*_H5′,H6′_ = 2.0 Hz and ^2^*J*_H5′,C6′_ = 3.8 Hz were observed, the *J*-based analysis could not be extended to C–5 and therefore did not allow a direct correlation with the C–5 absolute configuration determined by ECD data.

However, NMR data have been reported for all diastereomers of synthetic 3,4-dihydroxyglutamic acid, which can be regarded as a reliable model compound [20, 21]. Based on this comparison, the observed ‘small’ ^3^*J*(H5′,H6′) and ‘medium’ ^3^*J*(H6′,H7′) coupling constants are characteristic for the 5′*S*,6′*S*,7′*S* configuration.

Polydosetin B (**2**) was isolated as a co-metabolite alongside A and exhibited a quasimolecular ion cluster at *m/z* 448.2335 in the HRESIMS spectrum, consistent with a molecular formula of C_24_H_33_NO_7_, indicating one methyl group less than A. Comparative analysis of the NMR data revealed nearly identical chemical shifts and coupling patterns to those of A, with a notable absence of the methyl group C–18, confirming B as the 13-demethyl derivative of **1**. The loss of the C–18 methyl group resulted in subtle deshielding effects at adjacent positions (particularly at C–12 and C–14, see Table 1), but the overall connectivity and stereochemical configuration remained conserved. ROESY and HMBC data supported an identical planar structure to **1**, minus the C-13 substituent. ROESY correlations between H–13 and H_3_–15 as well as H–3 and H–14 indicate an unchanged *E* configuration of the Δ^13,14^ double bond. Thus, polydosetin B (**2**) was unambiguously identified as the 13-demethyl derivative of **1**.

Polydosetin C (**3**) was identified as a structural analogue of **1**, showing a quasimolecular ion cluster at *m/z* 476.2640 in the HRESIMS spectrum, corresponding to a molecular formula of C_26_H_37_NO_7_, indicating the presence of an additional oxygen atom. Comparison of the ^1^H and ^13^C NMR spectra of C with those of A revealed high overall similarity, with the most prominent difference being the appearance of a methoxy singlet (*δ*_H_ 3.77 ppm, *δ*_C_ 52.7 ppm), consistent with a methoxy group. HMBC correlations from the methoxy protons to C–8′ confirmed that the substitution occurred at this position and unambiguously identified C as the 8′–*O*-methyl derivative of **1**. Polydosetin C (**3**) was used to confirm the stereoconfiguration using a Mosher’s method experiment. The *Δδ*^SR^ chemical shift pattern of the MTPA derivatives of **3**, showing negative values for H-5′ (− 0.21 ppm) and H-6′ (− 0.08 ppm), along with a positive value for H-7′ (+ 0.09 ppm), is characteristic of the *anti-*1,2 Type C arrangement and thus validates our analysis [22].

Compounds D and E (**4** and **5**) were isolated as additional structural analogues of **1**, displaying quasimolecular ion peaks at *m/z* 446.1941 and 430.2584, corresponding to molecular formulae consistent with the loss of one and two oxygen atoms, respectively, relative to **1**. NMR spectral comparison revealed that both compounds shared the same overall core structure as **1**, but differed in the substitution pattern of the side chain.

In **4**, the absence of the C–7′ oxymethine signal was evident in the ^1^H NMR and HSQC spectra, replaced by an aliphatic methylene resonance (*δ*_H_ 2.13 & 1.84 ppm, *δ*_C_ 38.6 ppm). COSY and HMBC correlations confirmed the intact side chain, and no other significant changes were observed. Polydosetin E (**5**) exhibited the additional absence of the C–6′ oxymethine signal, confirming it as the 6′,7′-didehydroxy derivative of **1**. In this compound, both C–6′ and C–7′ appeared as aliphatic methylenes, and HMBC data supported the uninterrupted carbon chain. The rest of the molecule—including the tetramic acid core and its stereochemical features—remained unchanged in both derivatives.

The polydosetins A–E (**1**–**5**) exhibit several unusual structural features: The relative configuration at C–8 to the other stereocenters of the decalin part is particularly noteworthy, as this relatedness has only been observed once in a single related metabolite, the minor compound conipyridoin F [23]. In addition, incorporation of a glutamic acid unit is highly uncommon in tetramic acids; with gabusectin, only one decanoyltetramic acid derivative has been described as being derived from glutamic acid to date [24].

Finally, our results demonstrate that straightforward chemical shift analysis provides a reliable tool for establishing the relative configuration of this class of compounds, offering a practical alternative to more elaborate approaches.

### Structure elucidation of pullularins G and H

The molecular formula of pullularin G (**6**) was established as C_45_H_63_N_5_O_9_ based on the high-resolution electrospray ionization mass spectrum (HRESIMS), which displayed a molecular ion cluster at *m/z* 818.4650. This composition corresponds to 16 degrees of unsaturation.

Initial analysis of the ^1^H, ^13^C, and HSQC NMR spectra indicated that compound **6** possesses a peptidic nature, as evidenced by the presence of five characteristic azamethine signals and six carboxyl carbons resonating between *δ*_C_ 165–172 ppm.

**Fig. 3:**
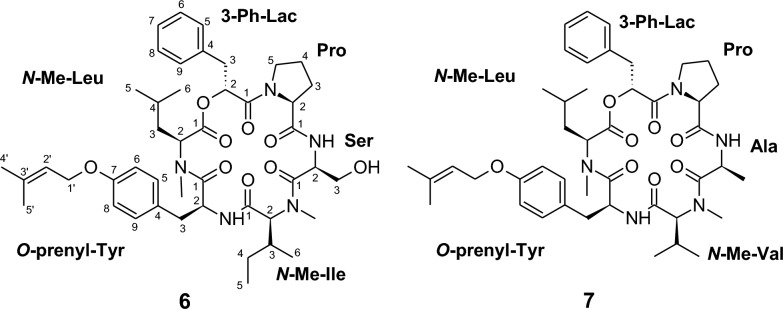
Chemical structures of pullularins G and H (**6** and **7**)

Detailed interpretation of COSY and HMBC correlations allowed the identification of the constituent amino acid residues, namely proline, serine, isoleucine, tyrosine, and leucine. In addition, signals consistent with a 3-phenyl-lactic acid moiety were observed. Structural refinements revealed specific modifications: both the leucine and isoleucine residues were found to be *N*-methylated, while the tyrosine residue carried a prenyl substituent attached to the *para*-oxygen atom of its phenolic group (see Fig. 3).

The sequence of the amino acid residues was established by analyzing long-range HMBC correlations between neighboring units, which enabled the complete assembly of the planar structure. All amino acids were determined to have l-configuration (Figures S58 − S61).

The molecular formula of compound **7** was established as C_44_H_61_N_5_O_8_ based on its HRESIMS data (molecular ion cluster at *m/z* 788.4555). The NMR spectra of **7** were highly similar to those of pullularin G (**6**), confirming a close structural relationship. However, careful analysis revealed two key differences: (i) the terminal oxymethylene group of the serine residue in **6** was replaced by a methyl group in **7**, thus **7** contains an alanine instead of the serine. (ii) The detection of an additional methylene unit in the former valin spin system indicated the substitution by isoleucine. These modifications account for the observed changes in both the molecular composition and the NMR spectroscopic data.

### Bioassays

In our search for novel bioactive natural products, we evaluated the antimicrobial activities of the tetramic acid derivatives polydosetins A–E (**1**–**5**) and the cyclodepsipeptides pullularins G and H (**6** and **7**), isolated from the two studied strains of *P. karssenii*. The compounds were tested against a panel of Gram-positive and Gram-negative bacteria, as well as various fungal strains. The antimicrobial assay results (Table S19) revealed that compounds **1**, **3**, **5**, and **7** (Table 4) displayed activity against the Gram-positive bacterium *B. subtilis*, with polydosetin C (**3**) being the most potent, showing a MIC value of 16.6 µg/mL. Compound **3** also exhibited moderate activity against *S. aureus* (33.3 µg/mL), while **7** showed weak activity against *S. aureus* (66.6 µg/mL) and *M. smegmatis* (66.6 µg/mL). However, none of the tested compounds showed inhibition against Gram-negative bacteria at the tested concentrations (MIC > 66.6 µg/mL). In addition, polydosetin E (**5**) demonstrated antifungal activity against *C. albicans*, *R. toruloides*, and *M. hiemalis*, showing the strongest activity against *M. hiemalis* (16.6 µg/mL). In comparison, compound **3** also exhibited activity against *M. hiemalis*, but with a MIC value of 66.6 µg/mL. Polydosetins A–E (**1**–**5**) belong to the 3‐decalinoyltetramic acids (3-DTAs) class and only a few of them have been reported to affect Gram-negative bacteria; examples include pyrrospirones C, F, and I, which can inhibit the growth of *E. coli* [13, 25]. These findings correlate with the literature, which establishes that 3-DTAs exhibit significant antimicrobial activities, particularly against Gram-positive bacteria, including methicillin-resistant *S. aureus* (MRSA) and *B. subtilis* [13]. This lack of activity could be explained by the fact that the outer membrane barrier of Gram-negative bacteria restricts penetration of 3-DTAs unless their structures incorporate sufficiently lipophilic (decalin-based or other hydrophobic) groups, which may improve outer membrane passage [13]. However, further studies are needed to explain this mechanism. Similar observations have been reported for cyclodepsipeptides, which have demonstrated a broad spectrum of activity against various Gram-positive bacteria, especially *B. subtilis* and *Mycobacterium* spp. [26–29].

**Table 4:** Antimicrobial activities of compounds **1**, **3**, **5**, and **7** in the serial dilution assay. All other compounds (**2**, **4**, and **6**) were devoid of significant effects up to concentrations of 66.6 µg/mL

Test Microorganism	MIC (µg/mL)				Positive control (µg/mL)
	1	3	5	7	
*Staphylococcus aureus*	n.i	33.3	n.i	66.6	0.21^G^
*Mycobacterium smegmatis*	n.i	n.i	n.i	66.6	1.70^ K^
*Bacillus subtilis*	66.6	16.6	66.6	66.6	16.6^O^
*Candida albicans*	n.i	n.i	66.6	n.i	8.3^N^
*Mucor hiemalis*	n.i	66.6	16.6	n.i	8.30^N^
*Rhodosporidium toruloides*	n.i	n.i	66.6	n.d	4.20^N^

*n.i* No inhibition (> 66.6 µg/mL). *n.d* Not determined

^G^: Gentamycin; ^O^: Oxytetracycline; ^N^: Nystatin; ^K^: Kanamycin

On the other hand, methylation of the carboxylic acid group in the studied tetramic acids can significantly affect their biological activity. Compound **3**, which is the 8′–*O*-methyl derivative of **1**, showed a wider range of antimicrobial effects (as shown in Table 4). This indicates that methylation can enhance antimicrobial properties. Similar results have been observed in structure–activity relationship (SAR) studies, where replacing a methyl group in enol ethers with a hydroxyl group caused a notable decrease in bioactivity [30]. However, there are limited direct comparisons between methylated esters and free carboxylic acids across different compounds. Despite this, small chemical modifications like methylation can have a significant impact on the biological activity of these compounds.

All the compounds were tested for their cytotoxic effects (Table S18) on two mammalian cell lines: mouse fibroblasts (L929) and endocervical adenocarcinoma cells (KB3.1). Only polydosetin E (**5**) and pullularin H (**7**) (Table 5) showed cytotoxic effects against KB3.1 cells, exhibiting weak activity with IC_50_ values of 24.1 and 53.6 μM, respectively. These findings suggest that the studied cyclodepsipeptides and tetramic acids generally have a favorable safety profile. This aligns with previous research evaluating the cytotoxicity of certain 3-DTAs, where most derivatives exhibited moderate cytotoxicity against various cancer cell lines, showing no significant difference between *trans*- and *cis*-decalin isomers [31]. Only a limited number of 3-DTAs have been reported to exhibit strong cytotoxic activity against various cell lines [13]. On the other hand, numerous studies have reported diverse cytotoxic properties of cyclodepsipeptides against a wide range of cell lines, highlighting their potential as valuable candidates for further investigation in anticancer drug discovery and development [26, 29].

**Table 5:** Cytotoxicity results of compounds **5** and **7**. All other compounds (**1**–**4** and **6**) were devoid of significant effects up to concentrations of 66.6 µM

Test Cell Line	IC_50_ (µM)		Positive Control Epothilone B (nM)
	5	7	
L929	**	*	0.65
KB3.1	53.6	24.1	0.17

(*): Slight inhibition of cell proliferation, (**): No cytotoxic activity observed

As part of our ongoing search for bioactive molecules, compounds **1**–**7** were tested for nematicidal activity. The nematode assay revealed that only polydosetins A and B (**1** and **2**) (Table 6) exhibited significant activity against *C. elegans*, with corrected mortality rates of 57.6% and 83.1% at 100 µg/mL, respectively. These results suggest that the presence of hydroxyl groups at positions C–6′ and C–7′, combined with a carboxylic acid at C–8′, could play a key role in enhancing nematode mortality. Compounds lacking these features did not demonstrate significant activity (Table S20). Interestingly, compound **2** is the only metabolite among those studied that has a hydrogen atom at C–13 instead of a methyl group. Moreover, it was the only compound that was detected in both *P. karssenii* strains, JKI 73120 and DSM 111209. These findings suggest that the absence of methylation at C–13 in 3-DTAs may enhance nematicidal activity and that compound **2** could play a key role in the ecological function of the studied strains. Nevertheless, further investigations are required to confirm this relationship. In addition, compounds **1** and **2** showed no cytotoxicity in the conducted assays, supporting their potential as promising, safe candidates for nematicidal agent development. To the best of our knowledge, this is the first report of 3-DTAs with nematicidal activity, and only a few other tetramic acids have been previously described with this property [32, 33].

**Table 6:** Results of the nematode assay of compounds **1** and **2** against *C. elegans*

Compound	Corrected mortality rate [%]			Positive control
	100 µg/mL	50 µg/mL	10 µg/mL	
**1**	57.6 ± 10.8	n. a	n. a	93.4 ± 4.1
**2**	83.1 ± 5.2	n. a	n. a	

*n.a.* no significant activity (mortality rate < 50%), Positive control: ivermectin (1 µg/mL)

The mortality rate in the negative control (MeOH) was 14.7 ± 3.5%, and was used to calculate the corrected mortality using the Schneider-Orelli’s formula

Although none of the tested cyclodepsipeptides showed nematicidal activity, the nematicidal compound ophiotine was previously isolated from *P. karssenii* DSM106825, prior to its formal description and taxonomic classification [6, 7]. Ophiotine caused 59% mortality in the host nematode *H. filipjevi* at a concentration of 10 µg/mL [7]. In addition, certain fungal cyclodepsipeptides, particularly those related to the PF1022 series, represent an important class of compounds with proven nematicidal properties, some of which have already been developed into commercial anthelmintics [26, 34]. The cyclodepsipeptide PF1022A, originally isolated from the endophytic fungus *Rosellinia* sp., exhibits nematicidal activity against *Ascaridia galli* in chickens [35, 36]. Its mode of action is complex and involves at least two targets: a latrophilin-like receptor and a Ca^2+^-activated K^+^ channel [26, 37]. A semisynthetic derivative of PF1022A, emodepside, is a pharmacologically active anthelmintic agent used in the treatment of both gastrointestinal and extraintestinal nematodes. Its mechanism of action is similar to that of PF1022A, involving targeted disruption of the nematode neuromuscular function [26, 38].

Cyclodepsipeptides are a diverse class of cyclic peptide natural products characterized by the replacement of one or more amino acid residues in the peptide backbone with hydroxy acids [26, 29, 39]. This modification results in the formation of at least one ester bond, often called a lactone linkage, within the core ring [26, 29, 39]. They are biosynthesized by non-ribosomal peptide synthetases (NRPS) combined with either polyketide synthase (PKS) or fatty acid synthase enzyme systems [26]. Cyclodepsipeptides are widely found in bacteria, fungi, plants, algae, sponges, and other marine organisms [26, 40]. Among the fungal kingdom, these metabolites are mainly reported from the ascomycetous genera *Fusarium*, *Beauveria*, *Isaria*, *Trichoderma*, *Acremonium*, *Aspergillus*, *Penicillium*, and *Rosellinia*, many of which are endophytic in at least part of their lifecycle [26, 29]. Fungal cyclodepsipeptides exhibit a wide range of biological activities, including cytotoxic, phytotoxic, antimicrobial, antiviral, anthelmintic, insecticidal, antimalarial, antitumor, and enzyme-inhibitory effects [26, 41]. Besides PF1022A, other cyclodepsipeptides with pharmaceutical applications include enniatins, which are produced by *Fusarium* spp. [42]. Fusafungine, a mixture of enniatins, was previously employed as an antibacterial nasal spray for the treatment of rhinosinusitis; however, it was withdrawn by the European Medicines Agency in 2016 due to the identification of enniatins as potential mycotoxins with associated health risks [26, 43]. Fungal cyclodepsipeptides are a diverse group of bioactive compounds with significant potential for drug development. Their distinctive structures and wide-ranging biological activities make them promising candidates for therapeutic applications in human health, veterinary medicine, and crop protection, highlighting the importance of continued research on this class of natural products [29, 41]. The biological role of the cyclodepsipeptides pullularins G and H (**6** and **7**) in *P. karssenii* remains unknown, and further research through various bioassays is required to determine their activity and potential applications.

Tetramic acids comprise a diverse class of natural products characterized by a pyrrolidine-2,4-dione core [44]. These compounds are widely distributed in marine and terrestrial organisms, including bacteria, actinobacteria, cyanobacteria, fungi, and sponges [44, 45]. Tetramic acids display a wide range of bioactivities, including antitumor, antibacterial, antifungal, antiprotozoal, and antiviral effects [44, 45]. In addition, certain derivatives, such as macrocidins, function as herbicides [46], while others exhibit antibiofilm activity against bacteria such as *S. aureus* [47, 48]. More than 270 tetramic acid derivatives have been described, exhibiting remarkable structural diversity arising from variations in acylation, hydroxylation, esterification, and cyclization [44, 49]. The biosynthesis of tetramic acids and related 2-pyridones typically involves polyketide synthase-nonribosomal peptide synthetase (PKS-NRPS) hybrid machineries [48]. Comparative analyses of biosynthetic gene clusters (BGCs) involved in the tetramic acid/2-pyridone biosynthesis have revealed that, in addition to the core PKS–NRPS hybrid genes, these clusters may also contain varying numbers of oxidoreductases, tailoring genes, transporters, transcription factors, and other regulatory elements [50]. This genomic complexity underlies the remarkable structural diversity and evolutionary dynamics of these biosynthetic pathways [48, 51].

Tetramic acids can be classified into eight groups based on their structural features and biosynthetic pathways. These groups include simple 3-acyl-tetramic acids, 3-oligoenoyltetramic acids, 3-decalinoyltetramic acids (3-DTAs), 3-spirotetramic acids, macrocyclic tetramic acids, *N*-acylated tetramic acids, α-cyclopiazonic acid-type tetramic acids, and other tetramic acids [45]. Polydosetins A–E (**1**–**5**) belong to the 3-DTAs class, which are primarily produced by filamentous fungi and are widespread among ascomycetes. [16, 31]. This class of compounds exhibits diverse biological activities, including antibacterial, antifungal, antiviral, and antihyperlipidemic properties [31]. The structural diversity of these compounds is generated through fungal biosynthetic pathways involving PKS-NRPS and decalin synthase [13, 31]. The polyketide moiety forms the decalin ring via an intramolecular Diels–Alder cycloaddition, while the heterocyclic pyrrolidine-2-one moiety, derived from amino acids, connects to the polyketide-derived decalin ring [13].

Several 3-DTAs share structural similarities with compounds **1**–**5**, including equisetin, trichosetin, beauversetin, Sch 210972, and Sch 213766. These compounds exhibit diverse bioactivities, ranging from antimicrobial effects to anti-HIV properties, highlighting their potential as lead compounds in drug discovery [13]. Equisetin was the first identified member of the 3-DTAs class derived from fungi. It was first isolated in 1974 from *Fusarium equiseti* [52]. Equisetin exhibits phytotoxicity [53] and has been reported as an inhibitor of human immunodeficiency virus type 1 (HIV-1) integrase [54]. Additionally, it shows cytotoxic activity against all NCI-60 cell lines, particularly against human leukemic lymphoblast CCRF-CEM cells [54]. Trichosetin, a derivative of equisetin, was initially isolated from dual cultures of *Trichoderma harzianum* and *Catharanthus roseus* [55]. It is also produced by *F. oxysporum* and has shown antimicrobial activity against *S. aureus* (MRSA) and *B. subtilis* [15, 55]. Beauversetin, a phomasetin-type 3-DTA, was first reported from *Beauveria bassiana* isolated from the marine sponge *Myxilla incrustans* [56]. Beauversetin has shown moderate cytotoxic activity against a panel of six cell lines [56]. Sch 210972 and Sch 213766 are also phomasetin-type 3-DTAs and were isolated from the fungus *Chaetomium globosum* [57, 58]. These compounds displayed inhibitory activities in a CCR-5 membrane binding assay, suggesting their potential as inhibitors of the chemokine receptor CCR-5, considered a therapeutic target for antiviral agents [57, 58]. The diverse biological activities of the 3-DTAs discussed above, which share structural similarities with polydosetins A–E (**1**–**5**), suggest that these compounds may have additional activities beyond those reported here and therefore merit further investigation. Moreover, the mode of action of 3-DTAs compounds is not well understood, highlighting an important area for future research.

## Conclusions

The current study evaluated the potential of the endophytic and nematode-associated fungus *P. karssenii* strains JKI 73120 and DSM 111209 to produce secondary metabolites. Building on previous research by Helaly et al. (2018), which reported five compounds from this fungus, we herein report seven additional metabolites that are novel to science, the tetramic acids polydosetins A–E (**1**–**5**) and the cyclodepsipeptides pullularins G and H (**6** and **7**). Bioactivity tests revealed that compounds **1**, **3**, **5**, and **7** demonstrated antimicrobial effects against Gram-positive bacterial strains, with compounds **3** and **5** also exhibiting activity against fungi. Importantly, the tetramic acids **1** and **2**, belonging to the 3-DTA class, are the first compounds in this class reported to exhibit nematicidal activity. Hydroxyl groups at positions C–6′ and C–7′, together with a carboxylic acid at C–8′, may play a key role in increasing nematode mortality. Additionally, the absence of methylation at C–13, as observed in compound **2**—which was the only metabolite produced by both studied strains—may further enhance nematicidal activity. Compounds **1** and **2** showed no cytotoxicity, highlighting their potential as biocontrol agents. These findings emphasize the impressive chemical diversity of nematode-associated fungi and their potential as sources of bioactive compounds for developing novel strategies to control nematodes and microbial pathogens.

## Supplementary Information

Additional file1 (PDF 8566 KB)

## Data Availability

The data that support the findings of this study are available in the supplementary information of this article.
