# Supplementary material for: Polydosetins & pullularins—bioactive tetramic acids & cyclodepsipeptides from the endophytic and nematophagous fungus Polydomus karssenii
Source: Nat Prod Bioprospect. 2026 Feb 3;16(1):28. doi: 10.1007/s13659-025-00579-8 (PMC12864567; doi:10.1007/s13659-025-00579-8)
Supplement: Supplementary file 1 — Additional file1 (PDF 8566 KB) [file 13659_2025_579_MOESM1_ESM.pdf]

Supplementary Information for

Polydosetins & Pullularins – Bioactive Tetramic Acids & Cyclodepsipeptides from the Endophytic and Nematophagous Fungus *Polydomus karssenii*

Natalia A. Llanos-López[1,2,#], Jan-Peer Wennrich[1,2,‡,#], Janette Miled[1,2], Samad Ashrafi[3,4], Wolfgang Maier[3], Frank Surup[1,2], and Marc Stadler[1,2,*]

1 Department of Microbial Drugs, Helmholtz Centre for Infection Research (HZI) and German Centre for Infection Research (DZIF), DZIF Partner Site Hannover-Braunschweig, Germany, Inhoffenstrasse 7, 38124 Braunschweig, Germany

2 Institute of Microbiology, Technische Universität Braunschweig, Spielmannstraße 7, 38106 Braunschweig, Germany

3 Institute for Epidemiology and Pathogen Diagnostics, Julius Kühn Institut (JKI) - Federal Research Centre for Cultivated Plants, Messeweg 11–12, 38104 Braunschweig, Germany

4 Department of Zoology and Entomology, University of the Free State, Bloemfontein 9300, South Africa

‡ Present address: Laboratory of Fungal Genetics and Metabolism, Institute of Microbiology of the Czech Academy of Sciences, Prague 14220, Czechia

* Correspondence: marc.stadler@helmholtz-hzi.de (M.S)

\# These authors contributed equally

Contents of Supporting Information

Figure S1. LR-ESI-MS spectrum of polydosetin A (**1**). 5

Figure S2. HR-ESI-MS spectrum of polydosetin A (**1**). 6

Table S1. ^{13}C NMR data of polydosetin A (**1**) in various deuterated solvents. Depicted are the shifts of the main tautomer. 7

Table S2. ^{1}H NMR data of polydosetin A (**1**) in various deuterated solvents. Depicted are the shifts of the main tautomer. 8

Figure S3. ^{1}H NMR spectrum of polydosetin A (**1**) (500 MHz, acetone–*d6*). 9

Figure S4. ^{13}C NMR spectrum of polydosetin A (**1**) (125 MHz, acetone–*d6*). 10

Figure S5. COSY NMR spectrum of polydosetin A (**1**) (500 MHz, acetone–*d6*). 11

Figure S6. HSQC NMR spectrum of polydosetin A (**1**) (500 MHz, acetone–*d6*). 12

Figure S7. HMBC NMR spectrum of polydosetin A (**1**) (500 MHz, acetone–*d6*). 13

Figure S8. ^{1}H NMR spectrum of polydosetin A (**1**) (700 MHz, CH_3OH–*d4*). 14

Figure S9. ^{13}C NMR spectrum of polydosetin A (**1**) (175 MHz, CH_3OH–*d4*). 15

Figure S10. LR-ESI-MS spectrum of polydosetin B (**2**). 16

Figure S11. HR-ESI-MS spectrum of polydosetin B (**2**). 17

Figure S12. ^{1}H NMR spectrum of polydosetin B (**2**) (500 MHz, CH_3OH–*d4*). 18

Figure S13. ^{13}C NMR spectrum of polydosetin B (**2**) (125 MHz, CH_3OH–*d4*). 19

Figure S14. COSY NMR spectrum of polydosetin B (**2**) (500 MHz, CH_3OH–*d4*). 20

Figure S15. HSQC NMR spectrum of polydosetin B (**2**) (500 MHz, CH_3OH–*d4*). 21

Figure S16. HMBC NMR spectrum of polydosetin B (**2**) (500 MHz, CH_3OH–*d4*). 22

Figure S17. LR-ESI-MS spectrum of polydosetin C (**3**). 23

Figure S18. HR-ESI-MS spectrum of polydosetin C (**3**). 24

Figure S19. ^{1}H NMR spectrum of polydosetin C (**3**) (500 MHz, CH_3OH–*d4*). 25

Figure S20. ^{13}C NMR spectrum of polydosetin C (**3**) (175 MHz, CH_3OH–*d4*). 26

Figure S21. COSY NMR spectrum of polydosetin C (**3**) (700 MHz, CH_3OH–*d4*). 27

Figure S22. HSQC NMR spectrum of polydosetin C (**3**) (700 MHz, CH_3OH–*d4*). 28

Figure S23. HMBC NMR spectrum of polydosetin C (**3**) (500 MHz, CH_3OH–*d4*). 29

Figure S24. LR-ESI-MS spectrum of polydosetin D (**4**). 30

Figure S25. ^{1}H NMR spectrum of polydosetin D (**4**) (500 MHz, CH_3OH–*d4*). 31

Figure S26. ^{13}C NMR spectrum of polydosetin D (**4**) (125 MHz, CH_3OH–*d4*). 32

Figure S27. COSY NMR spectrum of polydosetin D (**4**) (500 MHz, CH_3OH–*d4*). 33

Figure S28. HSQC NMR spectrum of polydosetin D (**4**) (500 MHz, CH_3OH–*d4*). 34

Figure S29. HMBC NMR spectrum of polydosetin D (**4**) (500 MHz, CH_3OH–*d4*). 35

Figure S30. LR-ESI-MS spectrum of polydosetin E (**5**). 36

Figure S31. HR-ESI-MS spectrum of polydosetin E (**5**).. 37
Figure S32. ^{1}H NMR spectrum of polydosetin E (**5**) (500 MHz, CH_3OH–*d4*)....................... 38
Figure S33. ^{13}C NMR spectrum of polydosetin E (**5**) (125 MHz, CH_3OH–*d4*)...................... 39
Figure S34. COSY NMR spectrum of polydosetin E (**5**) (500 MHz, CH_3OH–*d4*). 40
Figure S35. HSQC NMR spectrum of polydosetin E (**5**) (500 MHz, CH_3OH–*d4*). 41
Figure S36. HMBC NMR spectrum of polydosetin E (**5**) (500 MHz, CH_3OH–*d4*). 42
Figure S37. 1D TOCSY NMR spectrum (700 MHz, pyridine–d_5) of the **3**-(*S*)-MTPA bisester with irradiation on H–6' at δ_H 6.16 ppm. 43
Figure S38. 1D TOCSY NMR spectrum (700 MHz, pyridine–*d5*) of the **3**-(*R*)-MTPA bisester with irradiation on H–6' at 43
Figure S39. COSY (blue arrows) and HMBC (green arrows) correlations utilized in the structure elucidation process of **1** 44
Figure S40. Experimental ECD spectrum of polydosetins A–E (**1**–**5**) in methanol................ 44
Figure S41. LR-ESI-MS spectrum of pullularin G (**6**)... 45
Figure S42. HR-ESI-MS spectrum of pullularin G (**6**). 46
Figure S43. ^{1}H NMR spectrum of pullularin G (**6**) (500 MHz, DMSO-*d6*). 47
Figure S44. ^{13}C NMR spectrum of pullularin G (**6**) (125 MHz, DMSO-*d6*). 48
Figure S45. COSY NMR spectrum of pullularin G (**6**) (500 MHz, DMSO-*d6*)..................... 49
Figure S46. HSQC NMR spectrum of pullularin G (**6**) (500 MHz, DMSO-*d6*)..................... 50
Figure S47. HMBC NMR spectrum of pullularin G (**6**) (500 MHz, DMSO-*d6*).................... 51
Figure S48. LR-ESI-MS spectrum of pullularin H (**7**)... 52
Figure S49. HR-ESI-MS spectrum of pullularin H (**7**). 53
Figure S50. ^{1}H NMR spectrum of pullularin H (**7**) (700 MHz, DMSO-*d6*). 54
Figure S51. APT NMR spectrum of pullularin H (**7**) (175 MHz, DMSO-*d6*)........................ 55
Figure 52. COSY NMR spectrum of pullularin H (**7**) (700 MHz, DMSO-*d6*)....................... 56
Figure S53. HSQC NMR spectrum of pullularin H (**7**) (700 MHz, DMSO-*d6*)..................... 57
Figure S54. HMBC NMR spectrum of pullularin H (**7**) (700 MHz, DMSO-*d6*).................... 58
Figure S55. Key COSY (blue arrows) and HMBC (green arrows) accounting for the structure elucidation of pullularins G and H (**6** and **7**)... 59
Figure S56. Experimental ECD spectrum of pullularin G (**6**) in methanol............................ 59
Figure S57. Experimental ECD spectrum of pullularin H (**7**) in methanol............................ 60
Figure S58. LC-ESI-MS spectra of pullularins G and H (**6** and **7**), N-methyl-L-leucine, and N-methyl-D-leucine with FDAA. 60
Figure S59. LC-ESI-MS spectra of pullularins G and H (**6** and **7**), DL-serine, and L-serine with FDAA. 61
Figure S60. LC-ESI-MS spectra of pullularins G and H (**6** and **7**), D-proline, and L-proline with FDAA. 61

Figure S61. LC-ESI-MS spectra of pullularins G and H (**6** and **7**), and N-methyl-L-isoleucine with FDAA. 62

Isolation procedure of compounds from *P. karssenii* strain JKI 73120 62

Scheme S1. Separation scheme of the isolated compounds **2**, **3**, and **5**–**7** from *P. karssenii* strain JKI 73120. 65

Table S3. Chromatographic separation parameters for the BRFT + WOFT methanol extract of *P. karssenii* strain JKI 73120. 65

Table S4. Chromatographic separation parameters for fraction F8 of *P. karssenii* strain JKI 73120 (BRFT + WOFT methanol extract). 66

Table S5. Chromatographic separation parameters for fraction F16 (from F08, BRFT + WOFT methanol extract of *P. karssenii* strain JKI 73120). 66

Table S6. Chromatographic separation parameters for fraction F17 (from F08, BRFT + WOFT methanol extract of *P. karssenii* strain JKI 73120). 66

Table S7. Chromatographic separation parameters for fraction F4 (from F17 → F08, BRFT + WOFT MeOH extract of *P. karssenii* strain JKI 73120). 67

Table S8. Chromatographic separation parameters for the BRFT + WOFT *n*-heptane extract of *P. karssenii* strain JKI 73120. 67

Table S9. Chromatographic separation parameters for fraction G8+G11 of *P. karssenii* strain JKI 73120 (BRFT + WOFT *n*-heptane extract). 68

Table S10. Chromatographic separation parameters for fraction G8 (from G8+G11, BRFT + WOFT *n*-heptane extract of *P. karssenii* strain JKI 73120). 68

Table S11. Chromatographic separation parameters for fraction G9 of *P. karssenii* strain JKI 73120 (BRFT + WOFT *n*-heptane extract). 68

Scheme S2. Separation scheme of the isolated compounds **1**, **2**, and **4** from *P. karssenii* strain DSM 111209. 69

Table S12. Chromatographic separation parameters for the mycelial extract of *P. karssenii* strain DSM 111209. 69

Table S13. Chromatographic separation parameters for fraction F10 of *P. karssenii* strain DSM 111209 (mycelial extract). 70

Table S14. Chromatographic separation parameters for fraction F10 (from F10, mycelial extract of *P. karssenii* strain DSM 111209). 70

Table S15. Chromatographic separation parameters for the supernatant extract of *P. karssenii* strain DSM 111209. 70

Table S16. Chromatographic separation parameters for fraction G9 of *P. karssenii* strain DSM 111209 (supernatant extract). 71

Table S17. Chromatographic separation parameters for fraction G10 of *P. karssenii* strain DSM 111209 (supernatant extract). 71

Table S18. Cytotoxicity (IC_{50}) of compounds **1**–**7**. 71

Table S19. Antimicrobial activity (MIC) of compounds **1**–**7**. 72

Table S20. Nematicidal activity of compounds **1**–**7**. 72

Generic Display Report

Analysis Info

Analysis Name	D:\MIS DOCUMENTOS\Downloads\Compounds 1st-20250801T235501Z-1-001\Compounds 1st\MyNe_03_26_01_X_F10_I_F2_461 (1)\Amazon\MyNe_03_26_01_X_F10_I_F2_GB4_01_11404.d	Acquisition Date	16/06/2022 8:17:14 a. m.
Method	Screening_Set05iso-uv2f10_11404.m	Operator	Lab
Sample Name	MyNe_03_26_01_X_F10_I_F2	Instrument	amaZon speed
Comment			

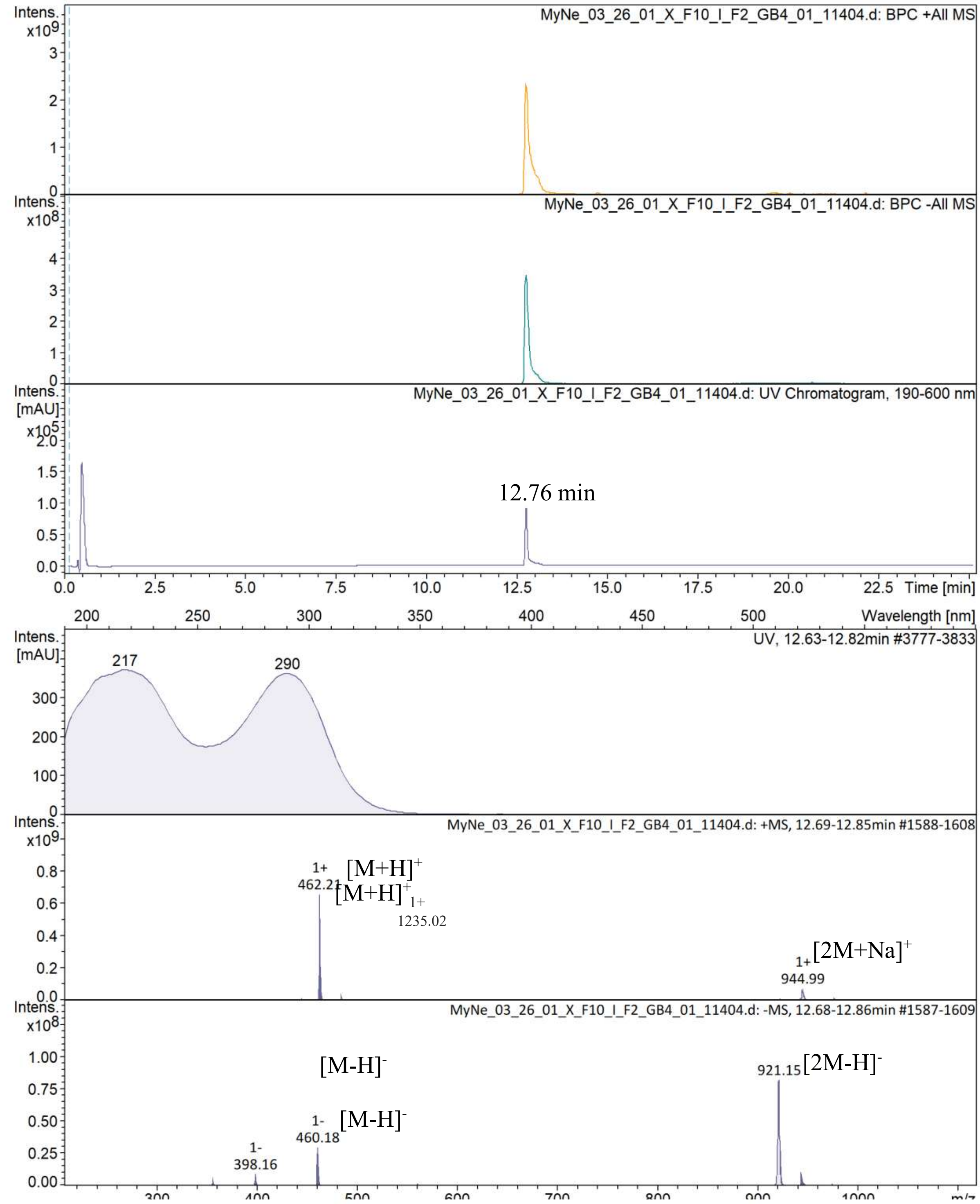

Figure S1. LR-ESI-MS spectrum of polydosetin A (**1**).

Generic Display Report

Analysis Info

		Acquisition Date	06-Mar-25 8:16:21 PM
Analysis Name	S:\DATA\timsTOF\NLL21_Natalia Llanos\25_03\MyNe_03_26_01_X_F10_I_F2_P1-B-4_1_5756.d		
Method	MWIS_BEH50mm_25min_IntThreshold 225.m	Operator	Admin
Sample Name	MyNe_03_26_01_X_F10_I_F2	Instrument	timsTOF Pro 2
Comment			

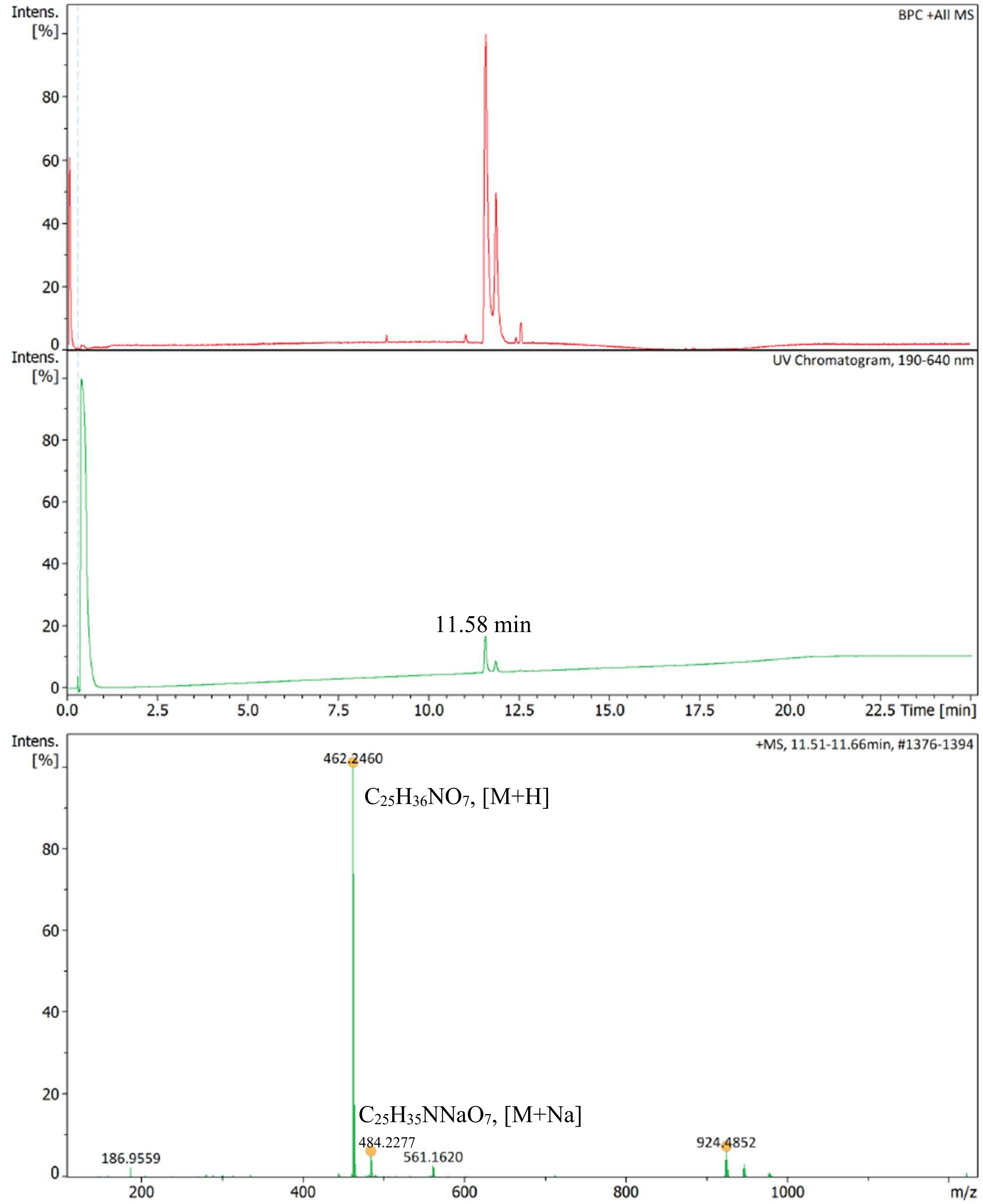

Bruker Compass DataAnalysis 6.1 printed: 01-Aug-25 4:06:22 PM by: nll21 Page 1 of 1

Figure S2. HR-ESI-MS spectrum of polydosetin A (**1**).

Table S1. ^{13}C NMR data of polydosetin A (**1**) in various deuterated solvents. Depicted are the shifts of the main tautomer.

pos.	mult.	$CH_3OH–d_4$ [a]	acetone–d_6 [b]	$CH_3Cl–d$ [b]	pyridine–d_5 [a,d]	DMSO–d_6 [a,d]
1	C	201.6	199.2	201.7	200.6	198.0
2	C	49.6	50.7	51.0	51.8	49.8
3	CH	57.5	56.3	56.2	56.8	55.0
4	C	133.0	131.9	131.4	132.8	131.0
5	CH	128.2	127.1	126.7	127.6	126.4
6	CH	33.7	32.6	32.1	33.2	31.7
7	CH_2	41.2	40.1	39.8	40.5	39.2
8	CH	29.5	28.3	27.8	28.7	27.2
9	CH_2	34.1	33.0	32.7	33.6	32.2
10	CH_2	24.5	23.5	23.4	24.2	22.6
11	CH	43.2	41.9	41.5	42.6	40.9
12	CH_3	15.7	14.9	14.9	15.7	14.5
13	C	136.0	134.9	134.5	134.9	134.1
14	CH	125.9	124.6	124.7	126.1	123.5
15	CH_3	13.8	13.2	13.5	14.2	13.3
16	CH_3	19.4	18.6	18.8	19.4	18.5
17	CH_3	23.0	22.4	22.5	23.3	22.3
18	CH_3	14.7	13.9	13.6	n.o.	n.o.
2’	C	n.o.	180.4	n.o.	n.o.	n.o.
3’	C.	n.o.	101.3	n.o.	n.o.	n.o.
4’	C	n.o.	191.6	n.o.	193.3	n.o.
5’	CH	63.2	62.8	n.o.	63.6	61.9[c]
6’	CH	72.8	72.2	72.3	74.5	70.6[c]
7’	CH	74.5	73.3	73.3	73.6	71.3[c]
8’	C	176.0	173.6	179.2	177.0	173.9

n.o. not observed, [a] 175 MHz, [b] 125 MHz, [c] assignment insecure, [d] chemical shifts extracted from HSQC and HMBC data.

Table S2. ^{1}H NMR data of polydosetin A (**1**) in various deuterated solvents. Depicted are the shifts of the main tautomer.

Pos	CH_3OH–d_4 [a]	acetone–d_6 [b]	CH_3Cl–d [b]	pyridine–d_5 [a]	DMSO–d_6 [a]
3	3.30, br s	3.25, br s	3.13, m	3.81, br s	3.12, br s
5	5.22, s	5.23, m	5.20, d (10.8)	5.27, s	5.21, d (9.7)
6	2.07, m	2.08, m	2.03, m	2.17, m	2.00, m
7	1.64, m	1.64, m	1.60, m	1.66, m	1.59, m
	1.47, m		1.45, m	1.56, m	1.38, m
8	2.07, m	2.07, m	2.07, m	2.04, m	2.03, m
9	1.69, m	1.67, m	1.66, m	1.75, m	1.57, m
	1.54, br d (13.0)	1.53, m	1.51, m	1.54, m	1.47, m
10	1.62, m	1.65, m	1.54, m	1.88, m	1.52, m
	1.22, m	1.23, m	1.19, m	1.34, m	1.12, m
11	1.89, br t (10.2)	1.88, t (10.4)	1.84, m	2.16, m	1.75, m
12	1.49, s	1.50, m	1.47, s	1.91, s	1.42, s
14	5.25, br s	5.23, m	5.17, m	5.93, m	5.25, q (6.2)
15	1.46, m	1.44, d (6.7)	1.43, m	1.56, d (6.5)	1.37, m
16	1.05, d (7.3)	1.04, d (7.3)	1.02, m	1.04, d (7.2)	0.99, dd (7.2,1.8)
17	1.50, br s	1.50, m	1.49, m	1.61, s	1.46, br s
18	1.46, m	1.47, br s	1.43, m	1.72, s	1.39, m
1’NH	n.o.	7.85, br s	n.o.	n.o.	n.o.
5’	n.o.	4.02, br s	3.49, m	4.93, m	3.86, m [c]
6’	4.17, dd (6.0,2.0)	4.24, dd (6.0,2.1)	4.47, m	5.06, br dd (7.2,2.9)	4.38, br s [c]
7’	4.23, d (6.0)	4.38, d (6.0)	4.08, m	5.55, m	3.88, d (8.8) [c]

n.o. not observed, [a] 700 MHz, [b] 500 MHz, [c] assignment insecure.

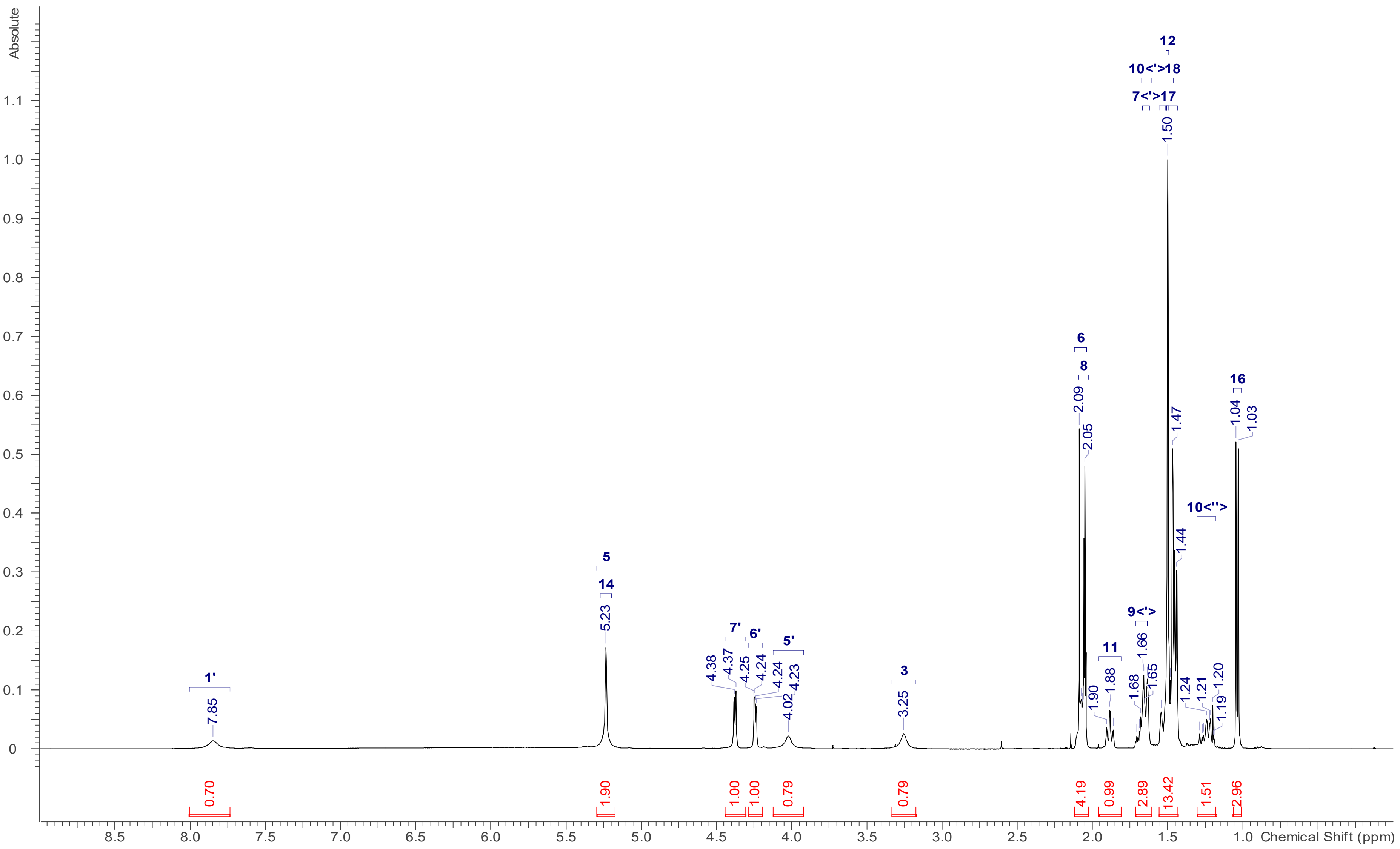

Figure S3. ^{1}H NMR spectrum of polydosetin A (**1**) (500 MHz, acetone–*d6*).

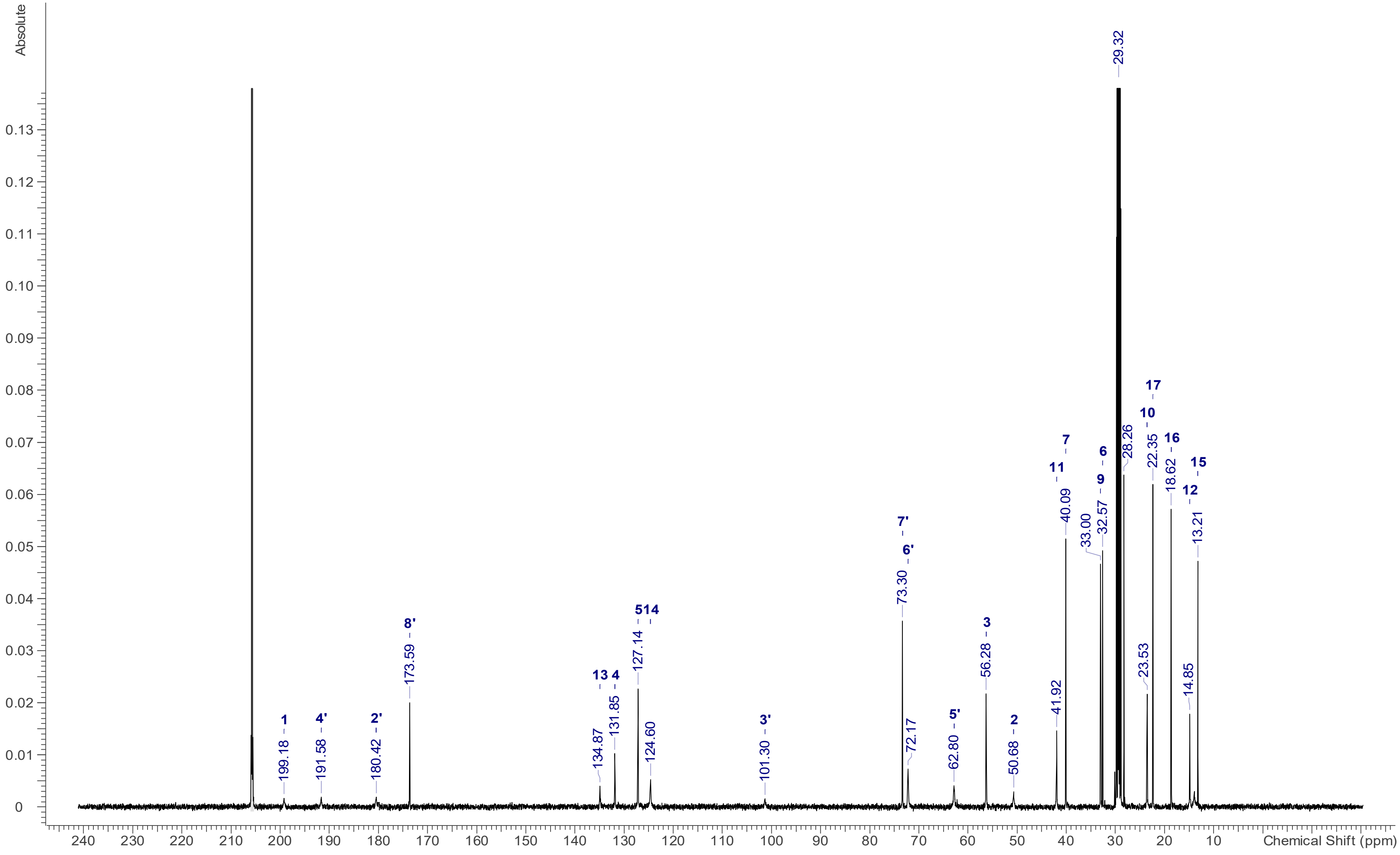

Figure S4.^{13}C NMR spectrum of polydosetin A (**1**) (125 MHz, acetone–*d6*).

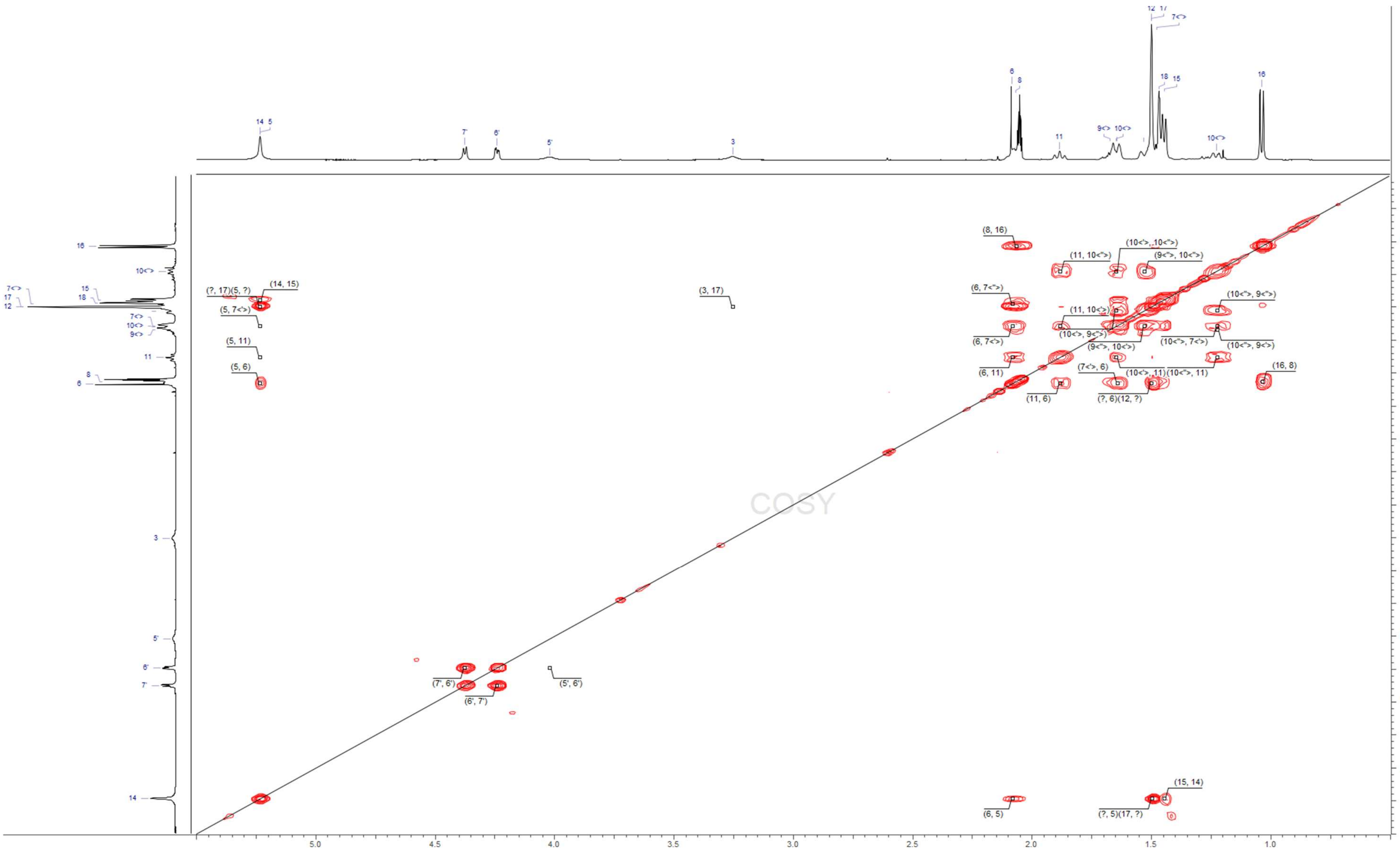

Figure S5. COSY NMR spectrum of polydosetin A (**1**) (500 MHz, acetone–*d6*).

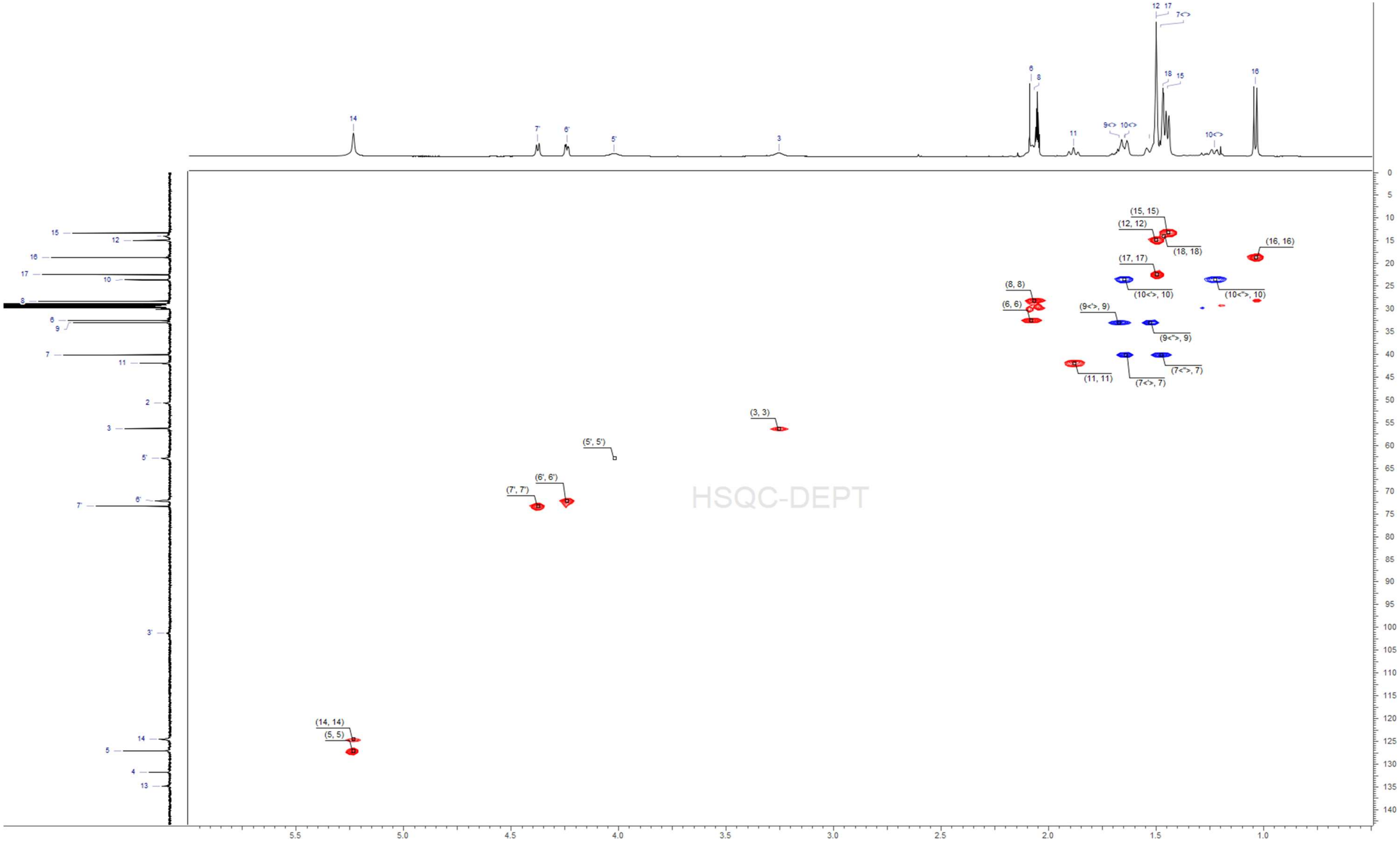

Figure S6. HSQC NMR spectrum of polydosetin A (**1**) (500 MHz, acetone–*d6*).

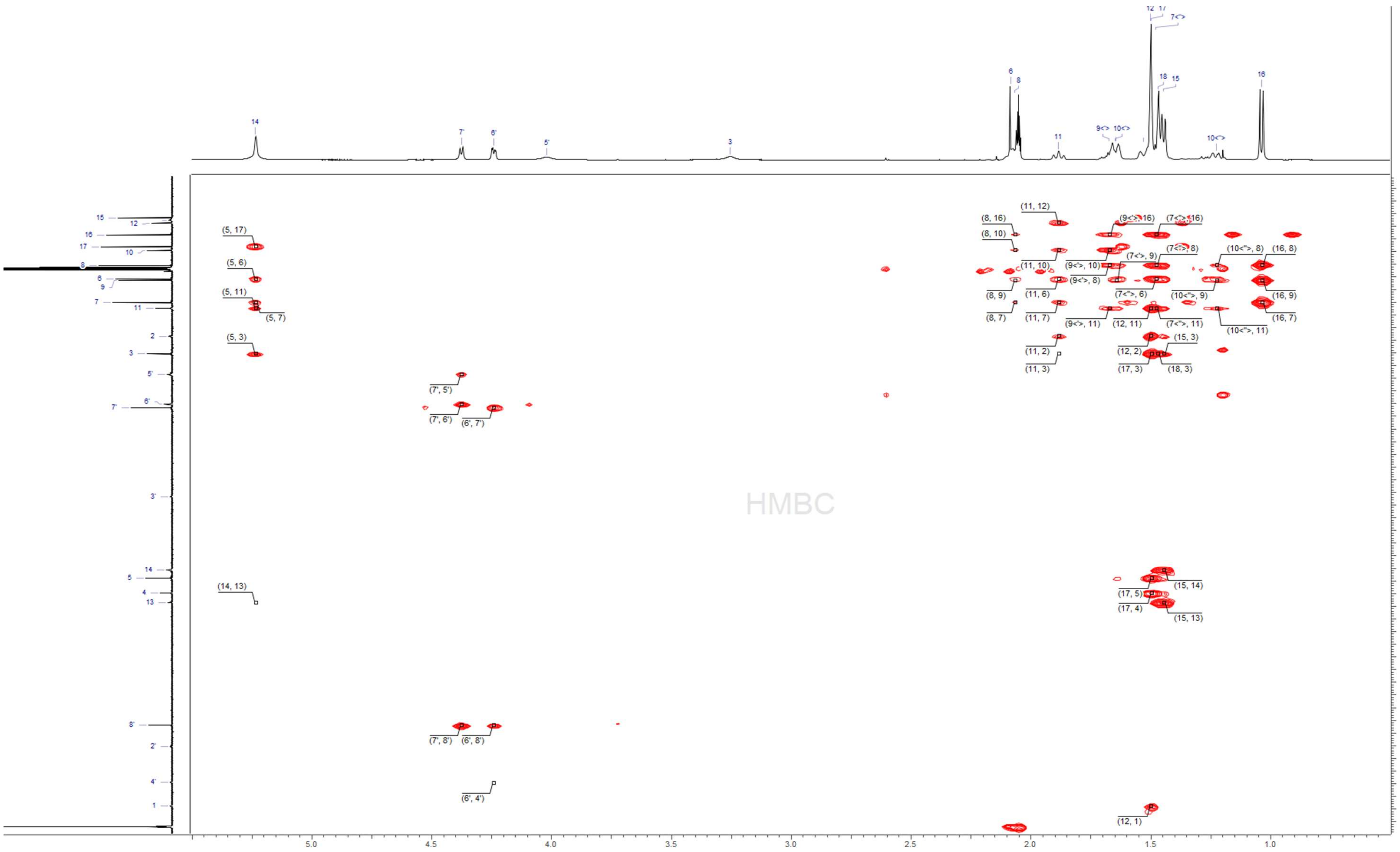

Figure S7. HMBC NMR spectrum of polydosetin A (**1**) (500 MHz, acetone–*d6*).

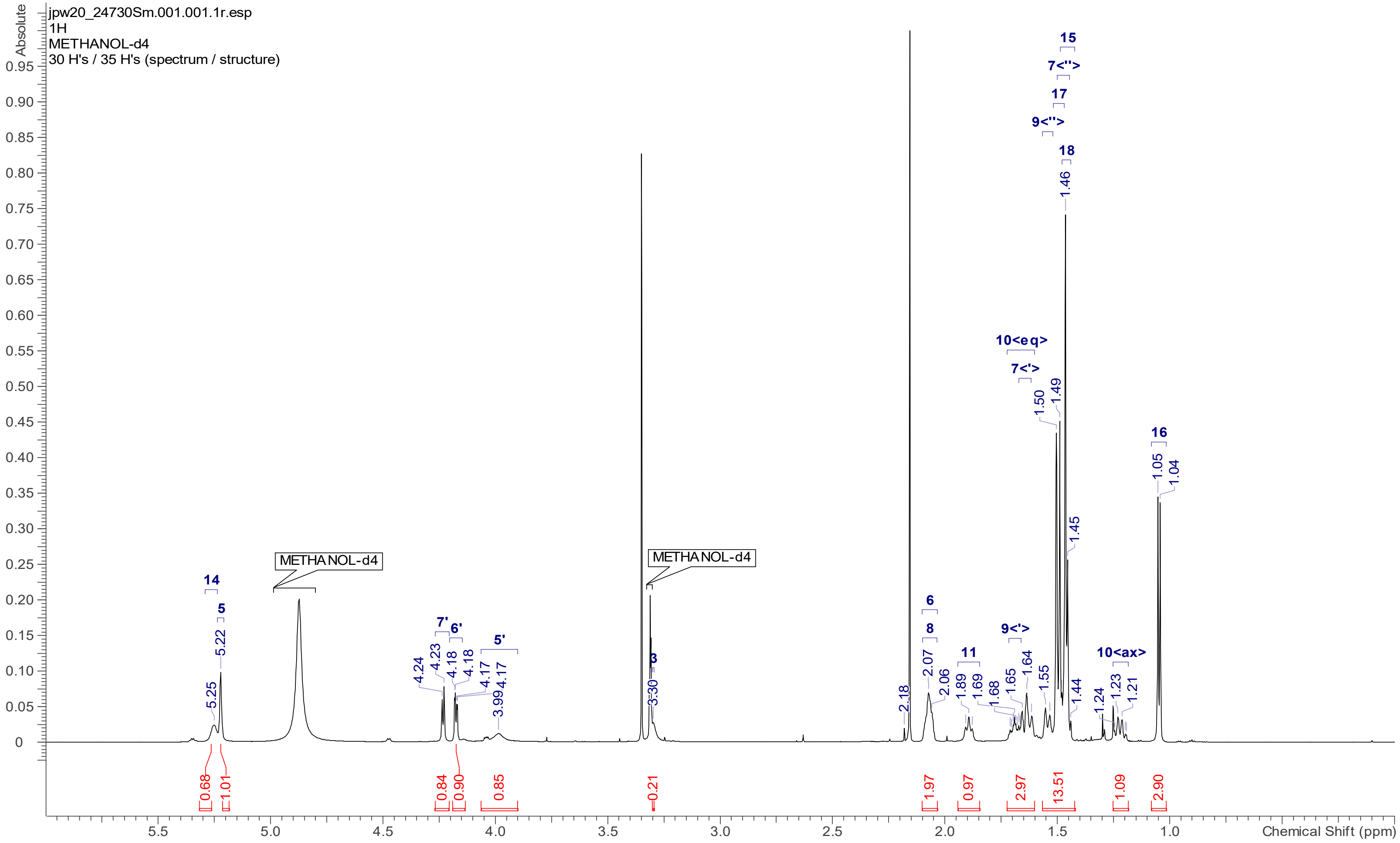

Figure S8. ^{1}H NMR spectrum of polydosetin A (**1**) (700 MHz, CH_3OH–*d4*).

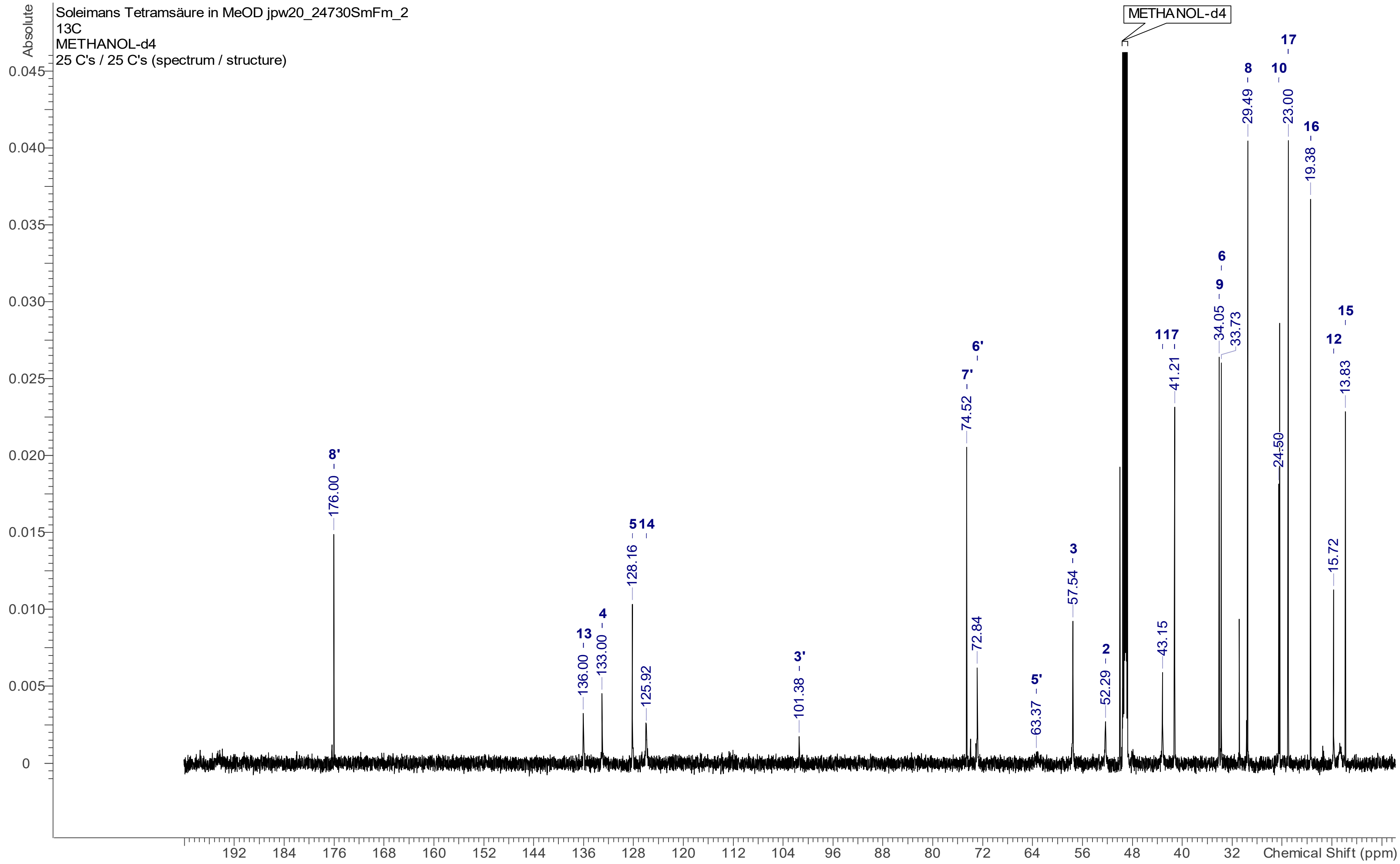

Figure S9. ^{13}C NMR spectrum of polydosetin A (**1**) (175 MHz, CH_3OH–*d4*).

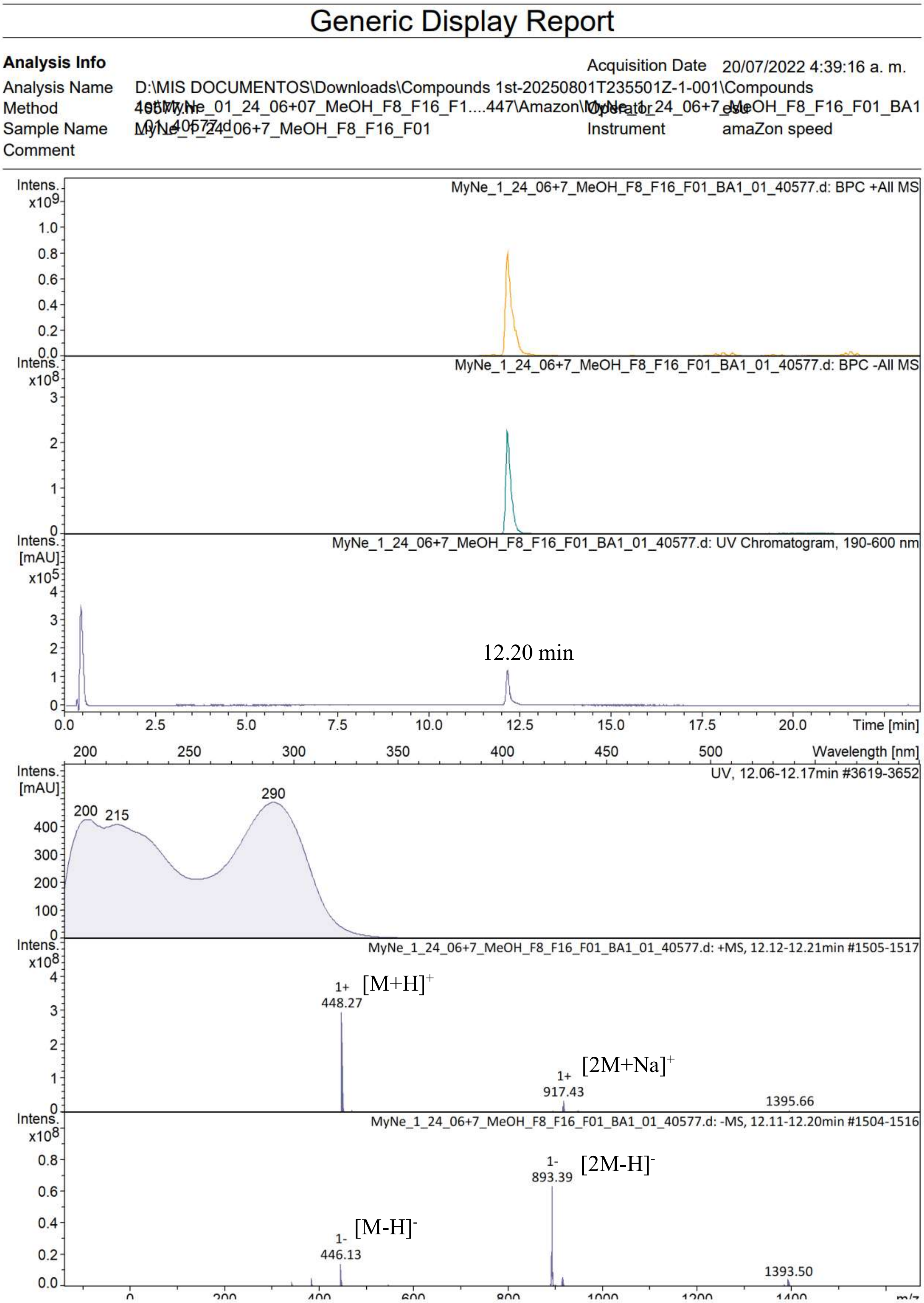

Figure S10. LR-ESI-MS spectrum of polydosetin B (**2**).

Generic Display Report

Analysis Info

Analysis Name	S:\DATA\timsTOF\NLL21_Natalia Llanos\25_03\MyNe_03_26_01_X_F9_F1_P1-B-3_1_5755.d	Acquisition Date	06-Mar-25 7:45:24 PM
Method	MWIS_BEH50mm_25min_IntThreshold 225.m	Operator	Admin
Sample Name	MyNe_03_26_01_X_F9_F1	Instrument	timsTOF Pro 2
Comment			

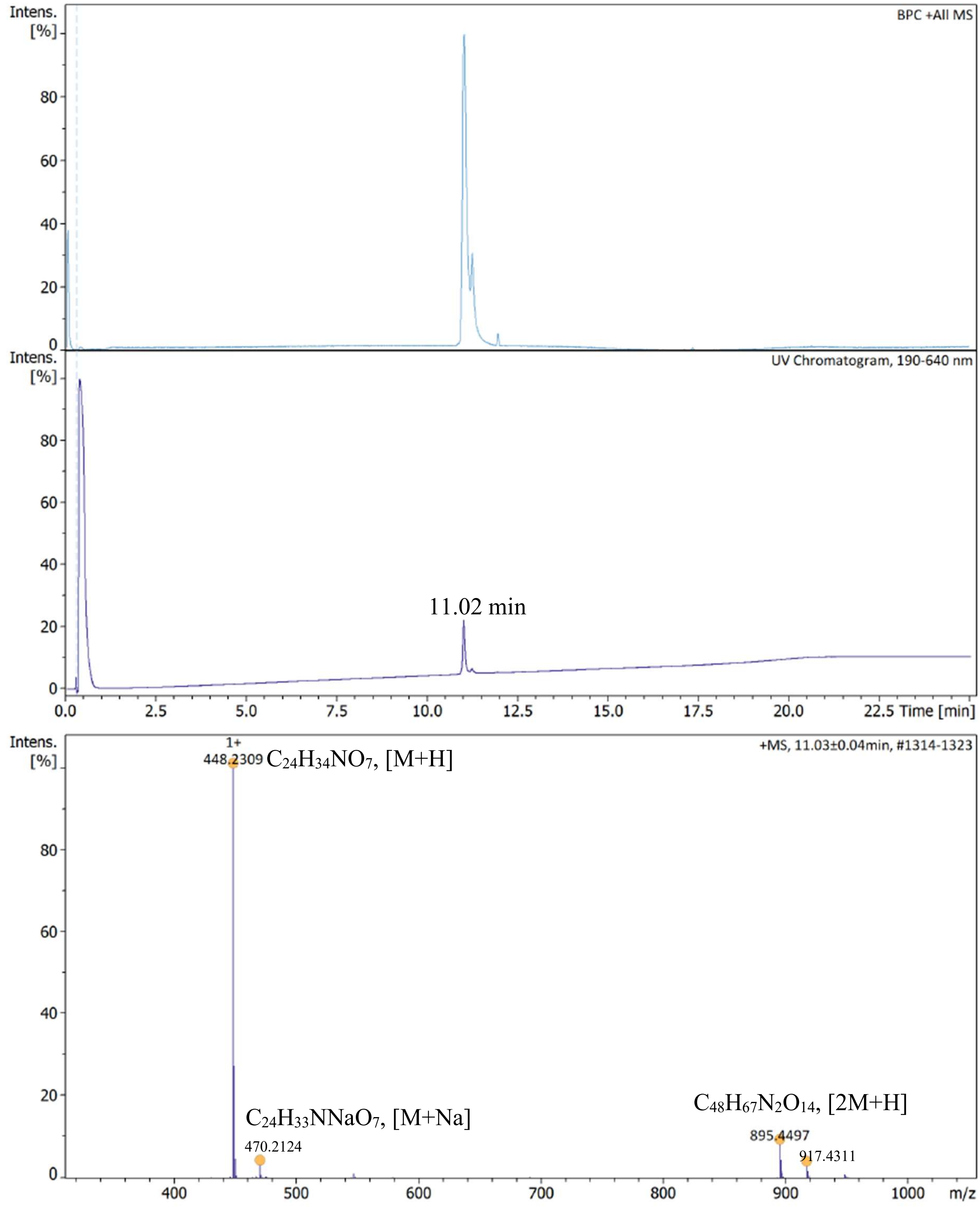

Bruker Compass DataAnalysis 6.1 printed: 01-Aug-25 4:46:20 PM by: nll21 Page 1 of 1

Figure S11. HR-ESI-MS spectrum of polydosetin B (**2**).

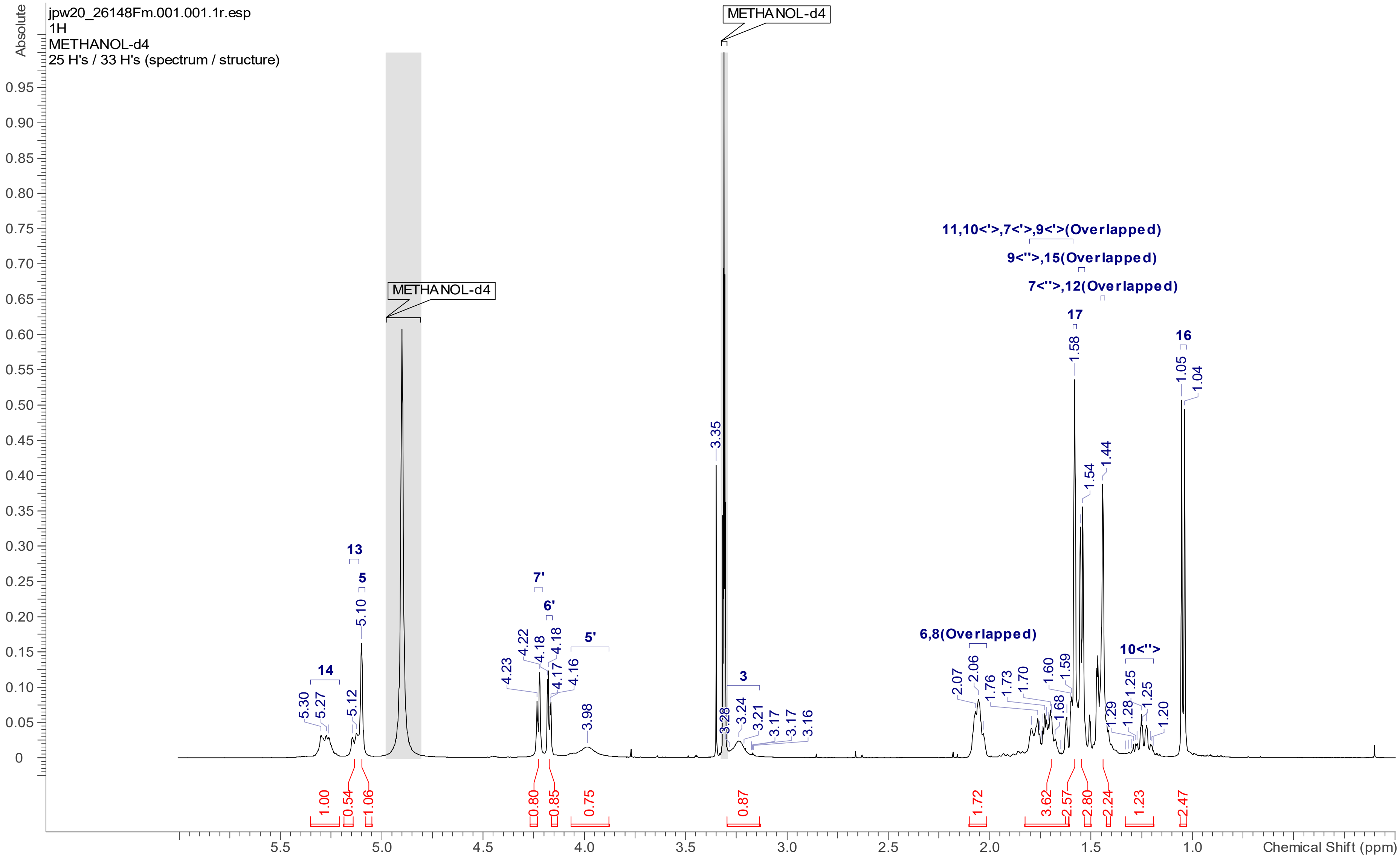

Figure S12. ^{1}H NMR spectrum of polydosetin B (**2**) (500 MHz, CH_3OH–*d4*).

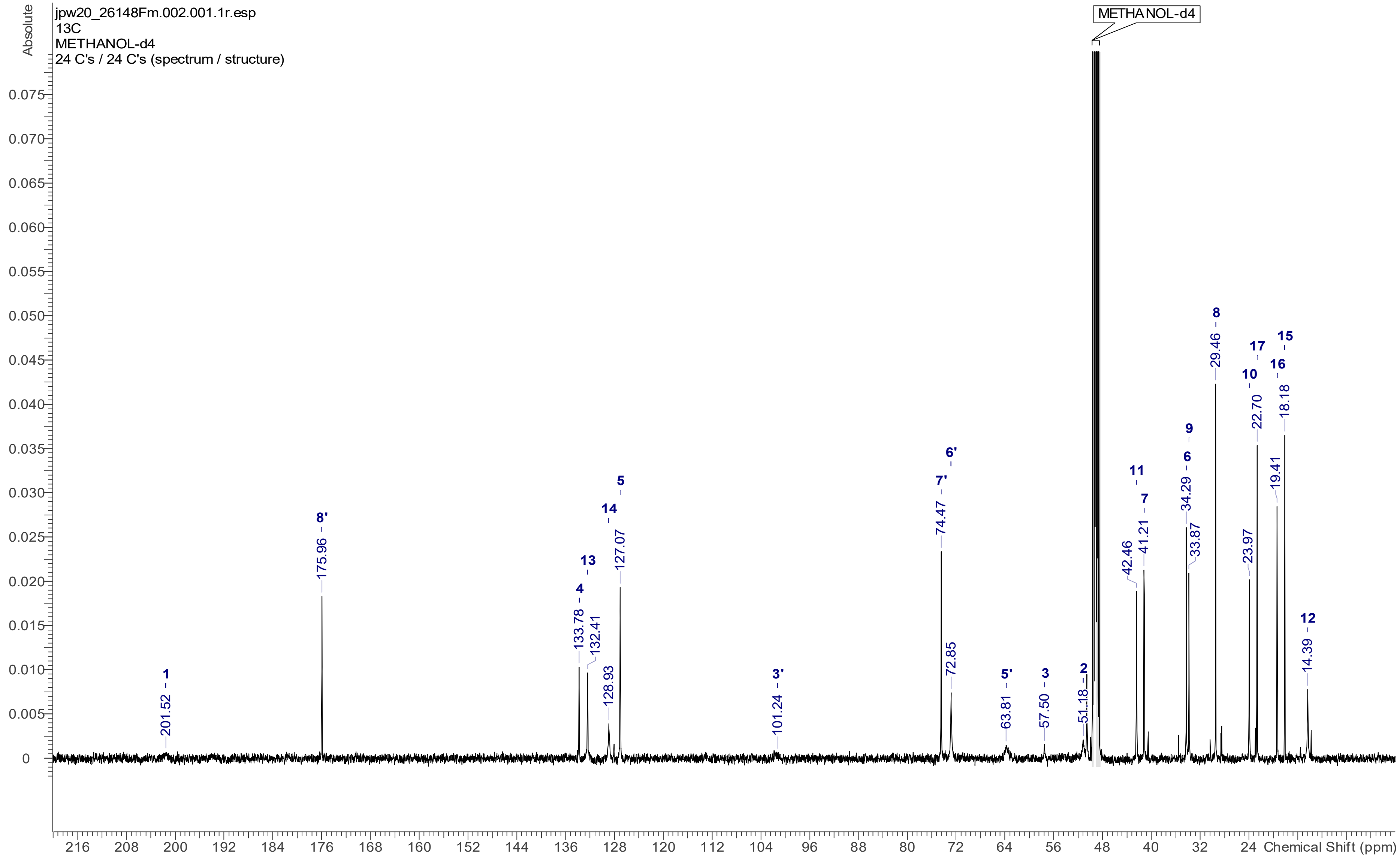

Figure S13. ^{13}C NMR spectrum of polydosetin B (**2**) (125 MHz, CH_3OH–*d4*).

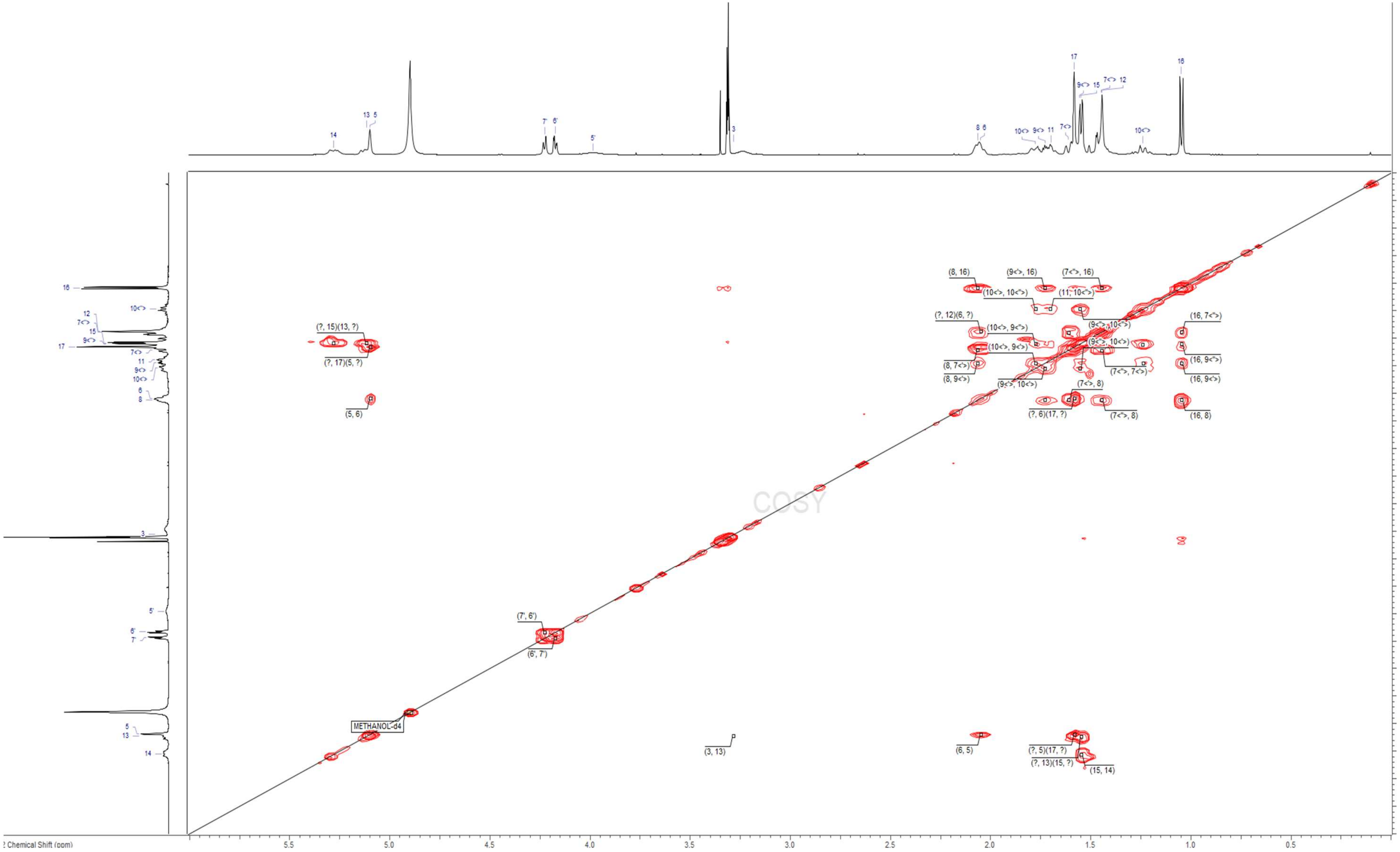

Figure S14. COSY NMR spectrum of polydosetin B (**2**) (500 MHz, CH_3OH–*d4*).

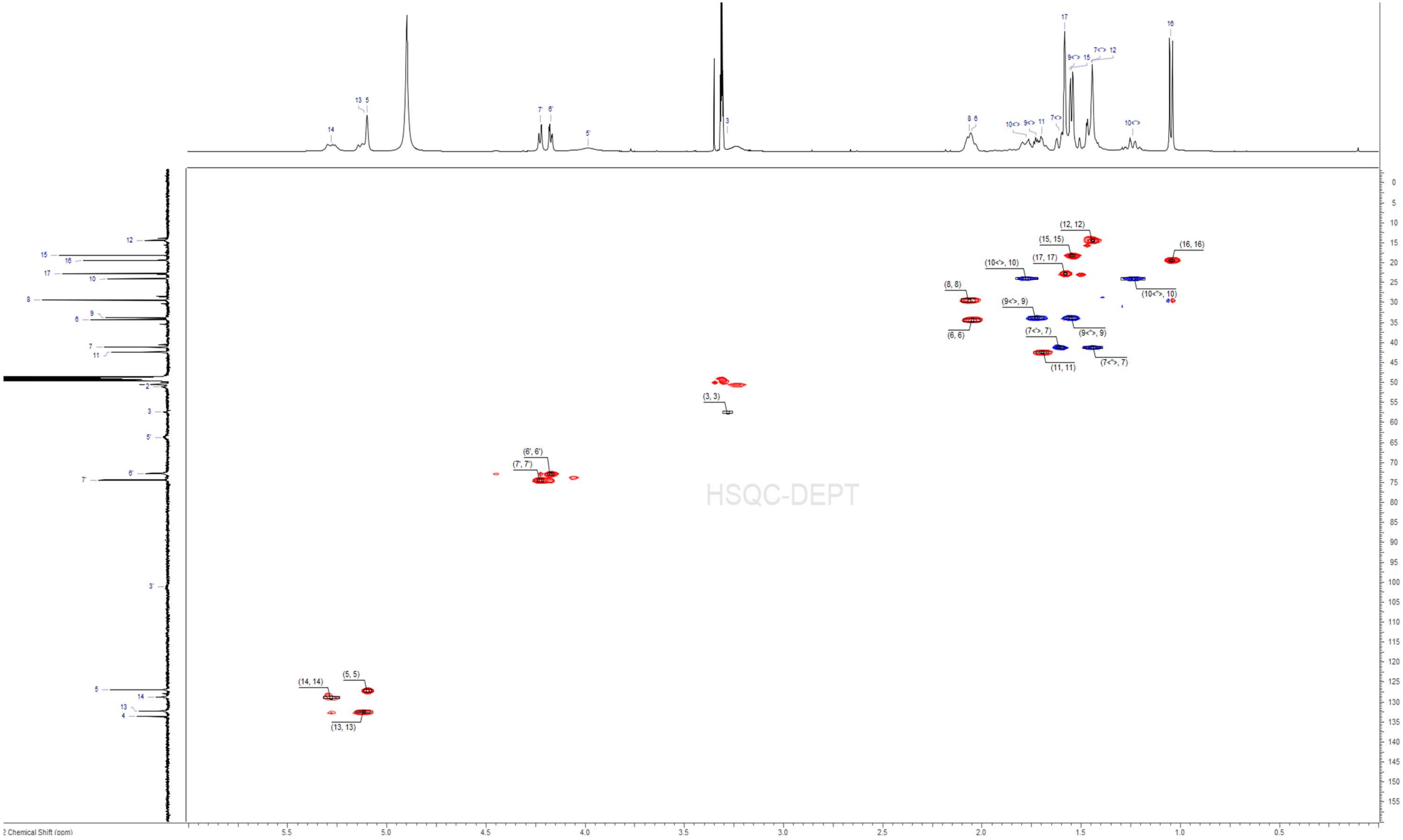

Figure S15. HSQC NMR spectrum of polydosetin B (**2**) (500 MHz, CH_3OH–*d4*).

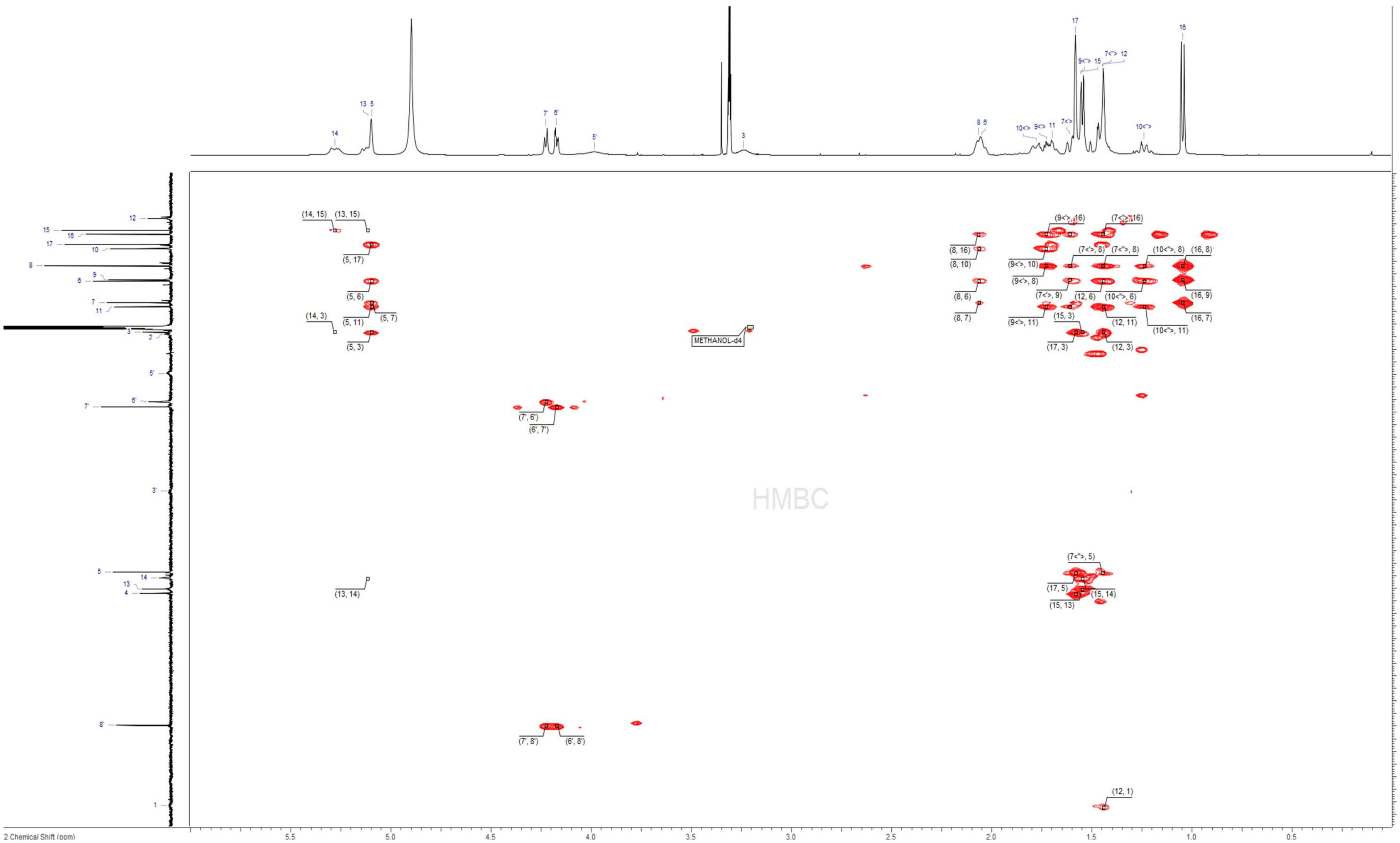

Figure S16. HMBC NMR spectrum of polydosetin B (**2**) (500 MHz, CH_3OH–*d4*).

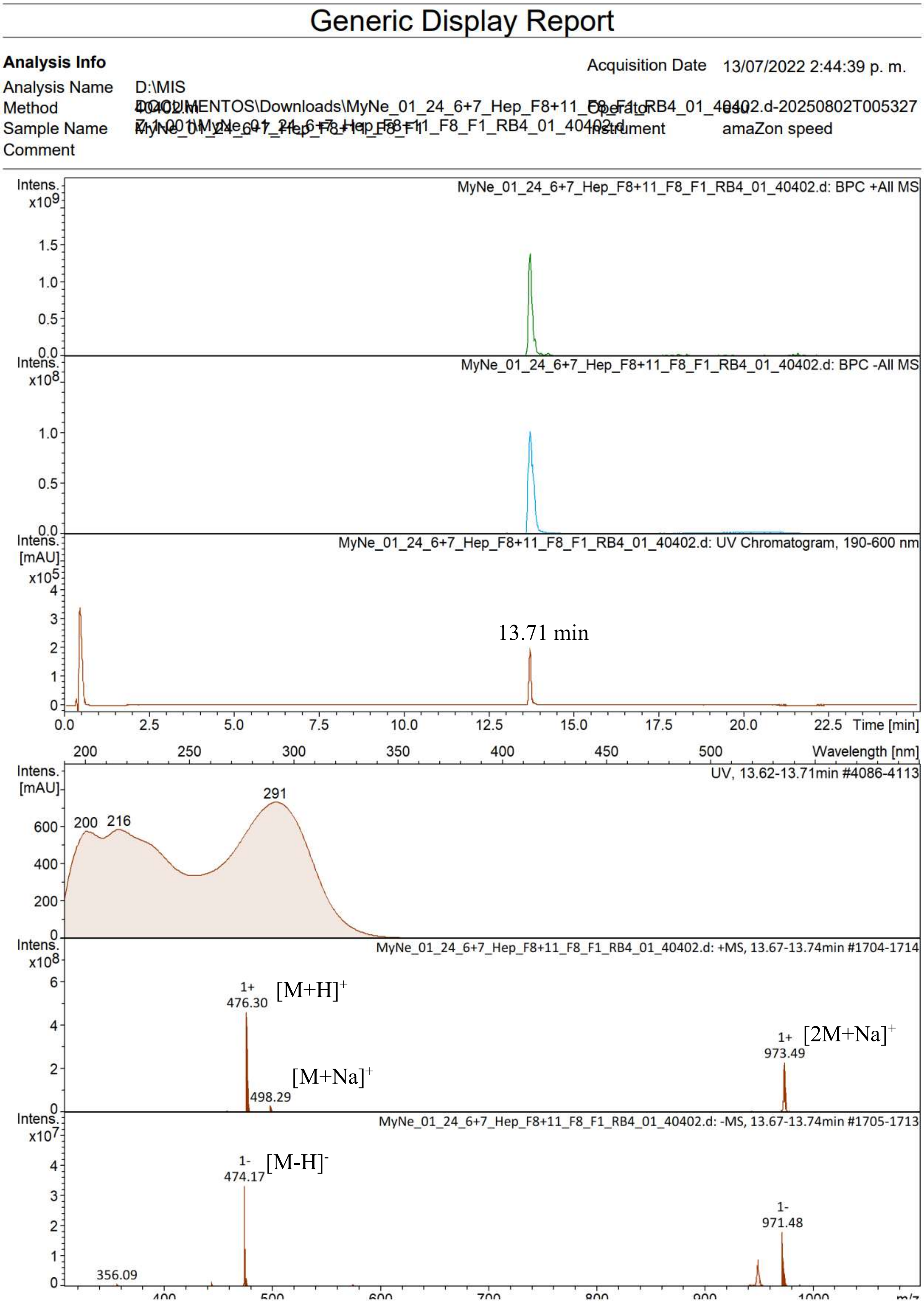

Figure S17. LR-ESI-MS spectrum of polydosetin C (**3**).

Generic Display Report

Analysis Info

Analysis Name	S:\DATA\timsTOF\NLL21_Natalia Llanos\25_03\MyNe_01_24_06+07_Hep_F8+F11_F08_F1_P1-B-6_1_5758.d	Acquisition Date	06-Mar-25 9:18:16 PM
Method	MWIS_BEH50mm_25min_IntThreshold 225.m	Operator	Admin
Sample Name	MyNe_01_24_06+07_Hep_F8+F11_F08_F1	Instrument	timsTOF Pro 2
Comment			

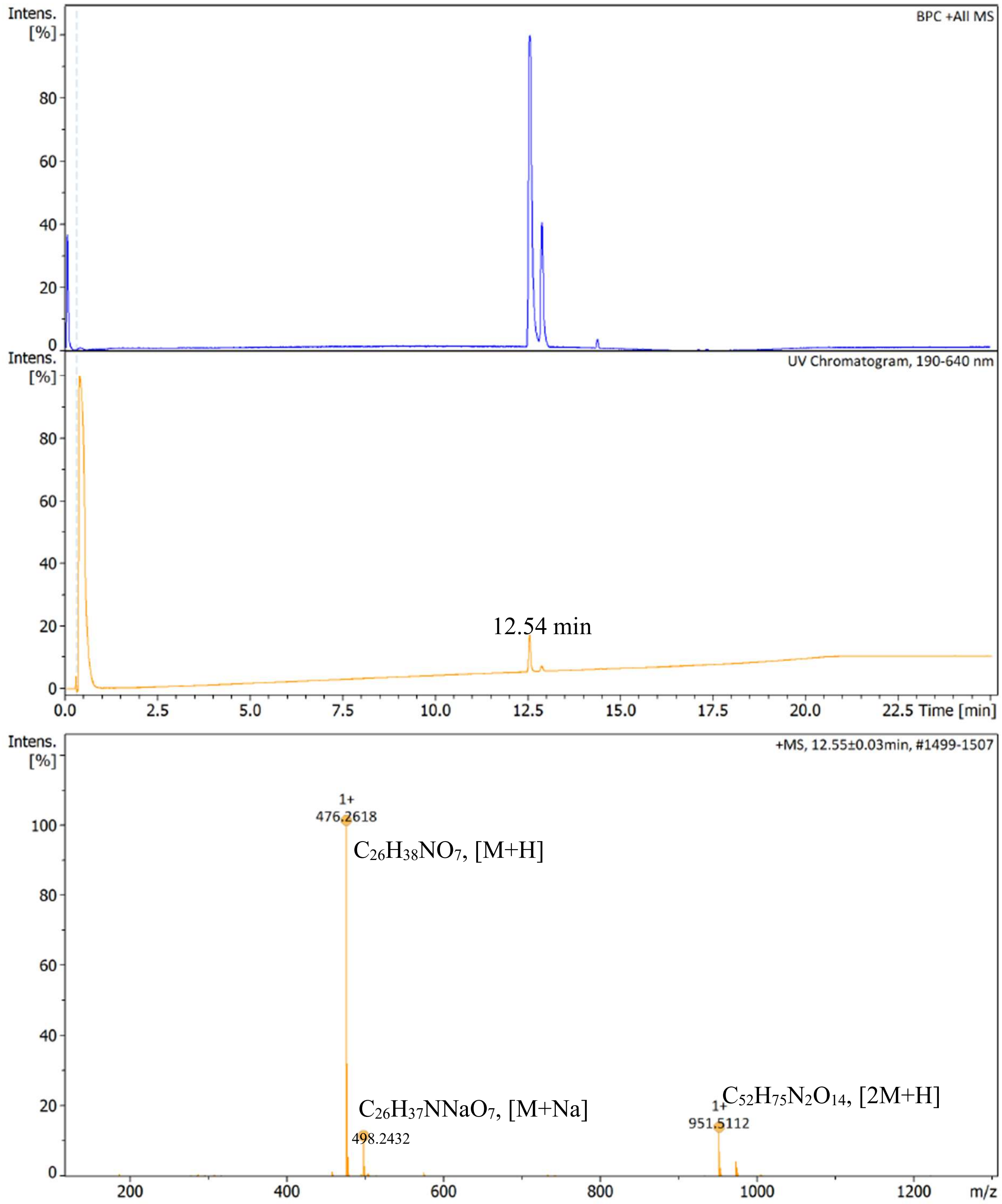

Bruker Compass DataAnalysis 6.1 printed: 01-Aug-25 3:47:32 PM by: nll21 Page 1 of 1

Figure S18. HR-ESI-MS spectrum of polydosetin C (**3**).

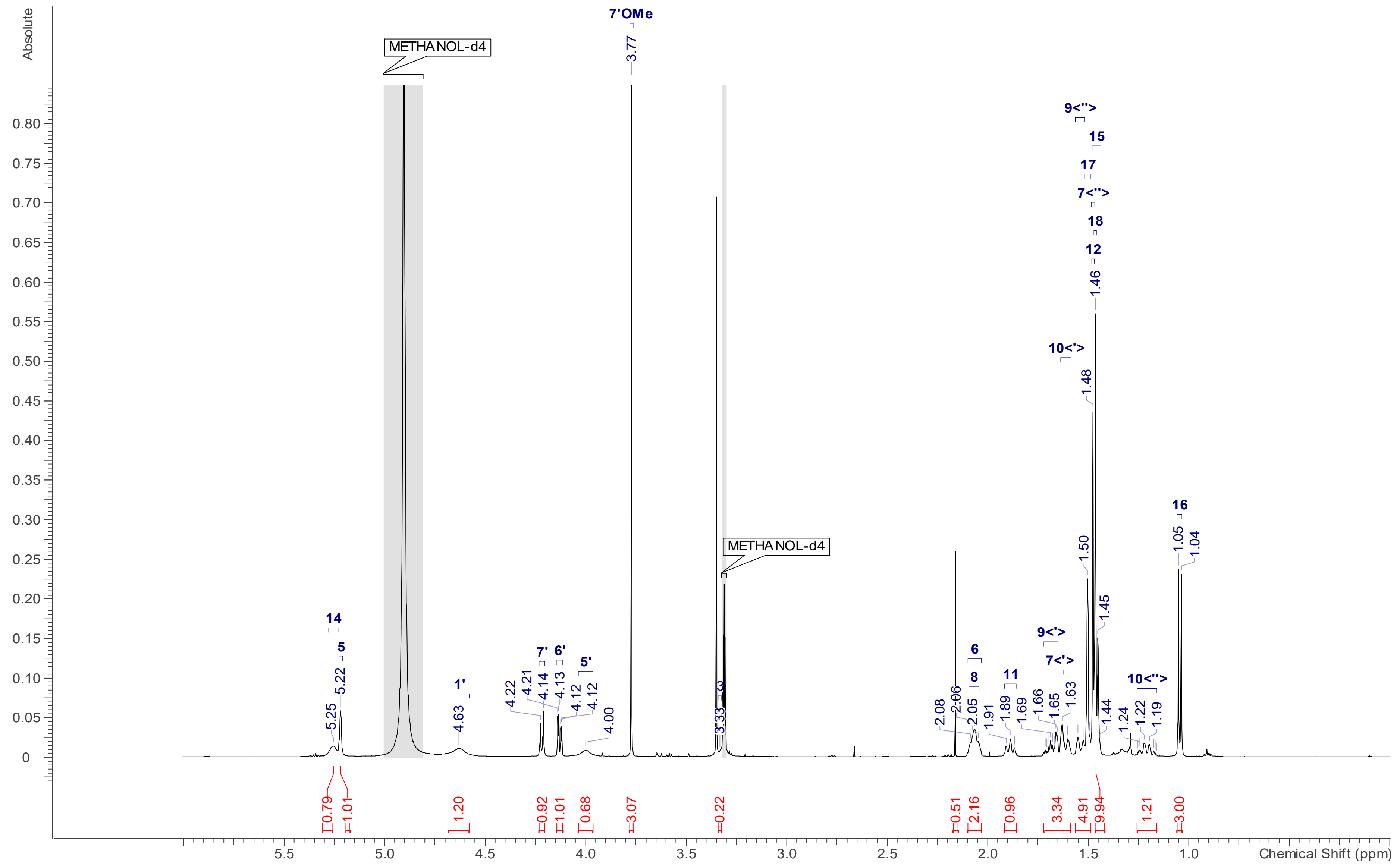

Figure S19. ^{1}H NMR spectrum of polydosetin C (**3**) (500 MHz, CH_3OH–*d4*).

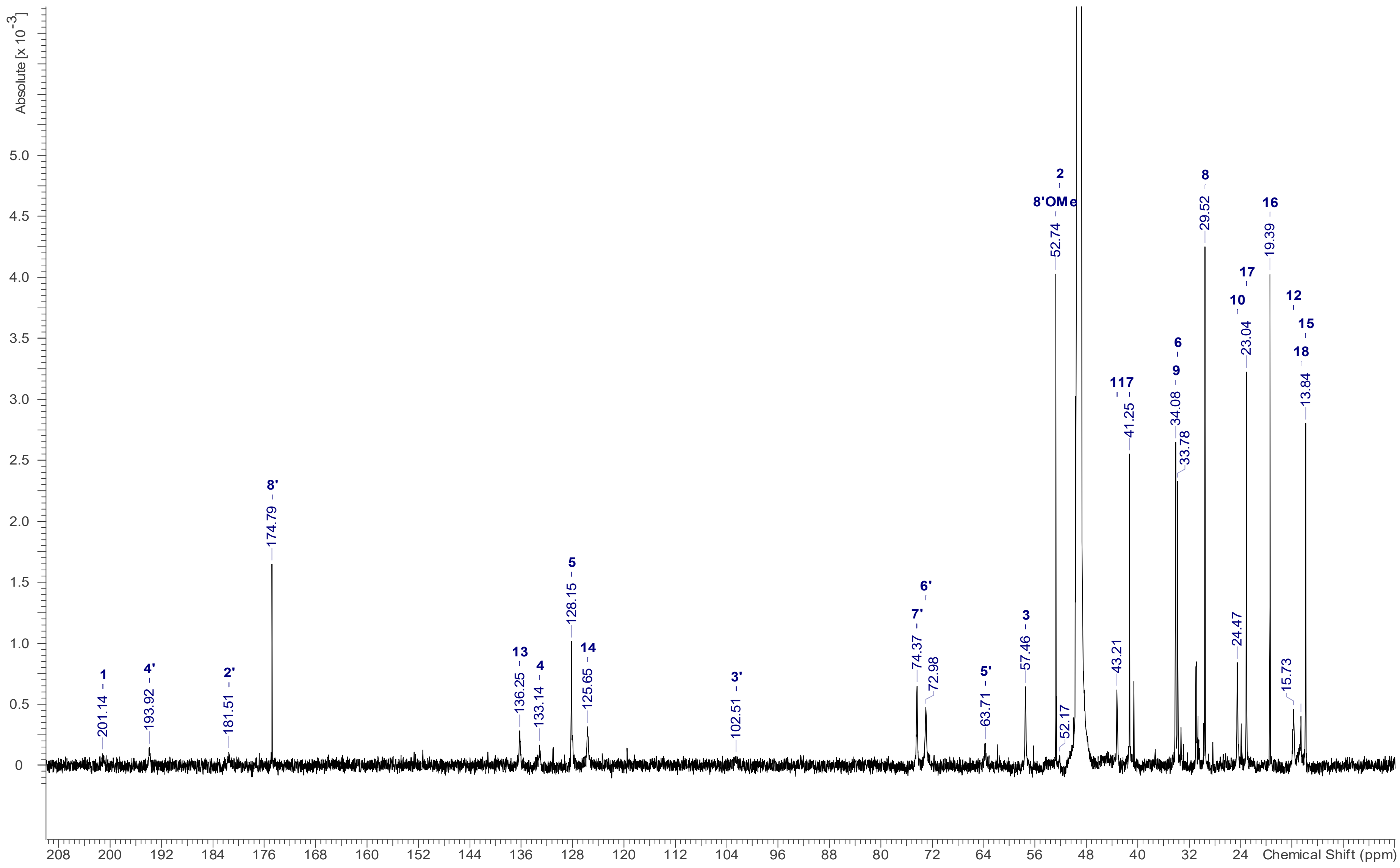

Figure S20. ^{13}C NMR spectrum of polydosetin C (**3**) (175 MHz, CH_3OH–*d4*).

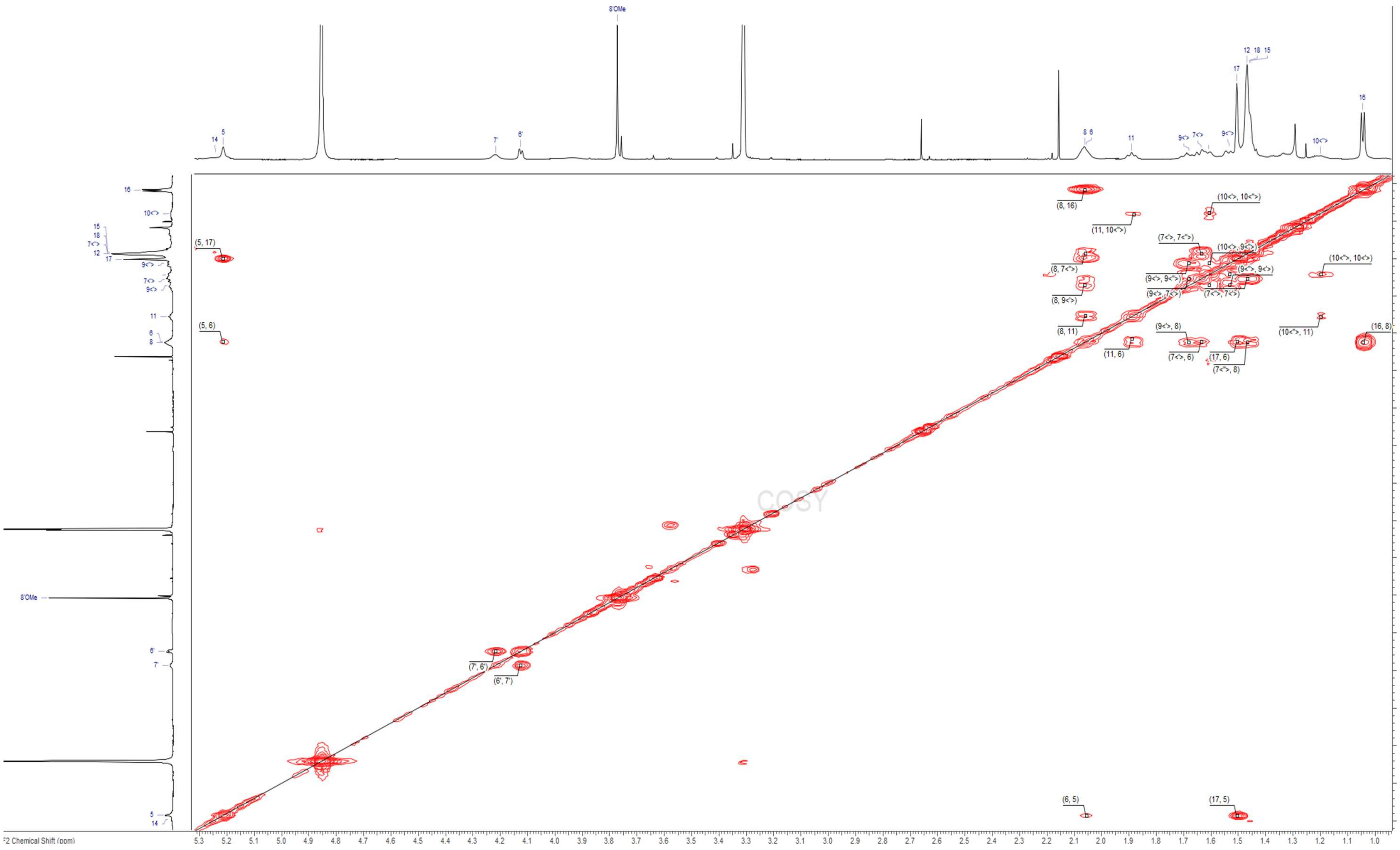

Figure S21. COSY NMR spectrum of polydosetin C (**3**) (700 MHz, CH_3OH–*d4*).

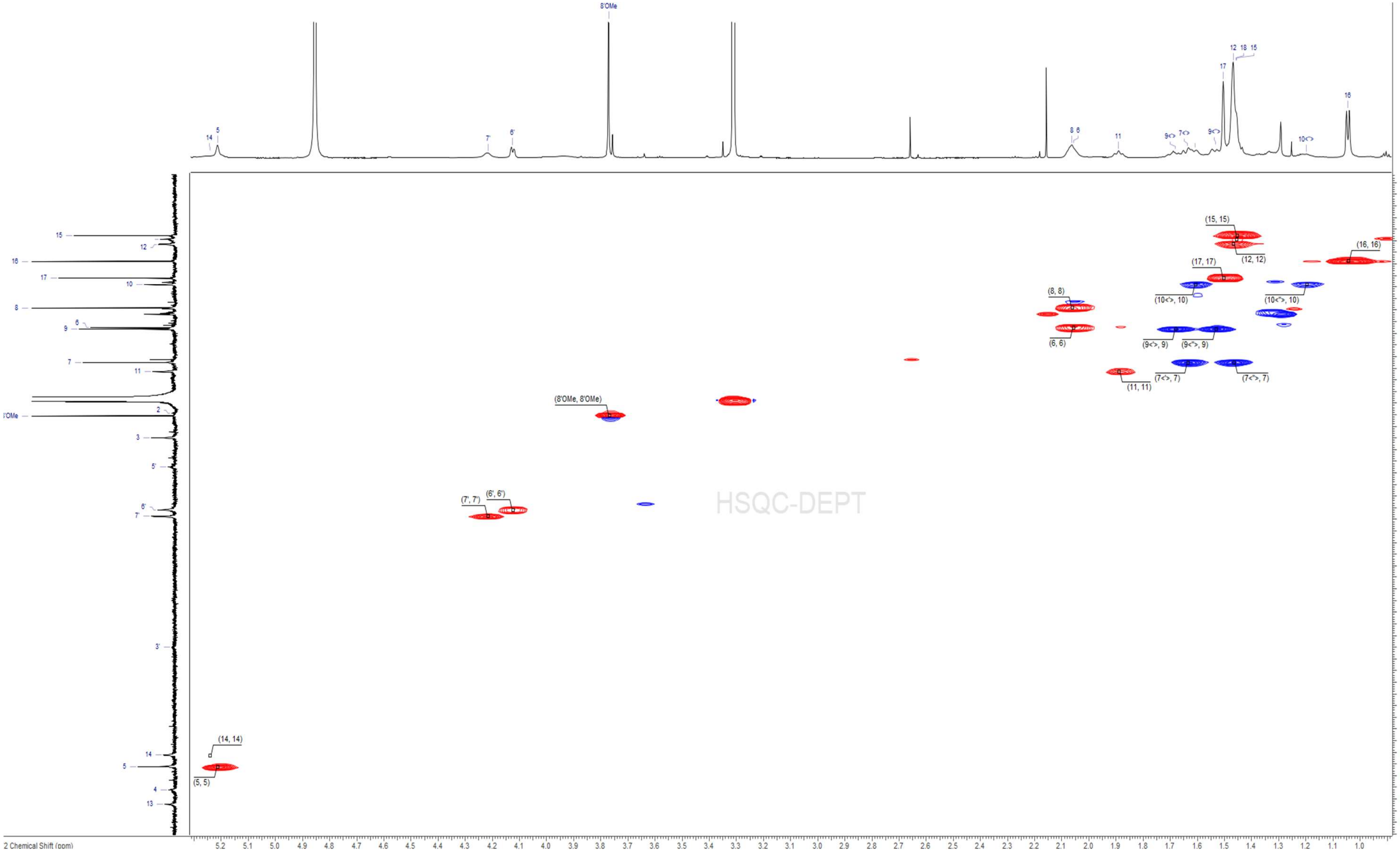

Figure S22. HSQC NMR spectrum of polydosetin C (**3**) (700 MHz, CH_3OH–*d4*).

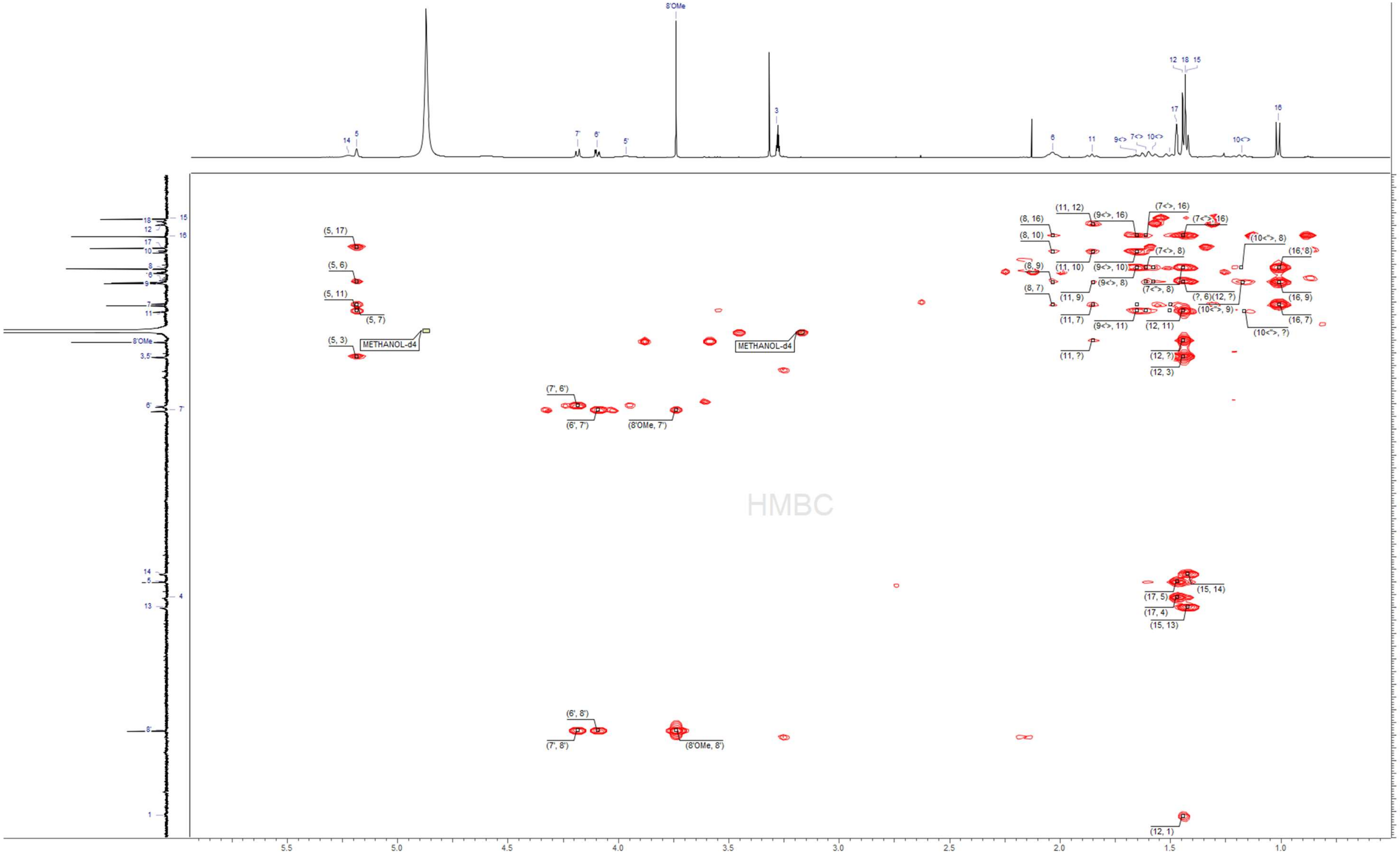

Figure S23. HMBC NMR spectrum of polydosetin C (**3**) (500 MHz, CH_3OH–*d4*).

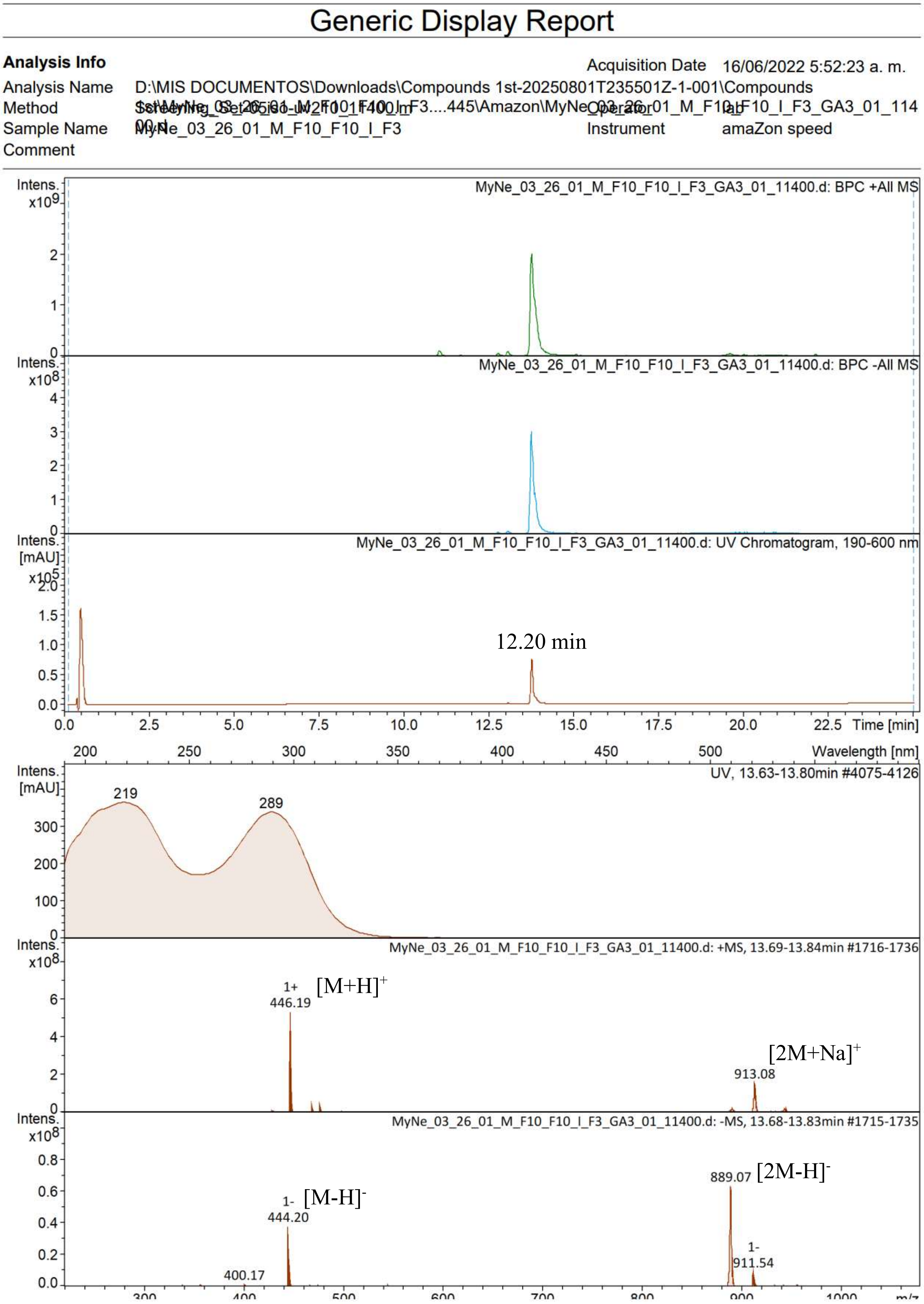

Figure S24. LR-ESI-MS spectrum of polydosetin D (**4**).

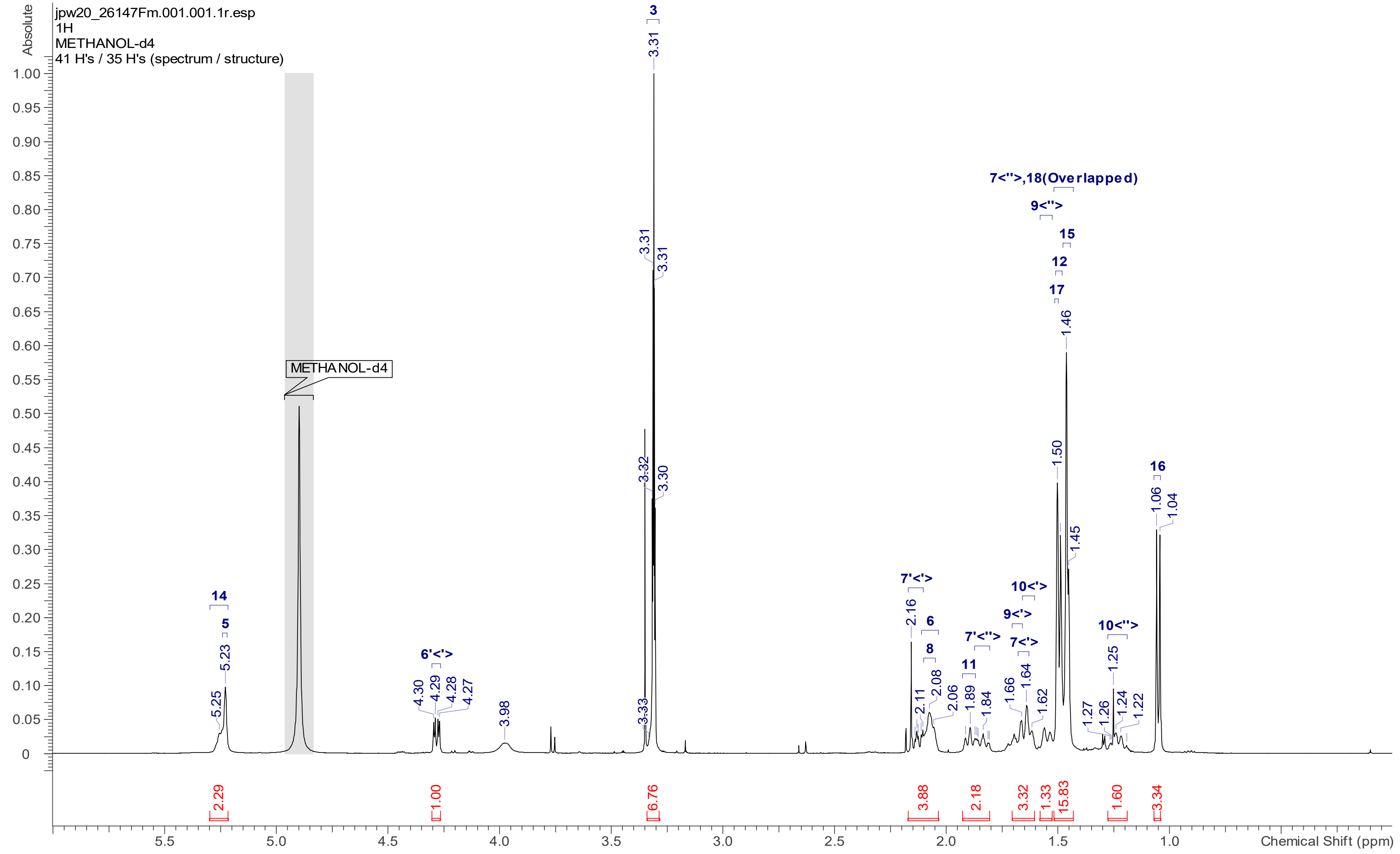

Figure S25. ^{1}H NMR spectrum of polydosetin D (**4**) (500 MHz, CH_3OH–*d4*).

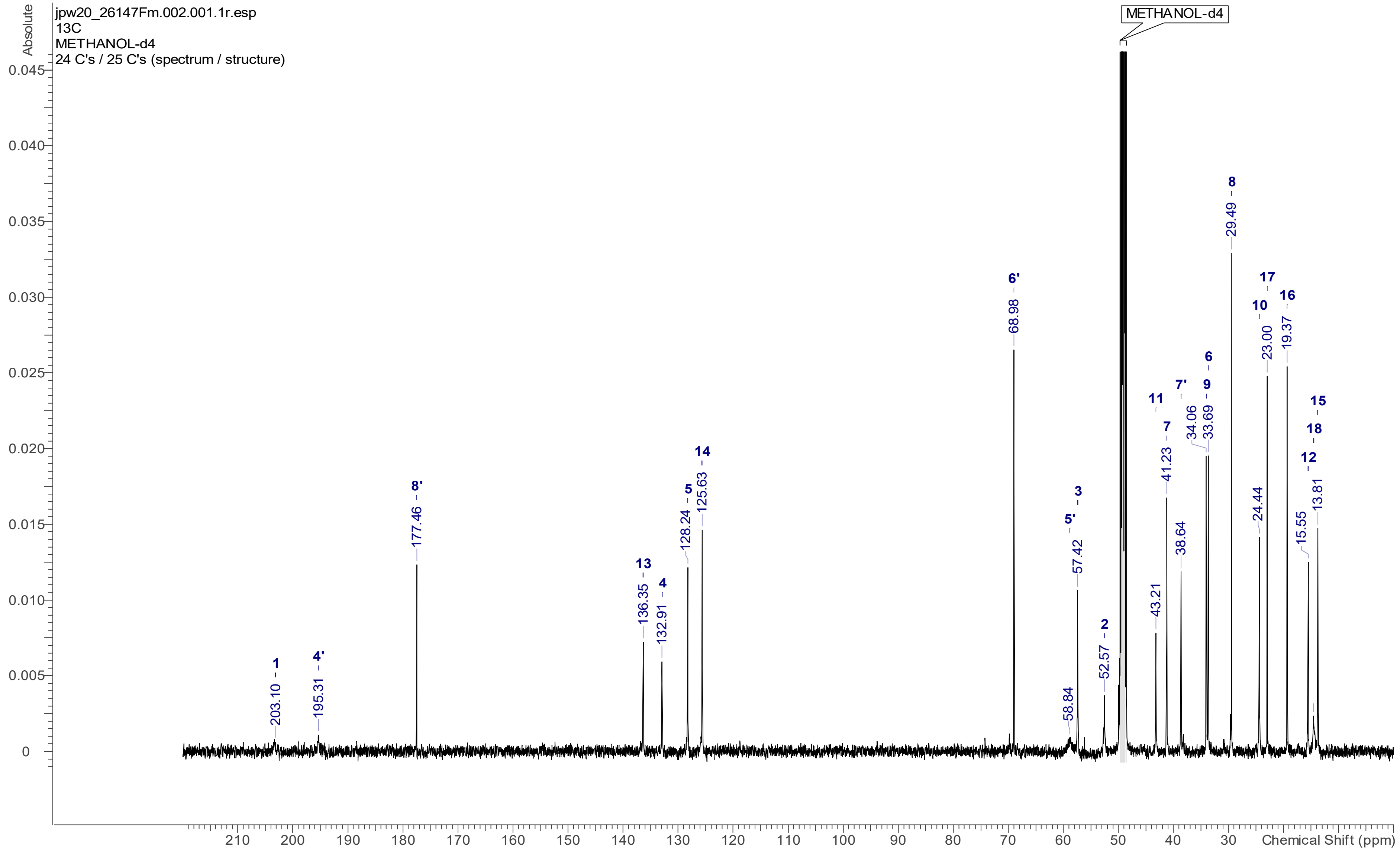

Figure S26. ^{13}C NMR spectrum of polydosetin D (**4**) (125 MHz, CH_3OH–*d4*).

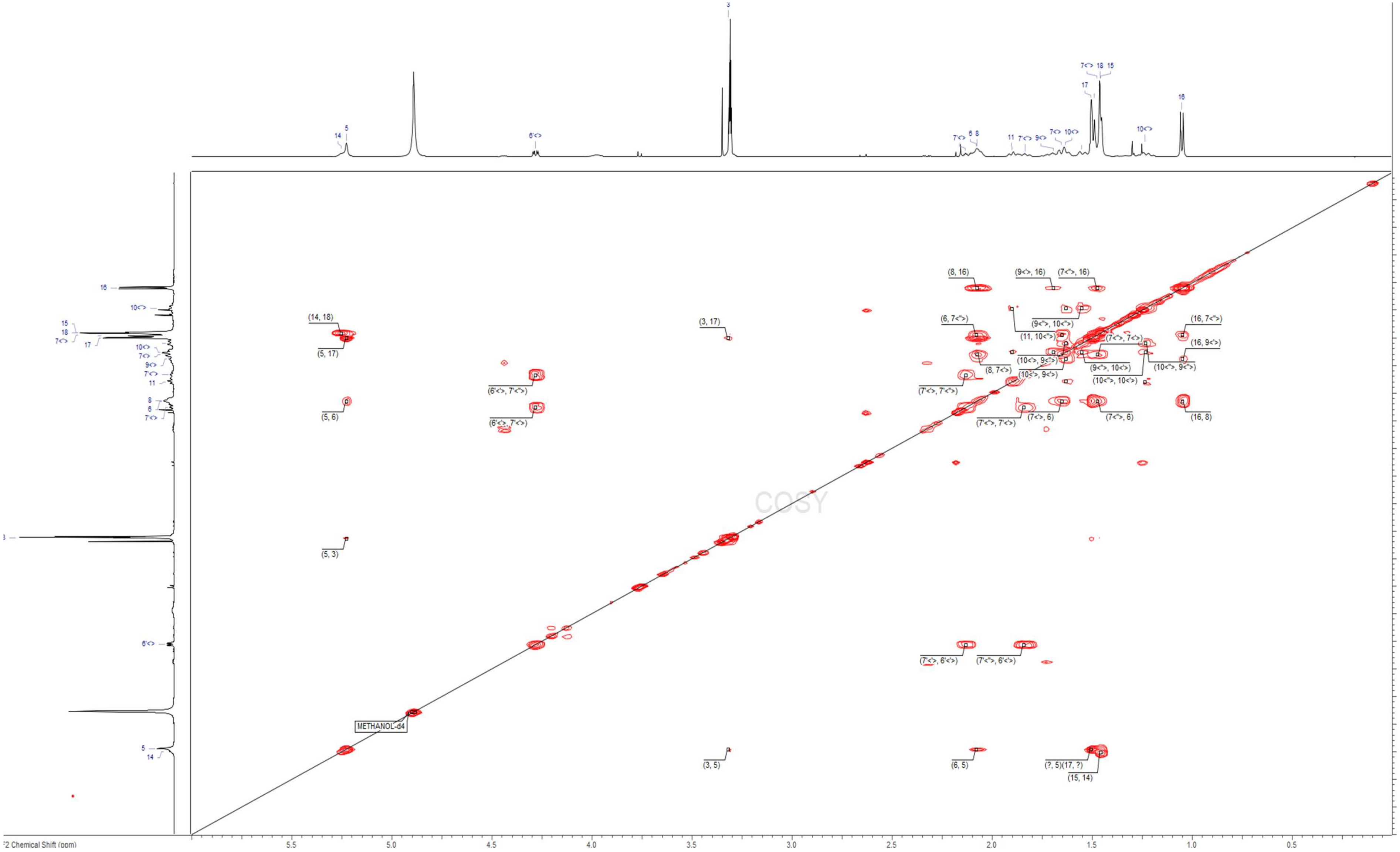

Figure S27. COSY NMR spectrum of polydosetin D (**4**) (500 MHz, CH_3OH–*d4*).

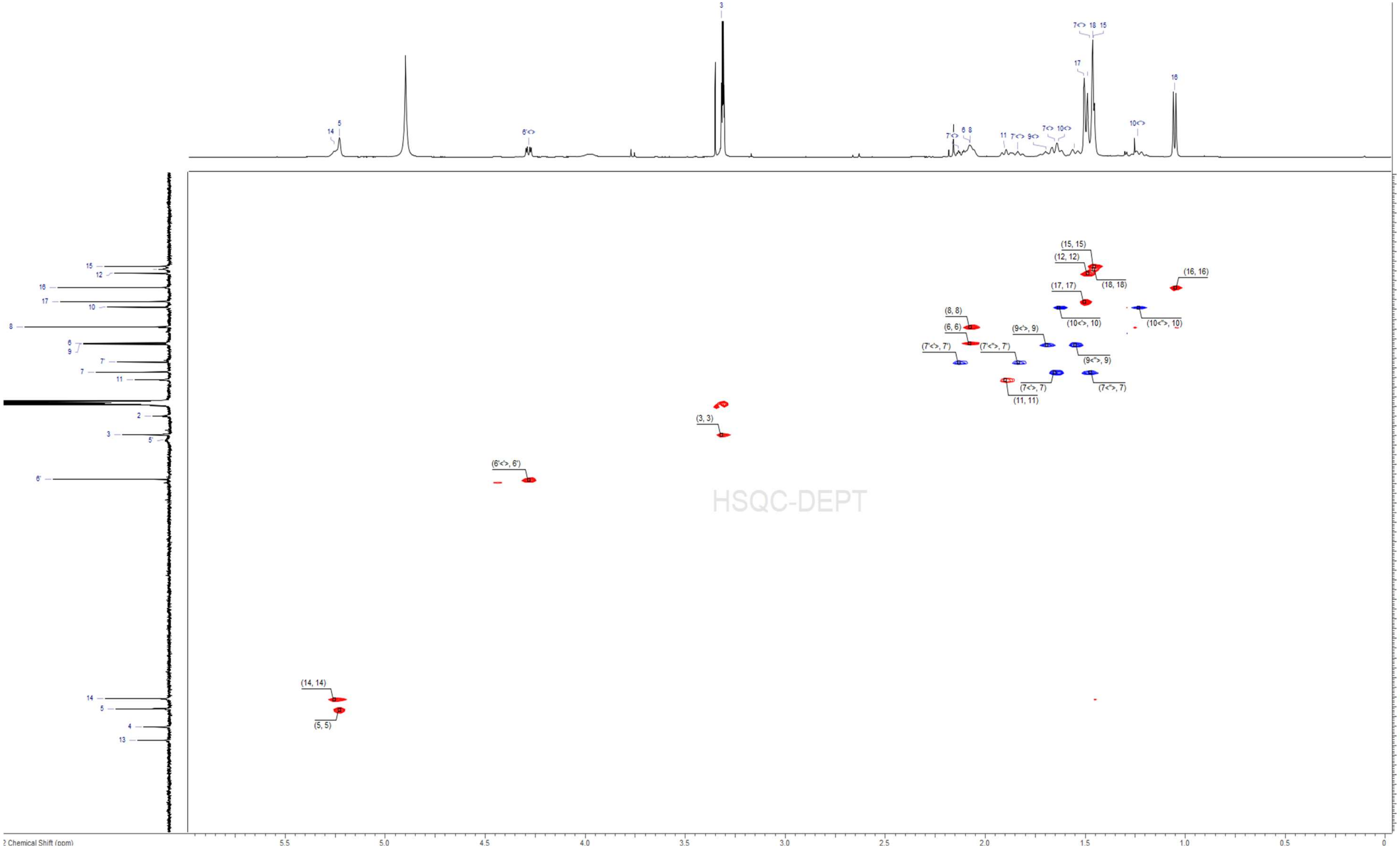

Figure S28. HSQC NMR spectrum of polydosetin D (**4**) (500 MHz, CH_3OH–*d4*).

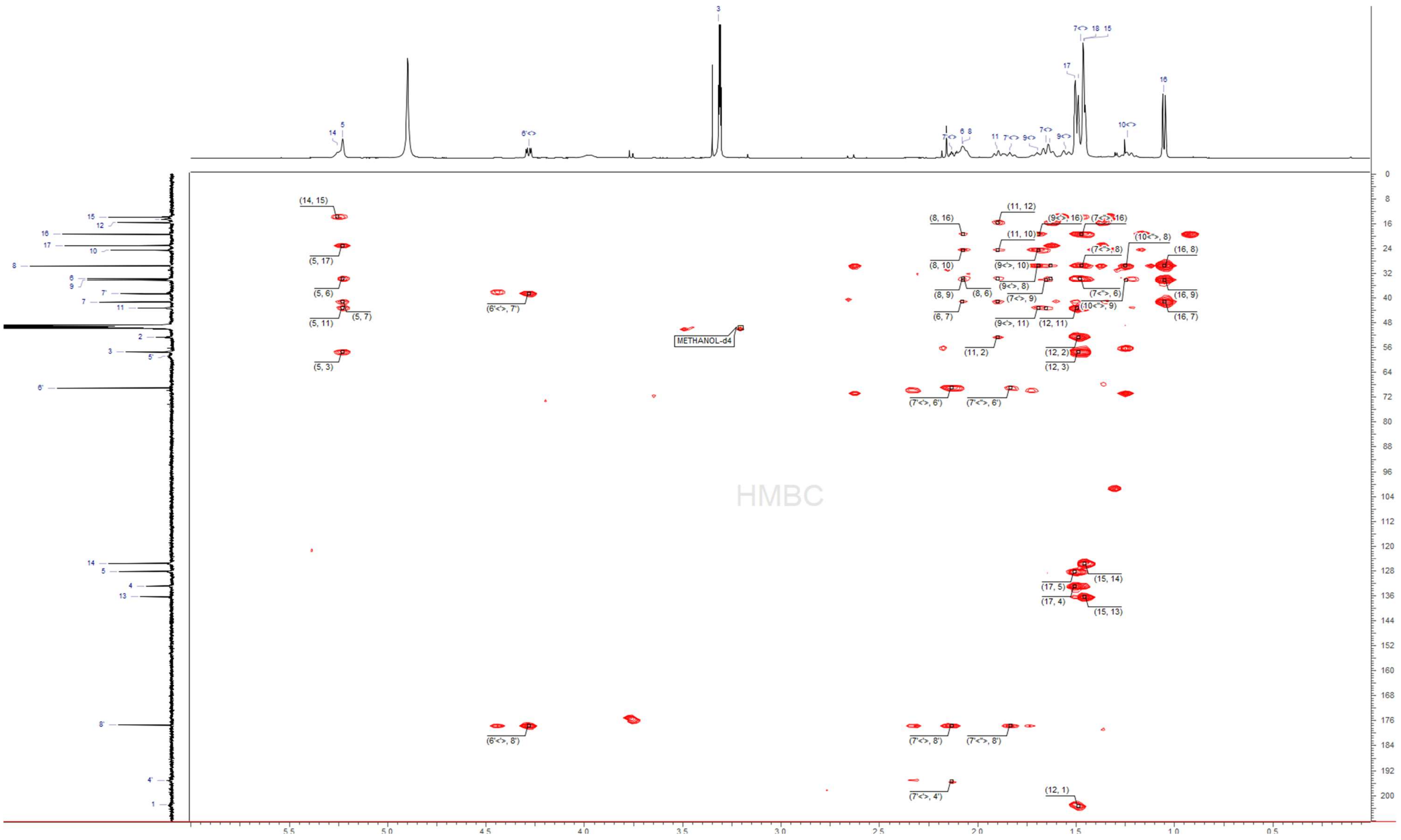

Figure S29. HMBC NMR spectrum of polydosetin D (**4**) (500 MHz, CH_3OH–*d4*).

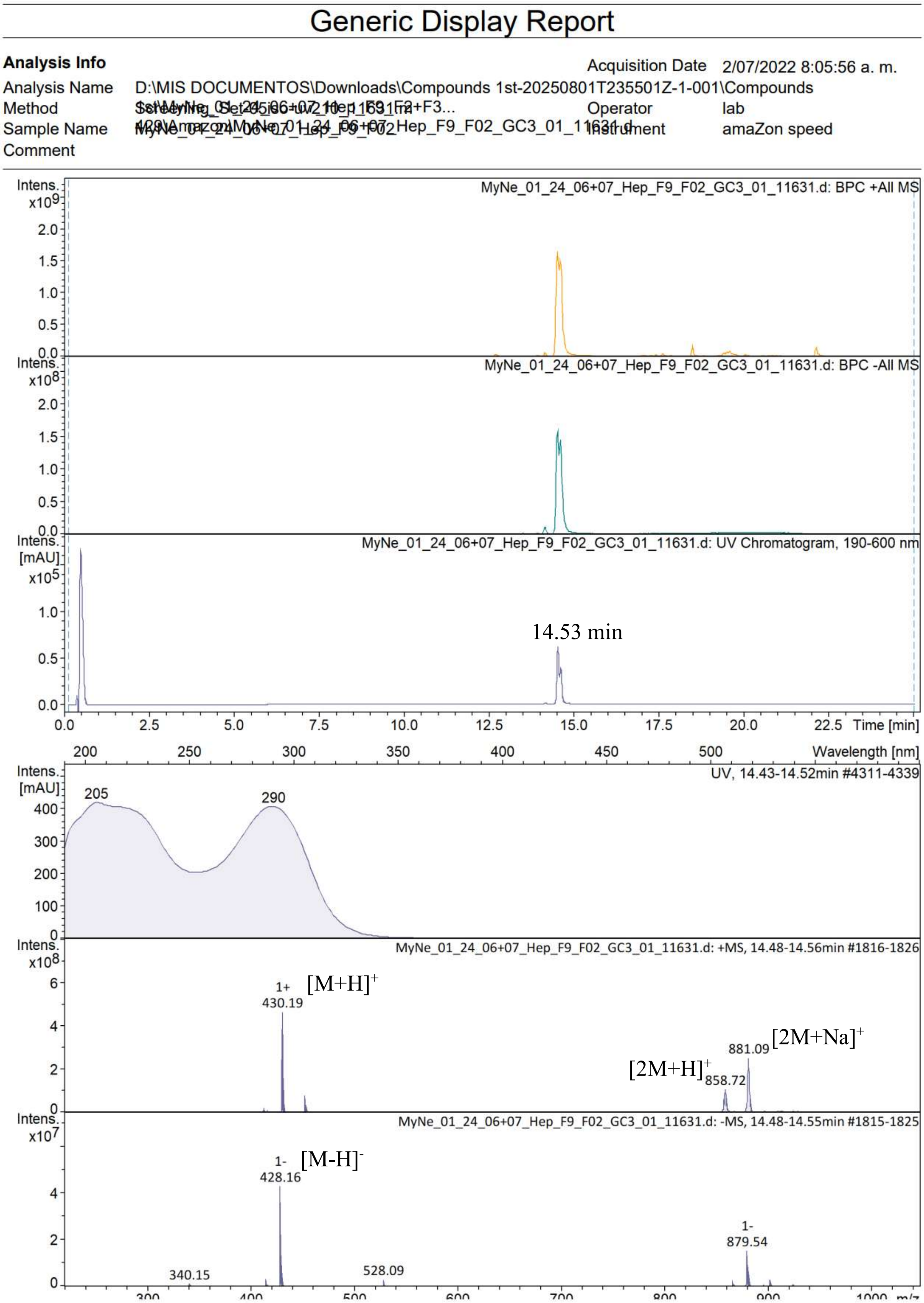

Figure S30. LR-ESI-MS spectrum of polydosetin E (**5**).

Generic Display Report

Analysis Info

Analysis Name	S:\DATA\timsTOF\NLL21_Natalia Llanos\25_03\MyNe_01_24_06+07_Hep_F9_F2.d	Acquisition Date	12-Dec-24 3:02:21 AM
Method	MWIS_BEH50mm_25min_IntThreshold 225.m	Operator	Admin
Sample Name	MyNe_01_24_06+07_Hep_F8+11_F17_F5	Instrument	timsTOF Pro 2
Comment			

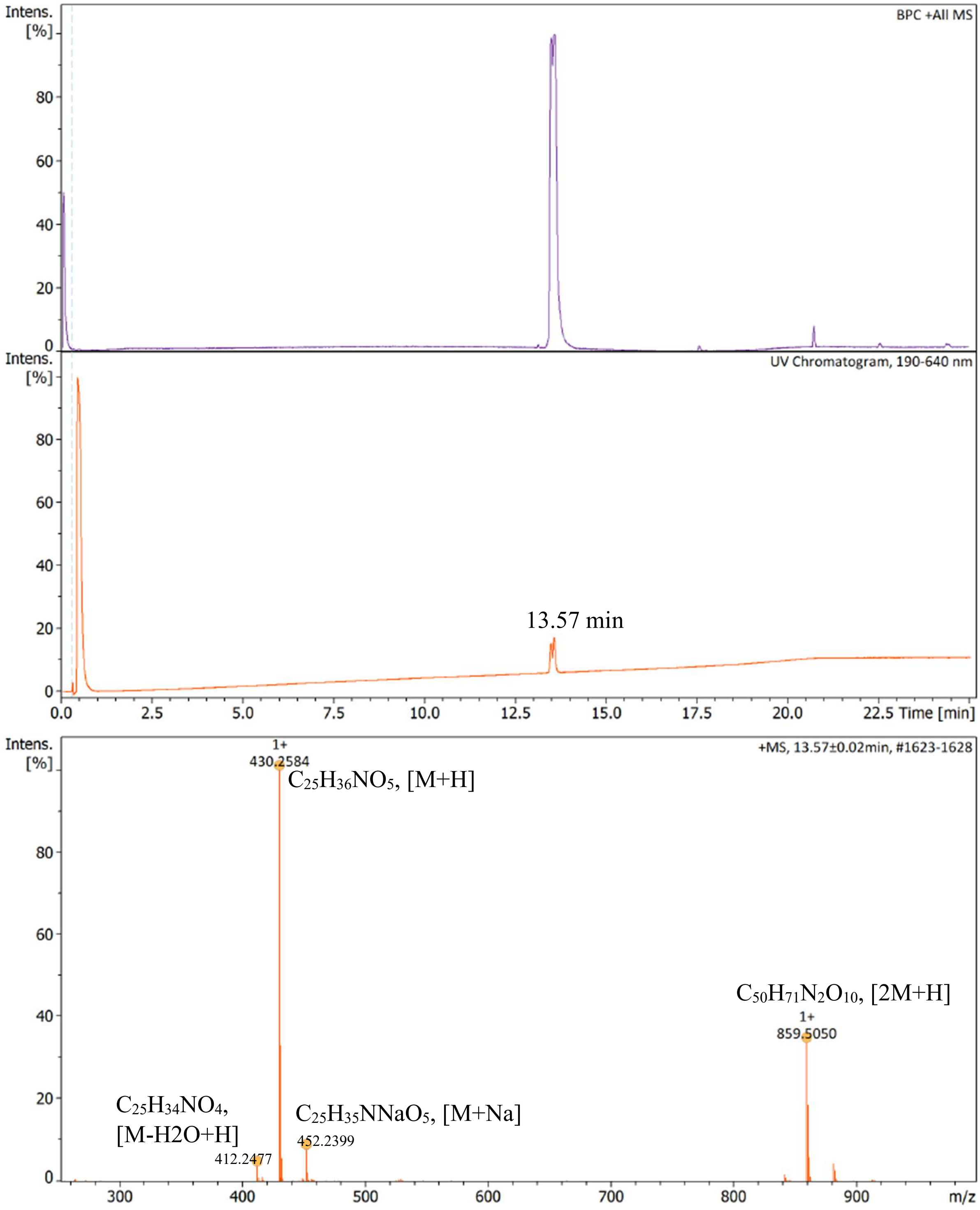

Bruker Compass DataAnalysis 6.1 printed: 01-Aug-25 5:22:45 PM by: nll21 Page 1 of 1

Figure S31. HR-ESI-MS spectrum of polydosetin E (**5**).

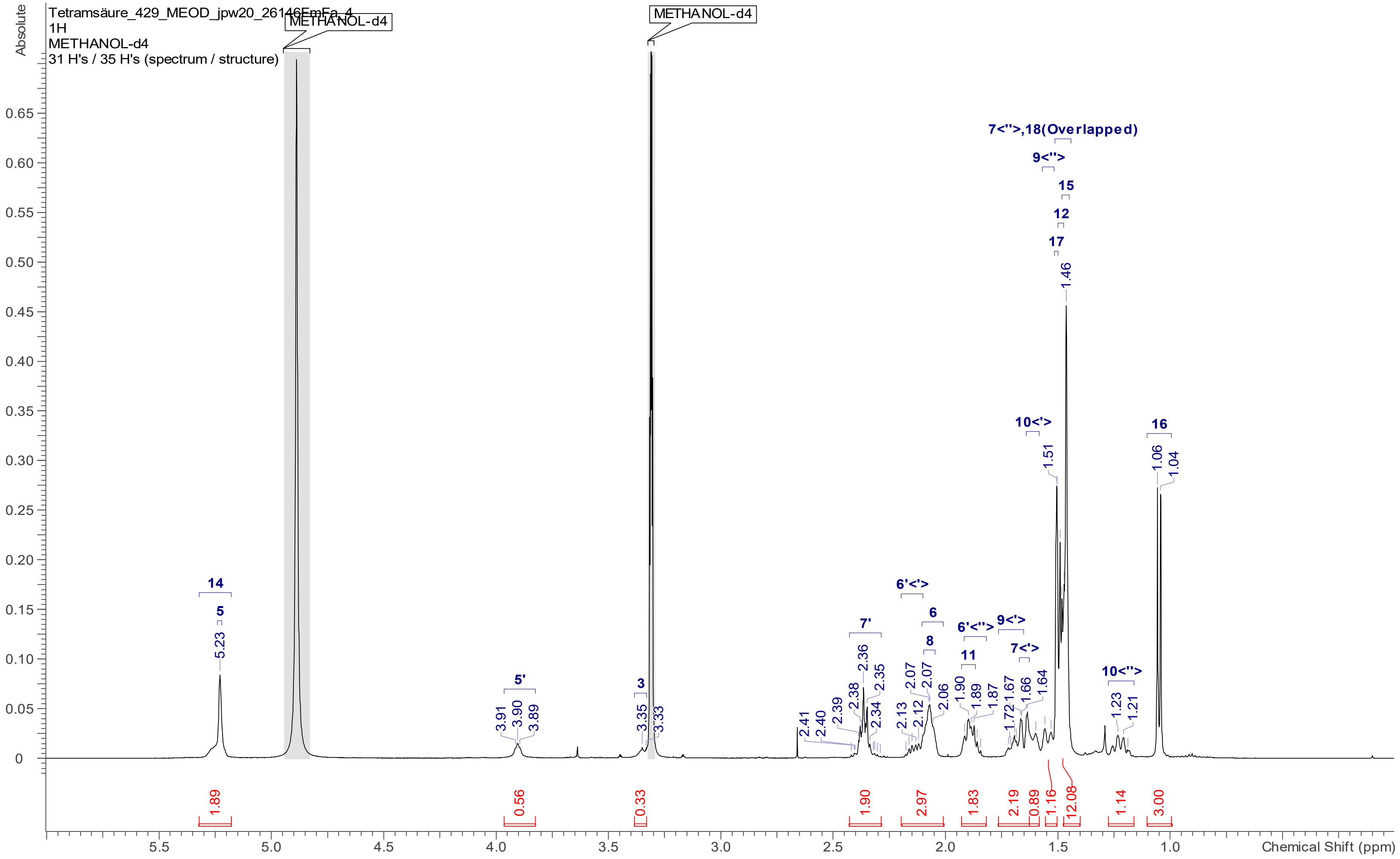

Figure S32. ^{1}H NMR spectrum of polydosetin E (**5**) (500 MHz, CH_3OH–*d4*).

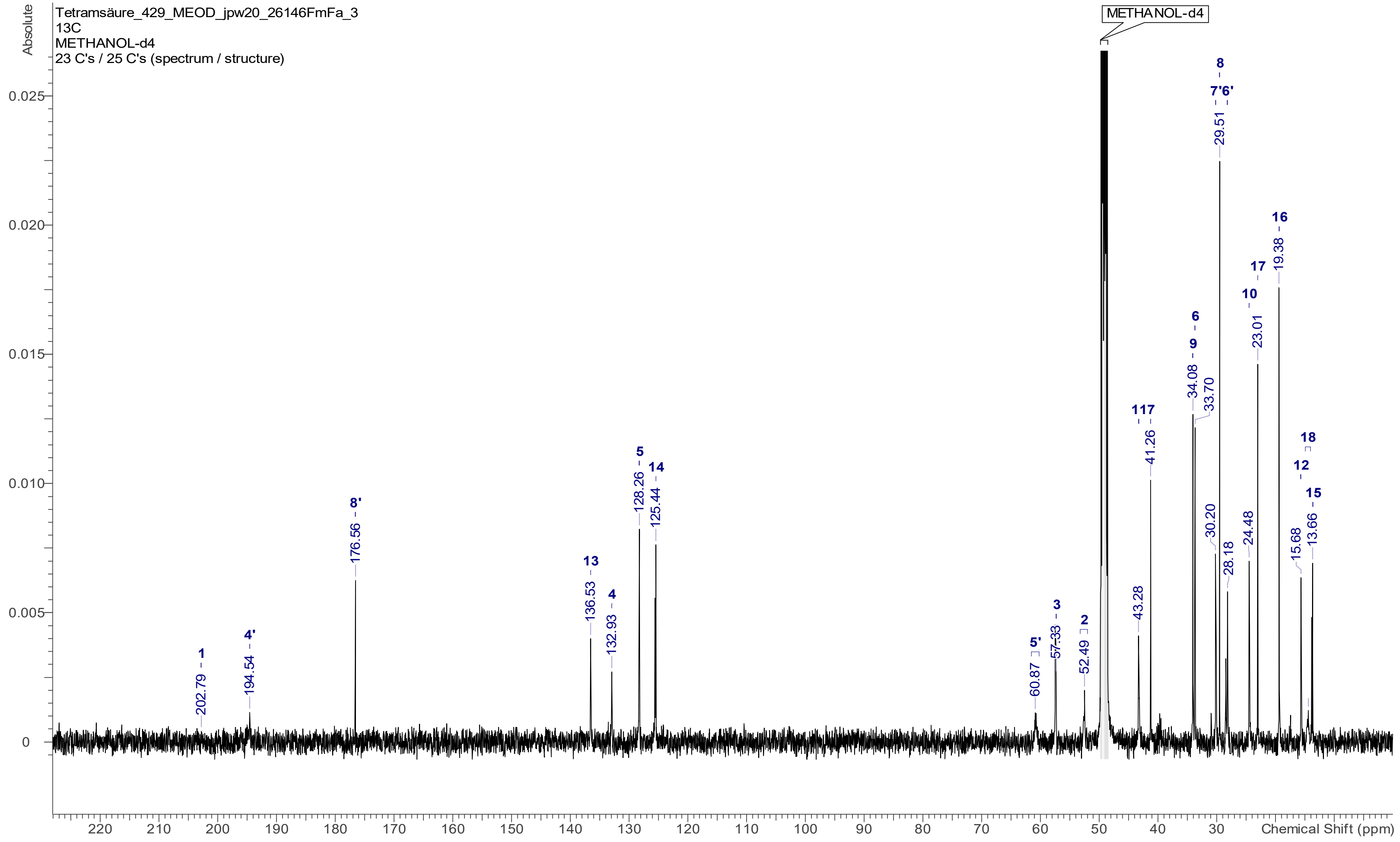

Figure S33. ^{13}C NMR spectrum of polydosetin E (**5**) (125 MHz, CH_3OH–*d4*).

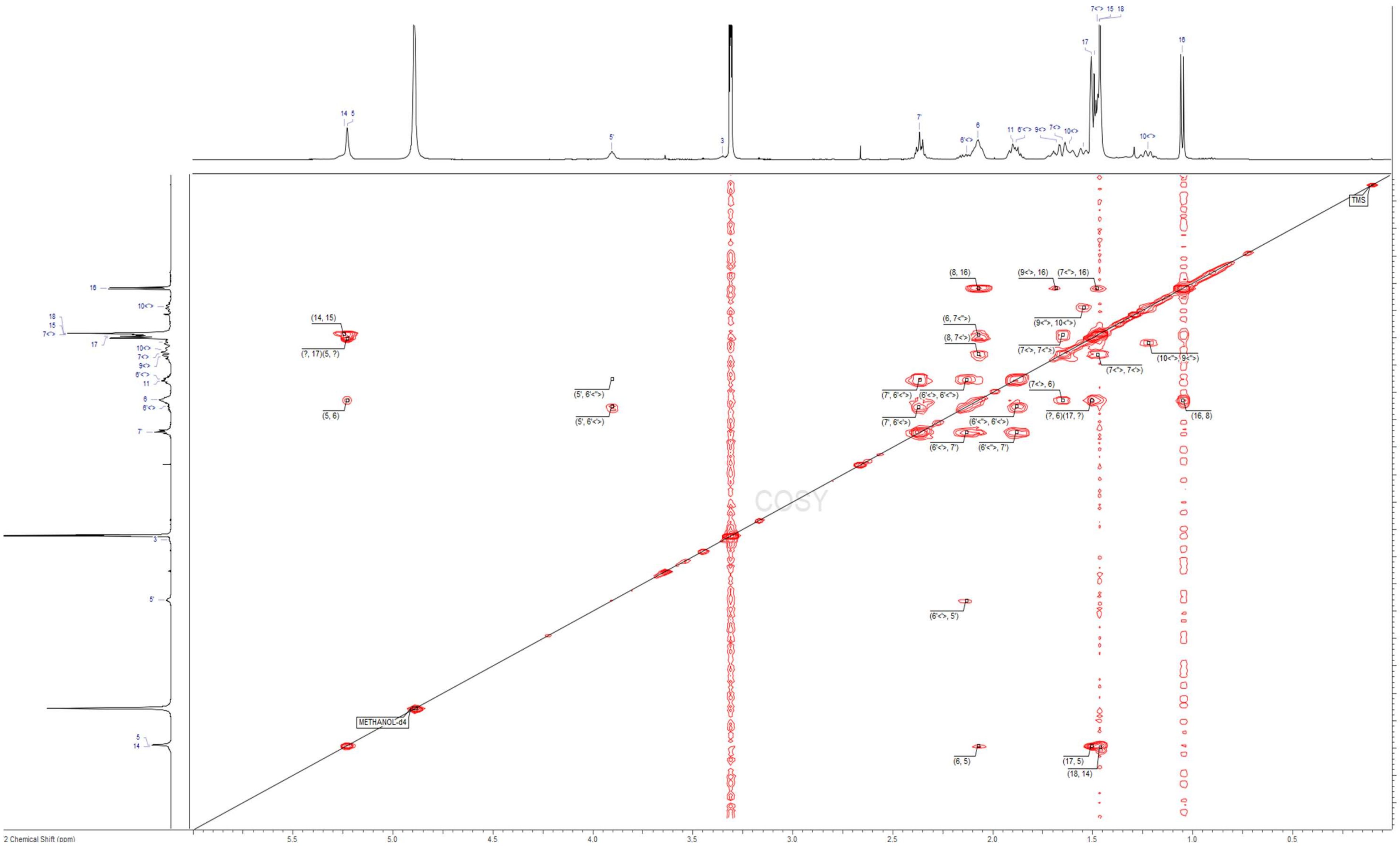

Figure S34. COSY NMR spectrum of polydosetin E (**5**) (500 MHz, CH_3OH–*d4*).

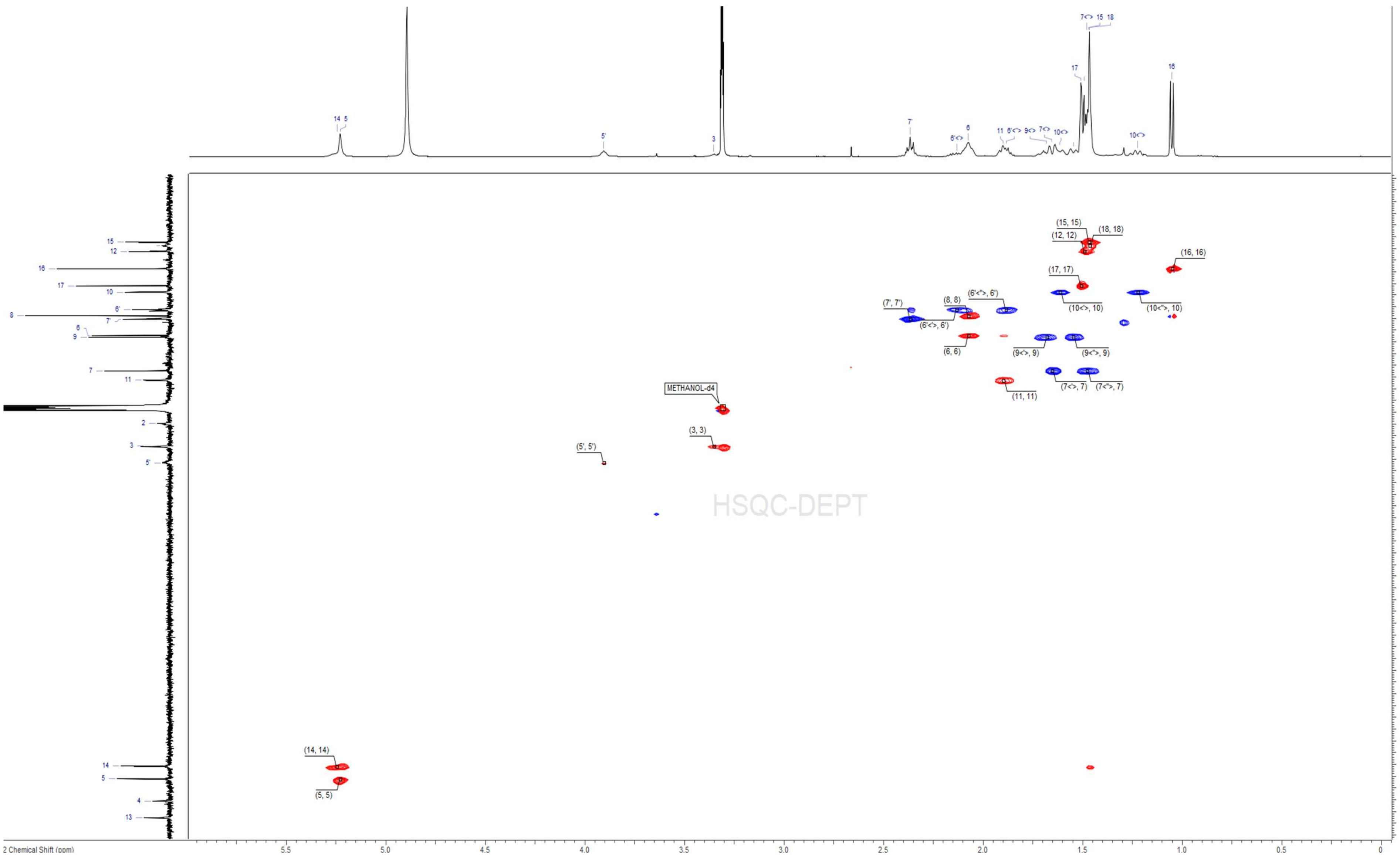

Figure S35. HSQC NMR spectrum of polydosetin E (**5**) (500 MHz, CH_3OH–*d4*).

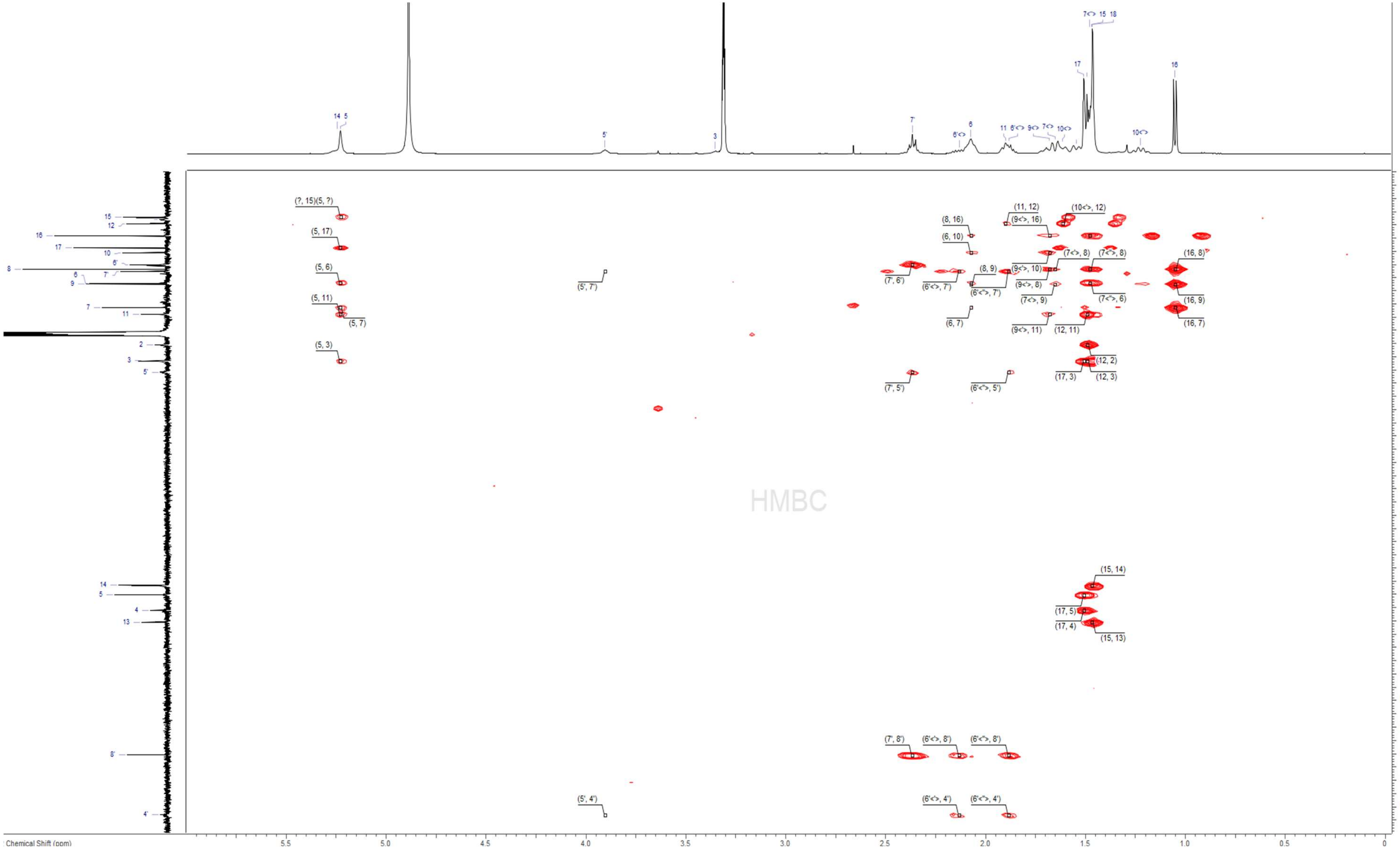

Figure S36. HMBC NMR spectrum of polydosetin E (**5**) (500 MHz, CH_3OH–*d4*).

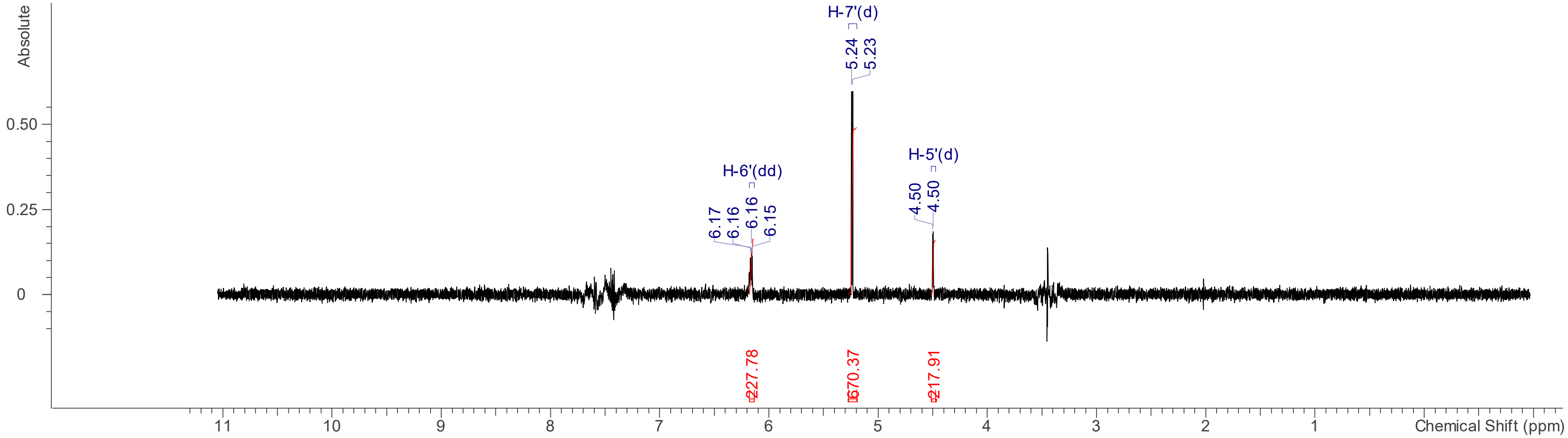

Figure S37. 1D TOCSY NMR spectrum (700 MHz, pyridine–d_5) of the **3**-(*S*)-MTPA bisester with irradiation on H–6’ at δ_H 6.16 ppm.

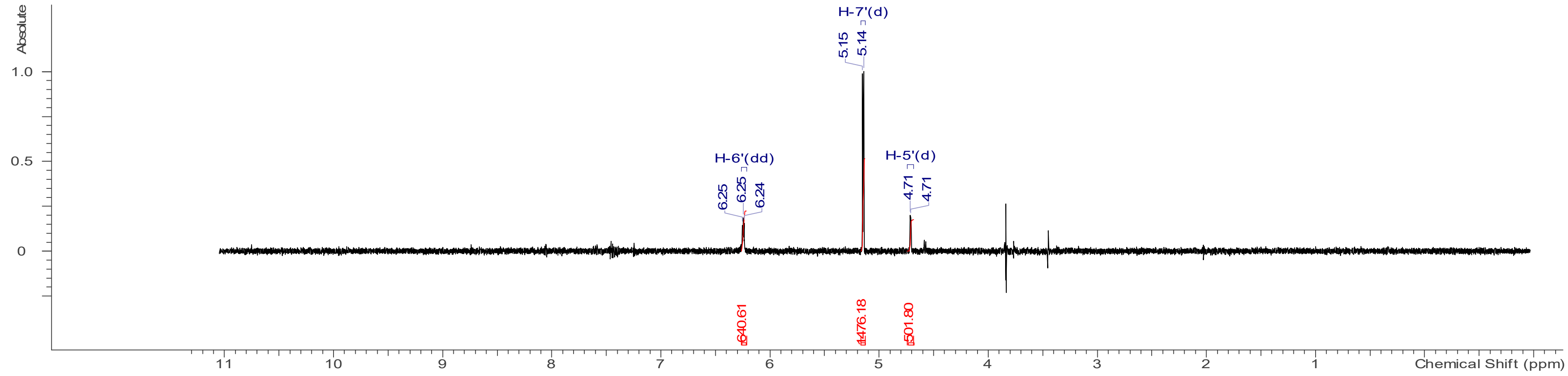

Figure S38. 1D TOCSY NMR spectrum (700 MHz, pyridine–*d5*) of the **3**-(*R*)-MTPA bisester with irradiation on H–6’ at δ_H 6.24 ppm.

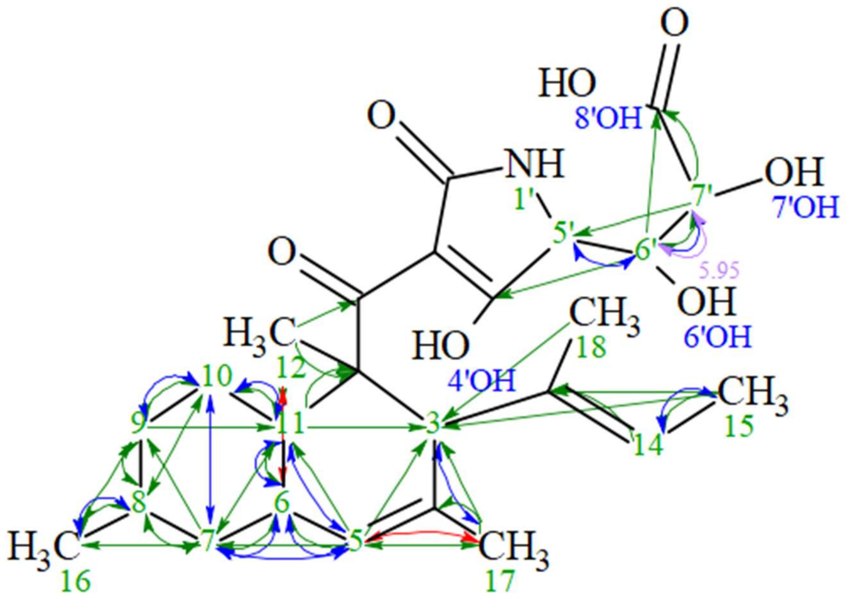

Figure S39. COSY (blue arrows) and HMBC (green arrows) correlations utilized in the structure elucidation process of **1**

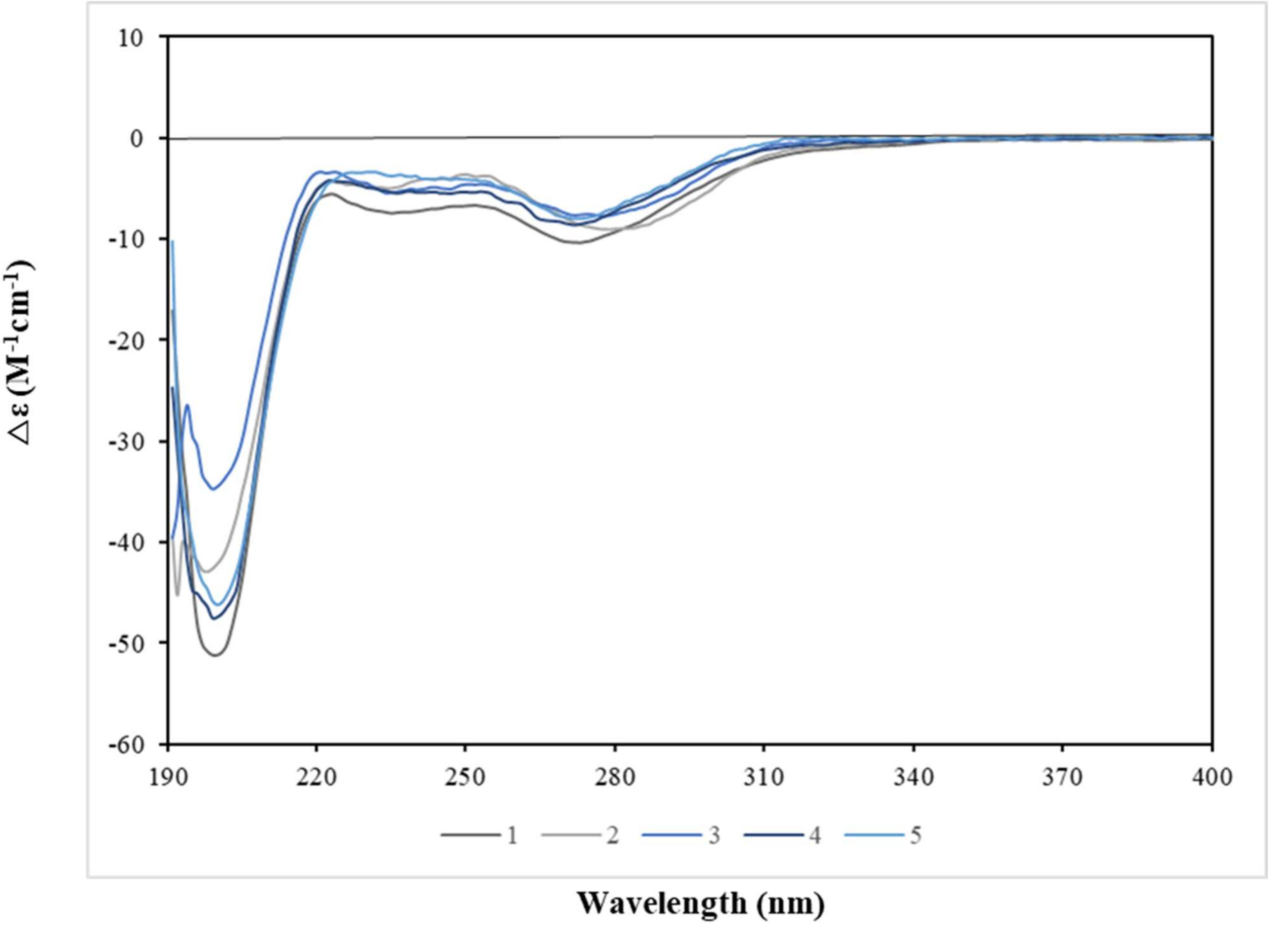

Figure S40. Experimental ECD spectrum of polydosetins A–E (**1**–**5**) in methanol.

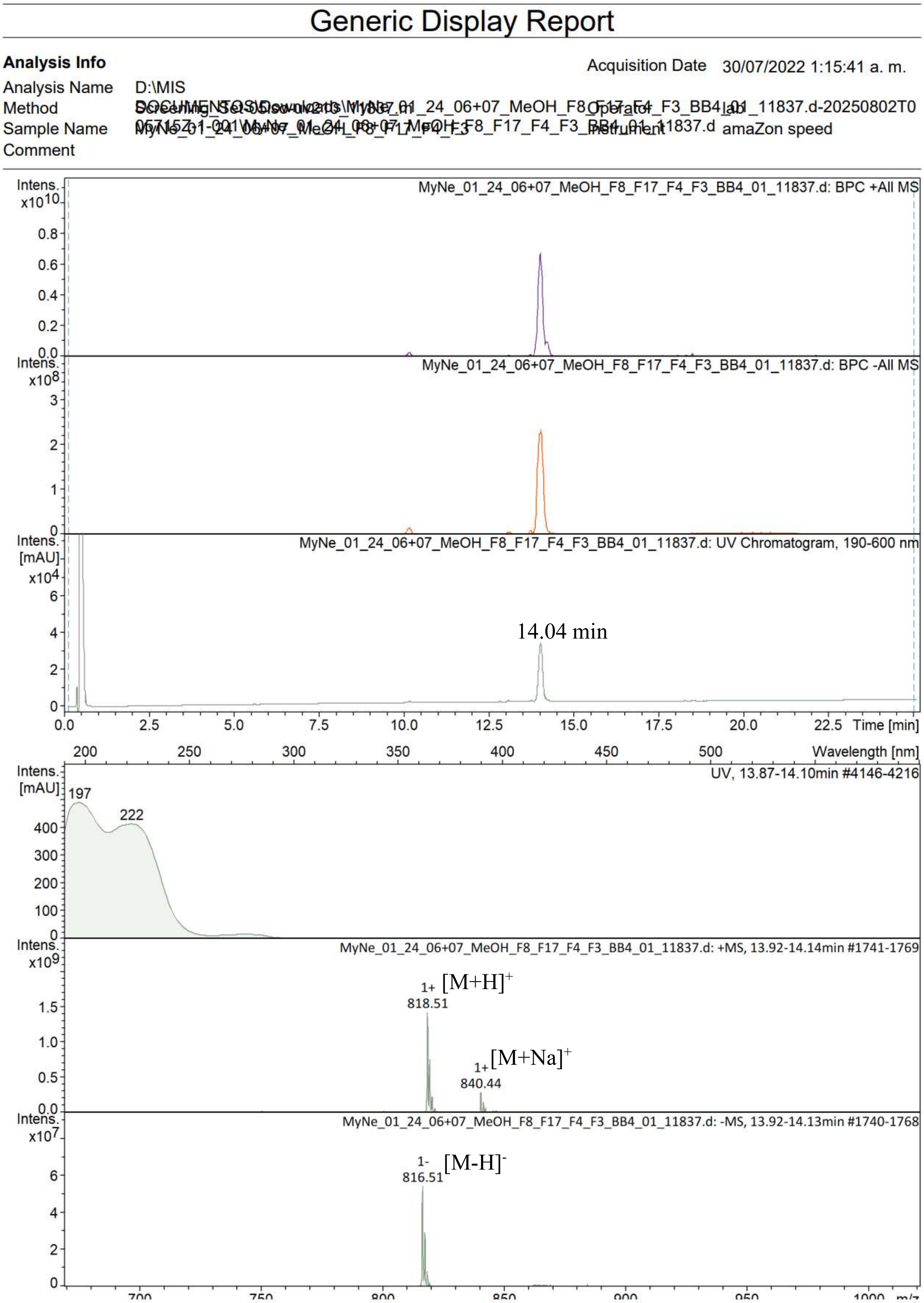

Figure S41. LR-ESI-MS spectrum of pullularin G (**6**).

Generic Display Report

Analysis Info

Analysis Name	S:\DATA\timsTOF\NLL21_Natalia Llanos\25_03\MyNe_01_24_06+07_MeOH_F8_F17_F4_F3	Acquisition Date	12-Dec-24 1:29:29 AM
Method	MWIS_BEH50mm_25min_IntThreshold 225.m	Operator	Admin
Sample Name	MyNe_01_24_06+07_MeOH_F8_F17_F4_F3	Instrument	timsTOF Pro 2
Comment			

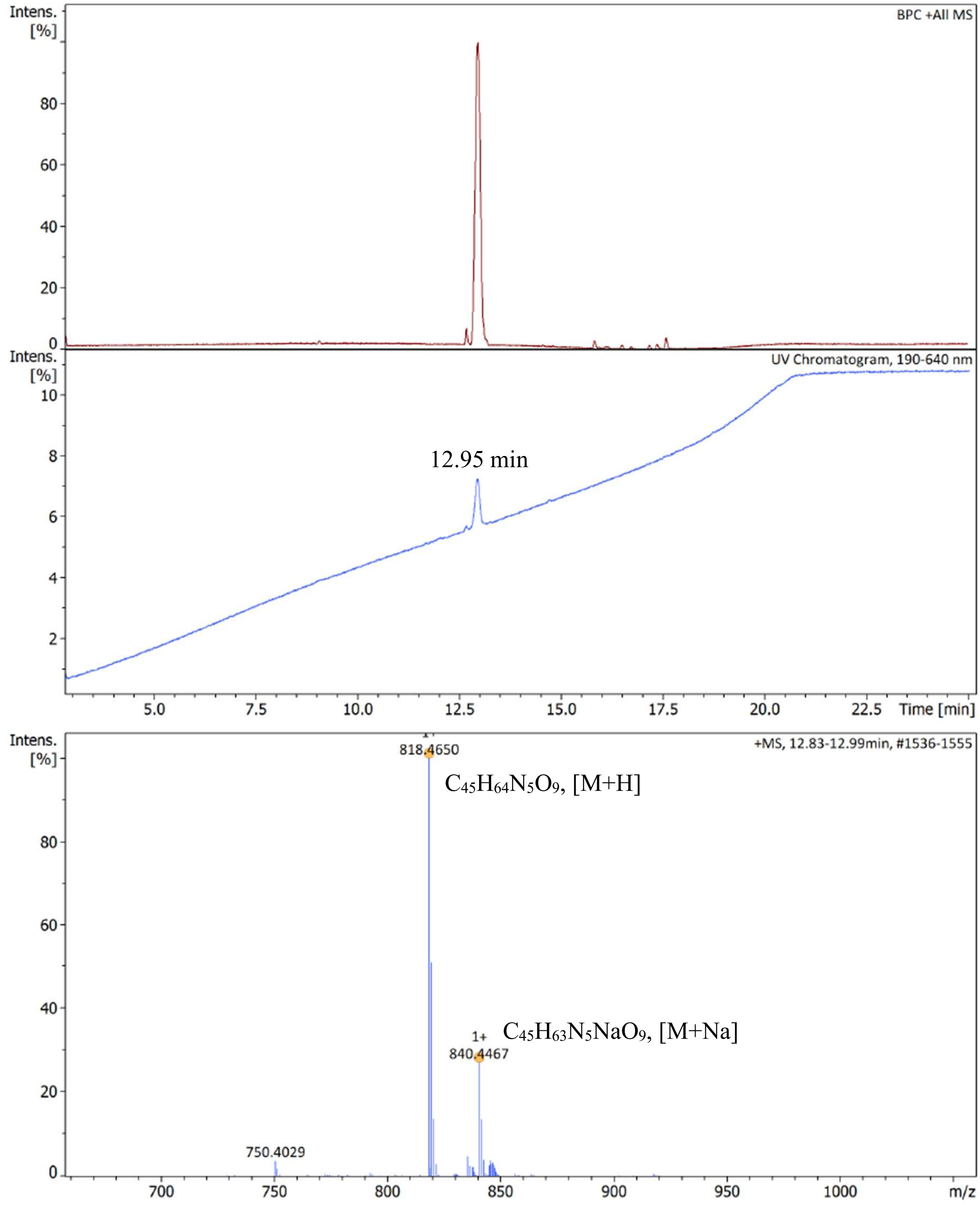

Bruker Compass DataAnalysis 6.1 printed: 01-Aug-25 2:44:02 PM by: nll21 Page 1 of 1

Figure S42. HR-ESI-MS spectrum of pullularin G (**6**).

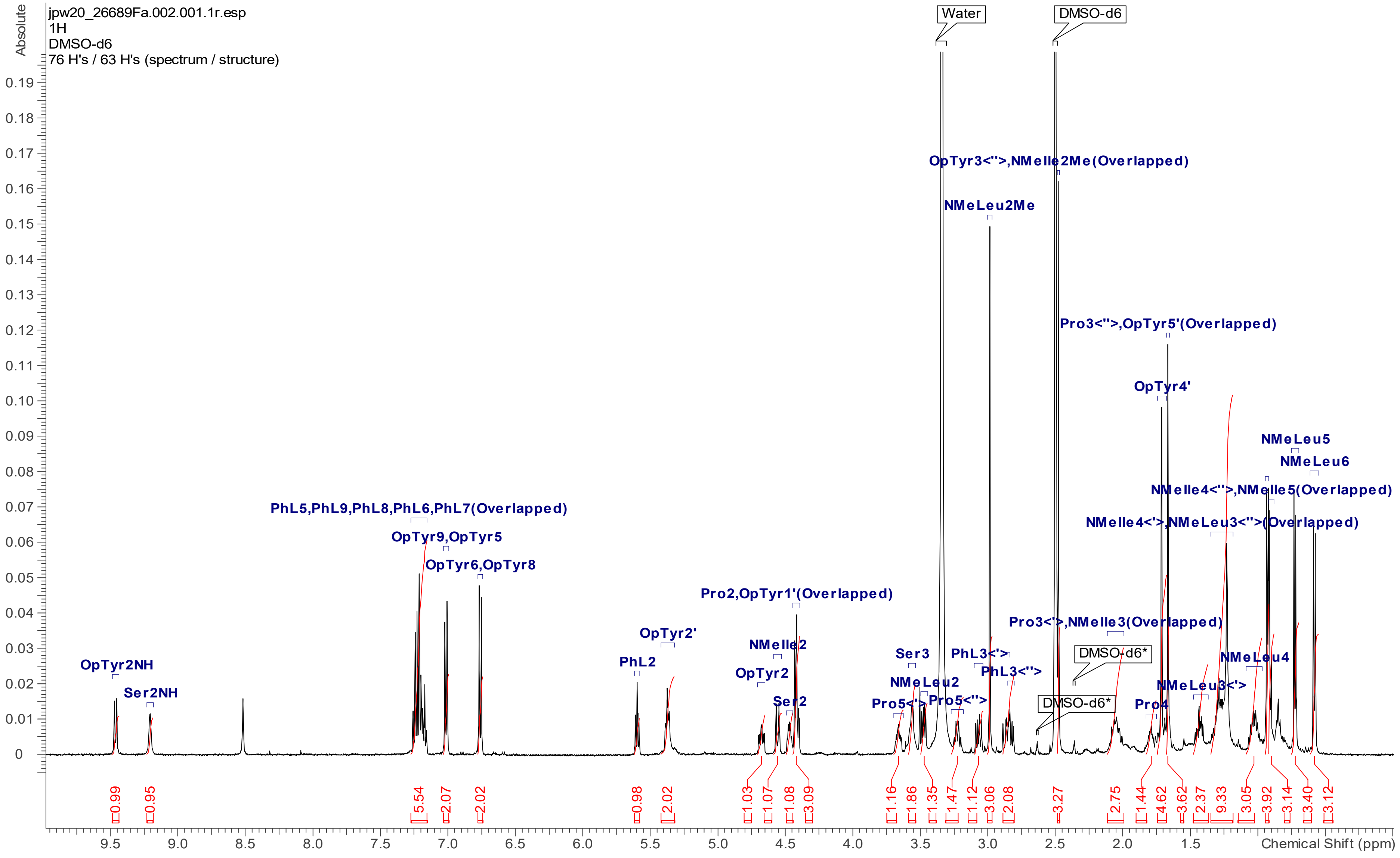

Figure S43. ^{1}H NMR spectrum of pullularin G (**6**) (500 MHz, DMSO-*d6*).

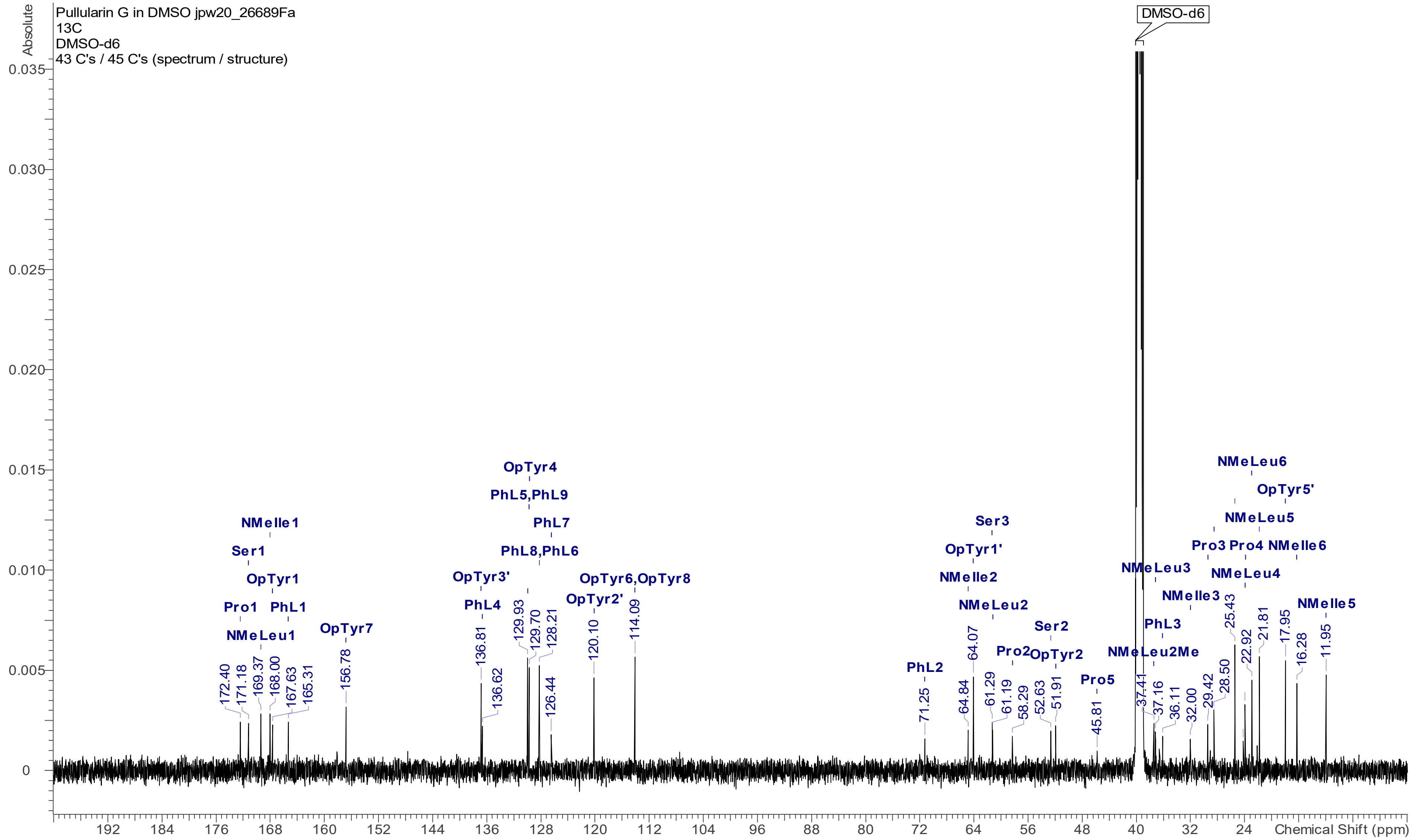

Figure S44. ^{13}C NMR spectrum of pullularin G (**6**) (125 MHz, DMSO-*d6*).

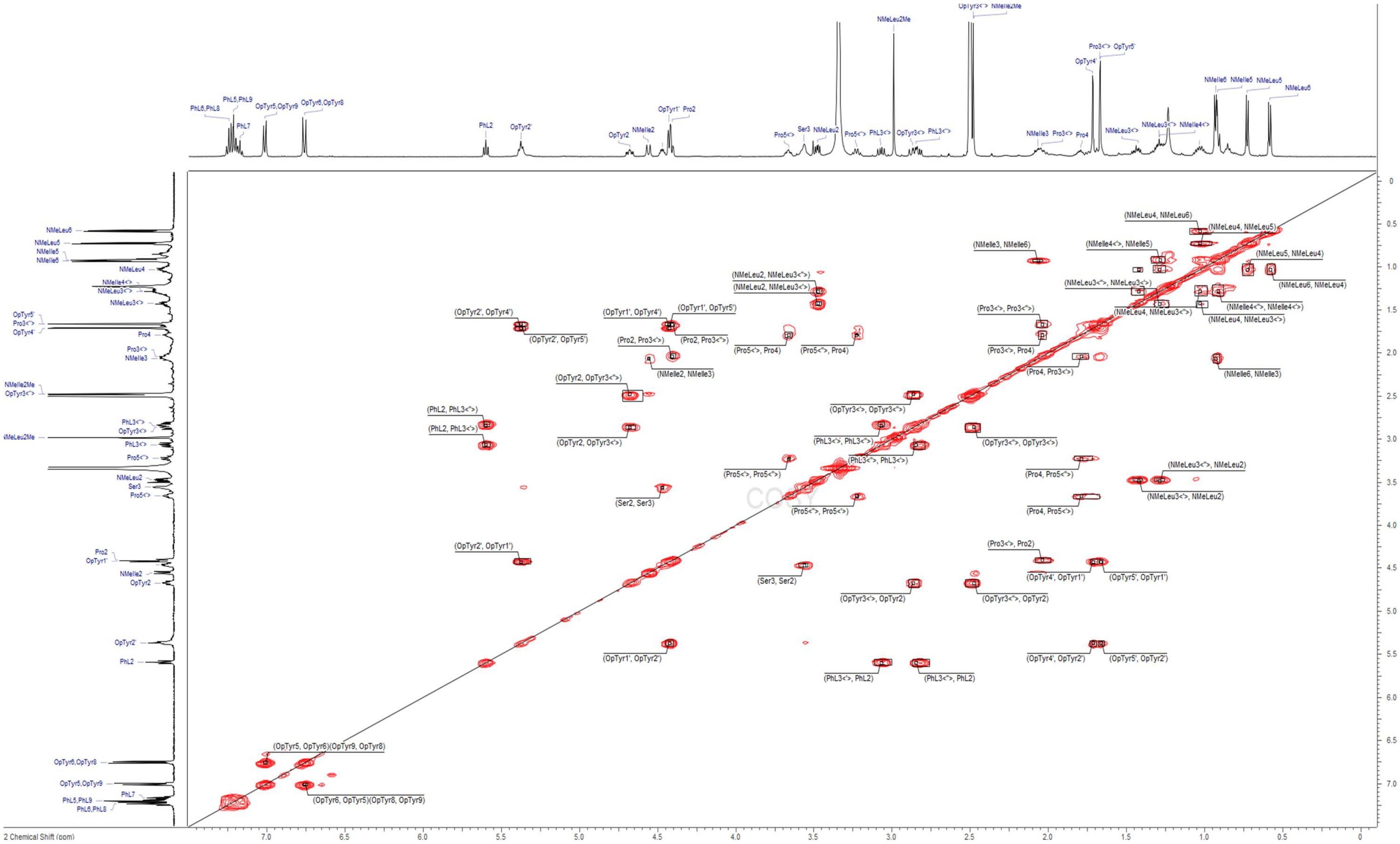

Figure S45. COSY NMR spectrum of pullularin G (**6**) (500 MHz, DMSO-*d6*).

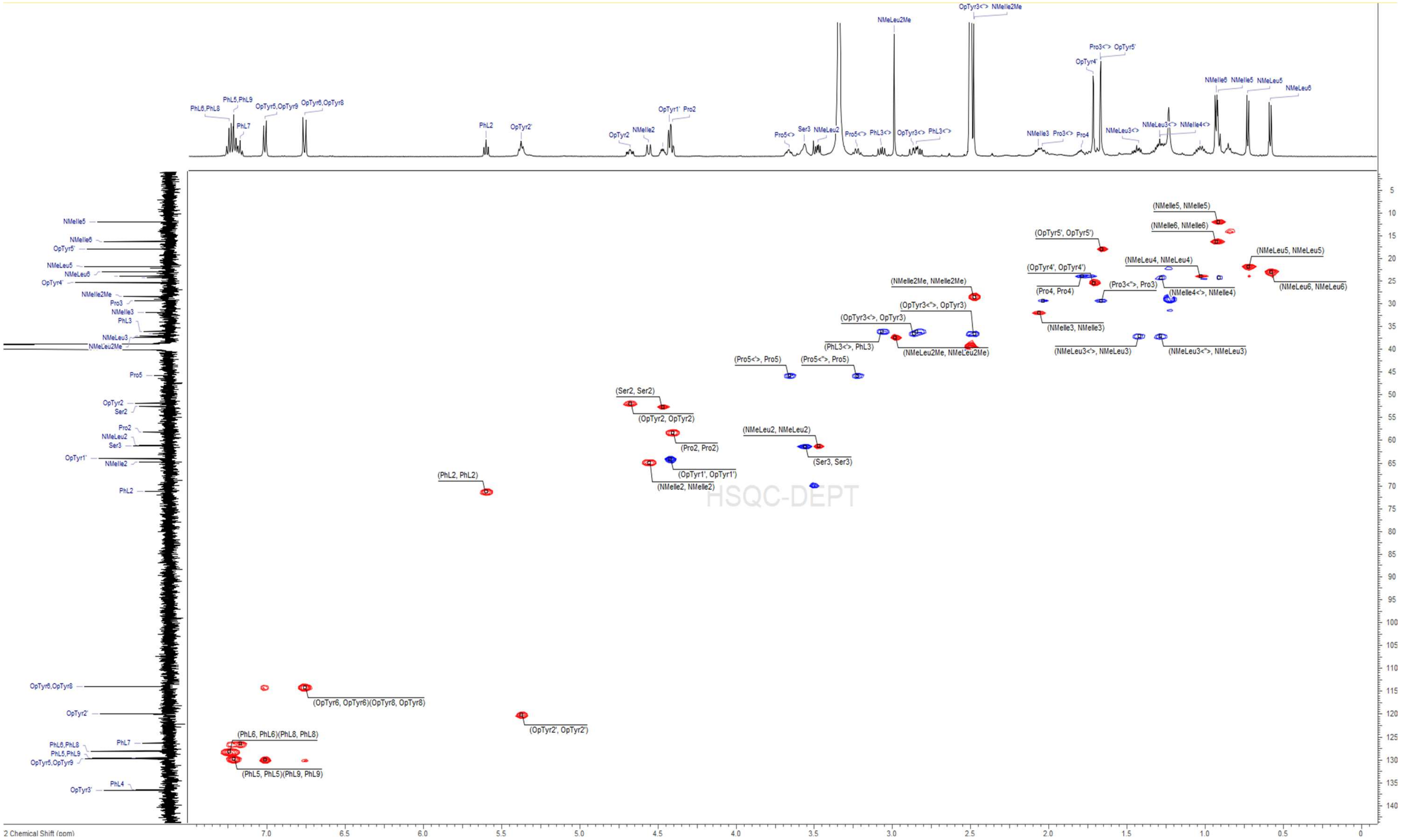

Figure S46. HSQC NMR spectrum of pullularin G (**6**) (500 MHz, DMSO-*d6*).

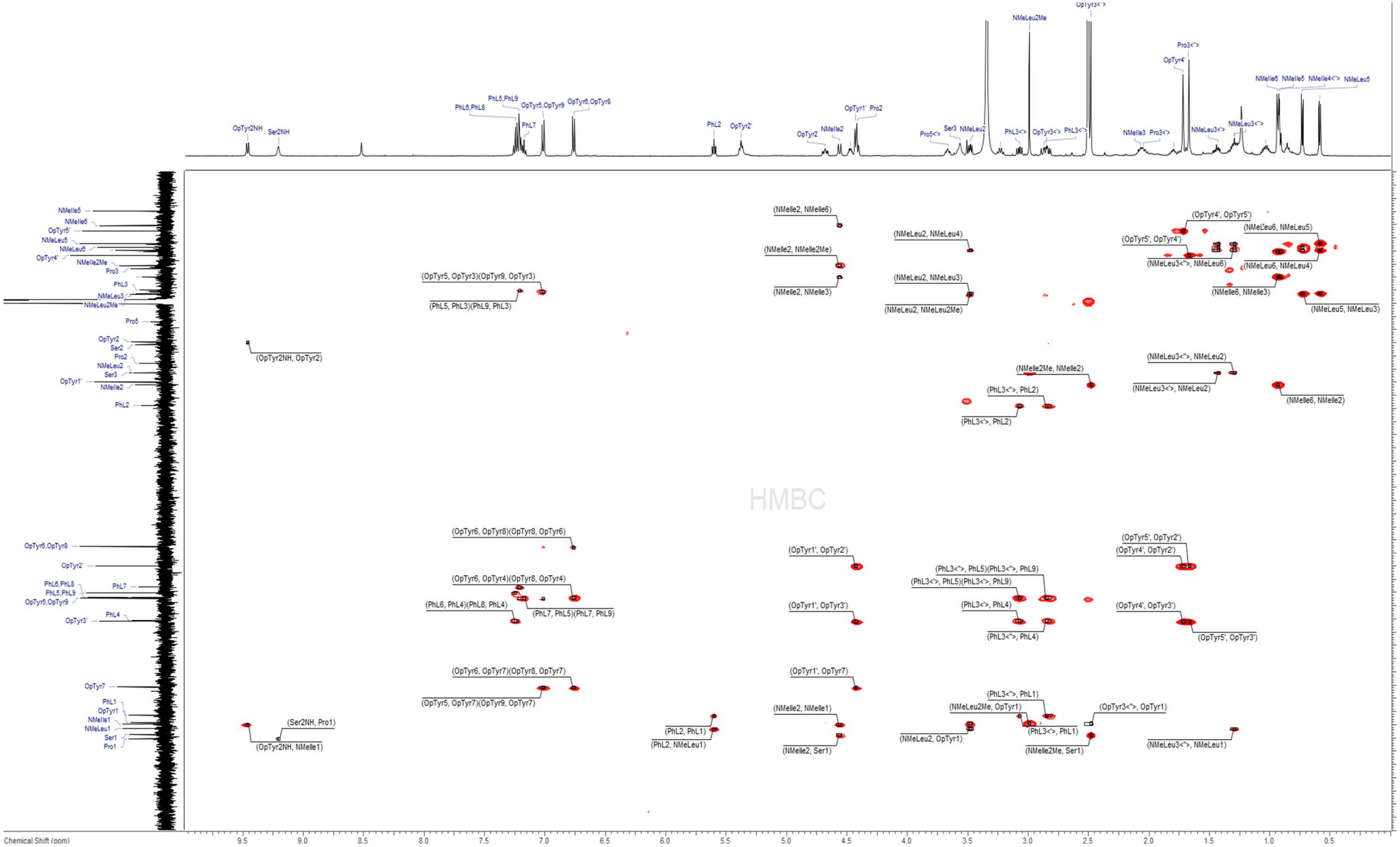

Figure S47. HMBC NMR spectrum of pullularin G (**6**) (500 MHz, DMSO-*d6*).

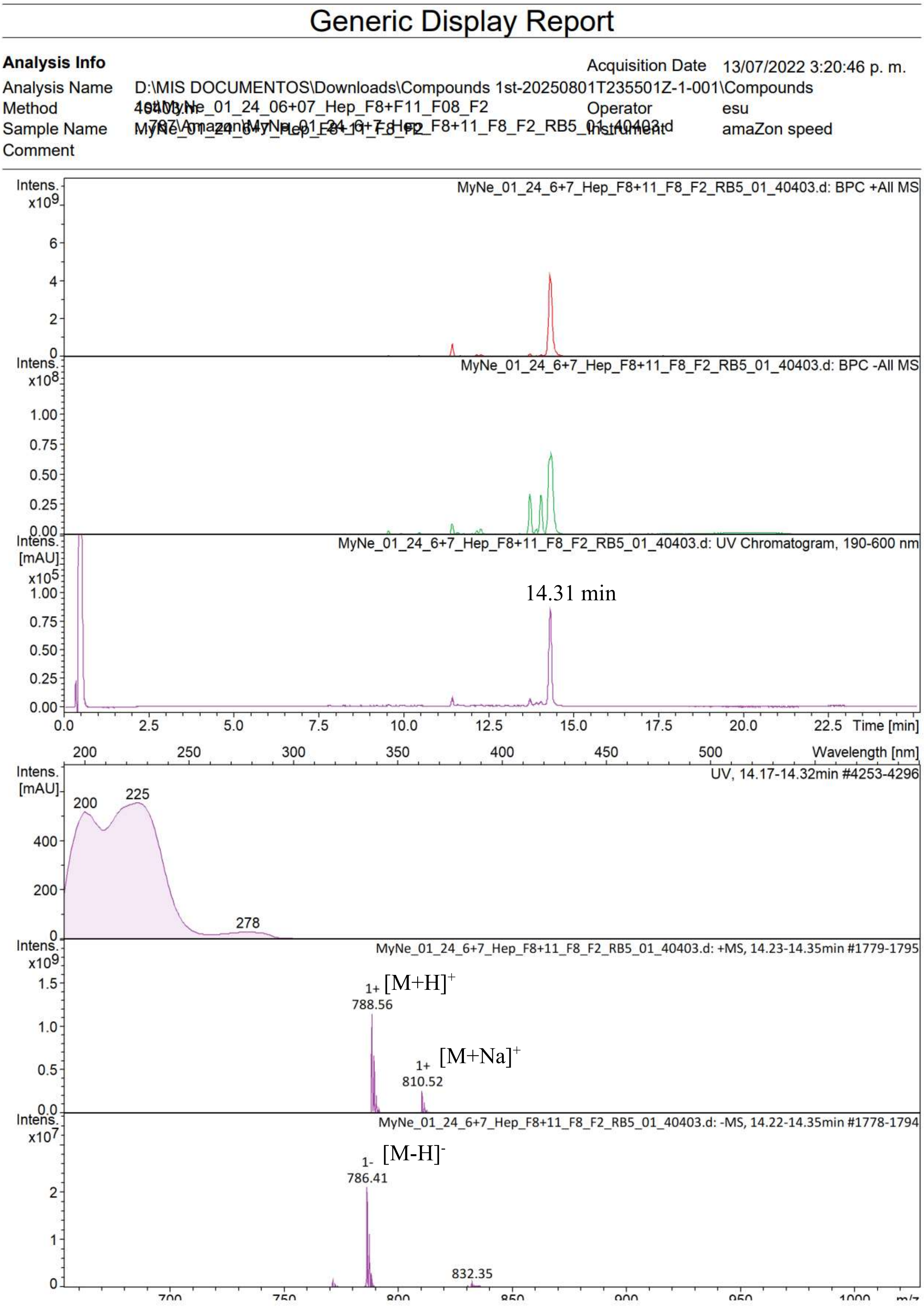

Figure S48. LR-ESI-MS spectrum of pullularin H (**7**).

Generic Display Report

Analysis Info

Analysis Name	S:\DATA\timsTOF\NLL21_Natalia Llanos\25_03\MyNe_01_24_06+07_Hep_F8+11_F8_F2_Frac.d	Acquisition Date	12-Dec-24 12:58:34 AM
Method	MWIS_BEH50mm_25min_IntThreshold 225.m	Operator	Admin
Sample Name	MyNe_01_24_06+07_Hep_F8+11_F8_F2	Instrument	timsTOF Pro 2
Comment			

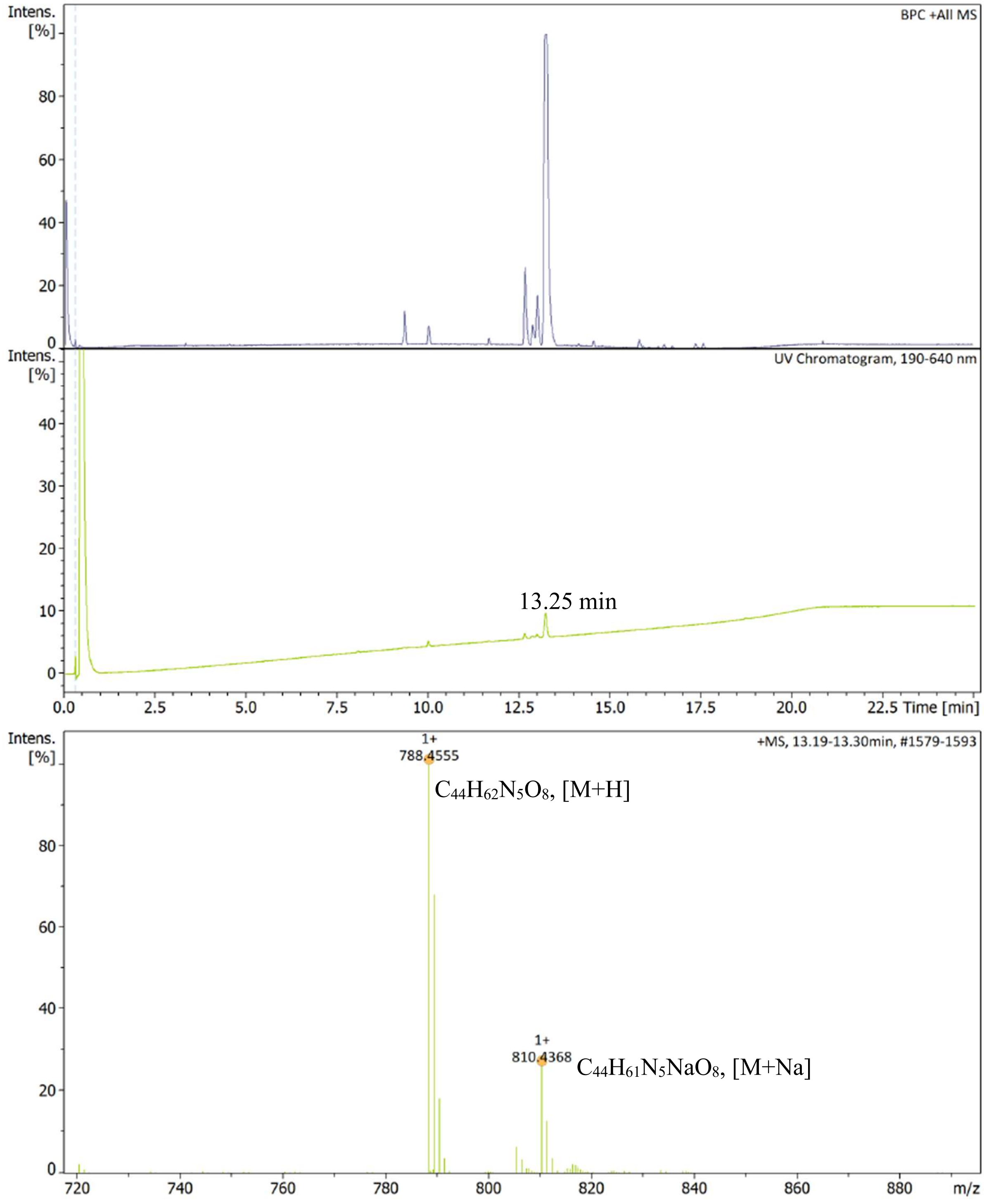

Bruker Compass DataAnalysis 6.1 printed: 01-Aug-25 3:03:35 PM by: nll21 Page 1 of 1

Figure S49. HR-ESI-MS spectrum of pullularin H (**7**).

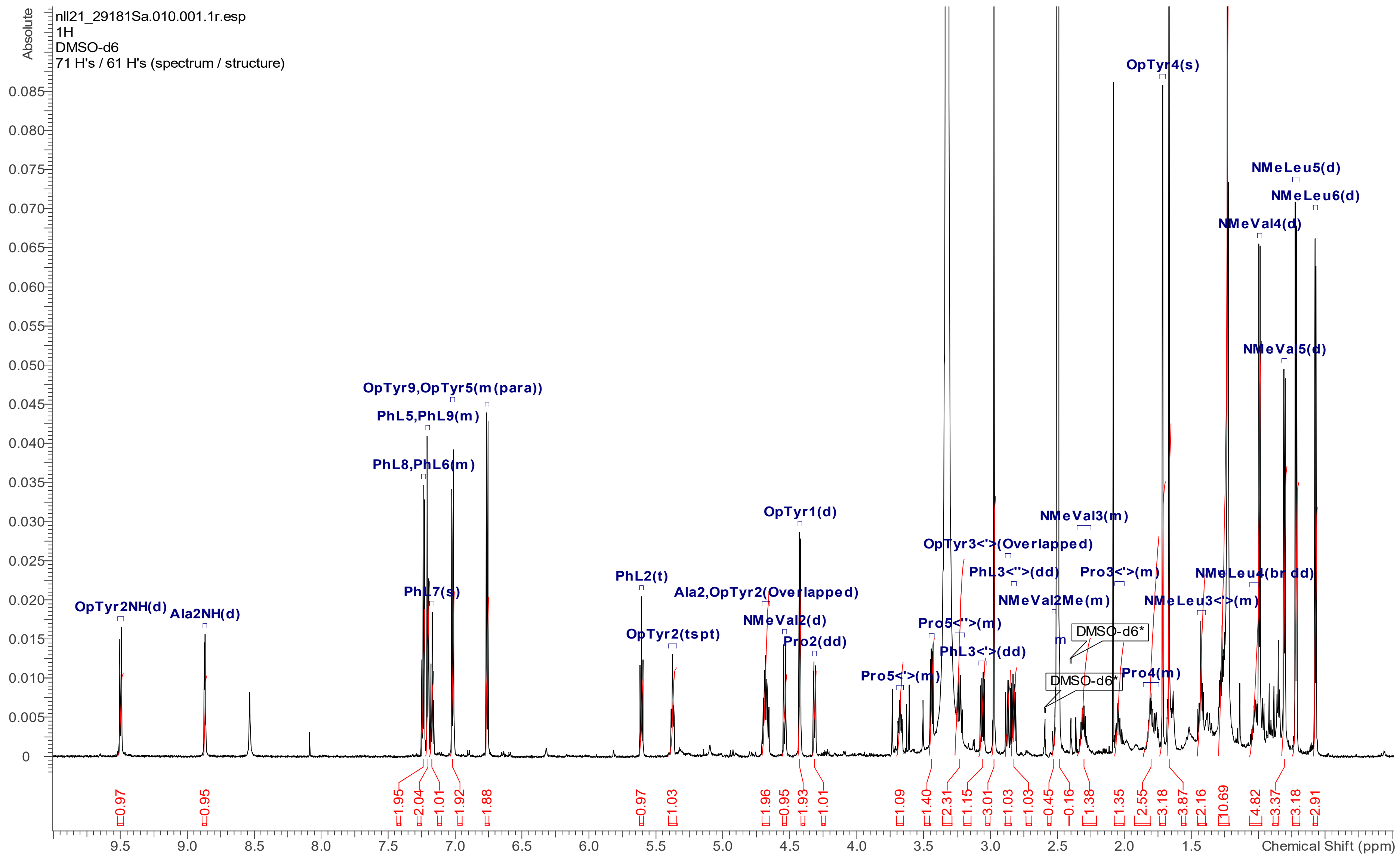

Figure S50. ^{1}H NMR spectrum of pullularin H (**7**) (700 MHz, DMSO-*d6*).

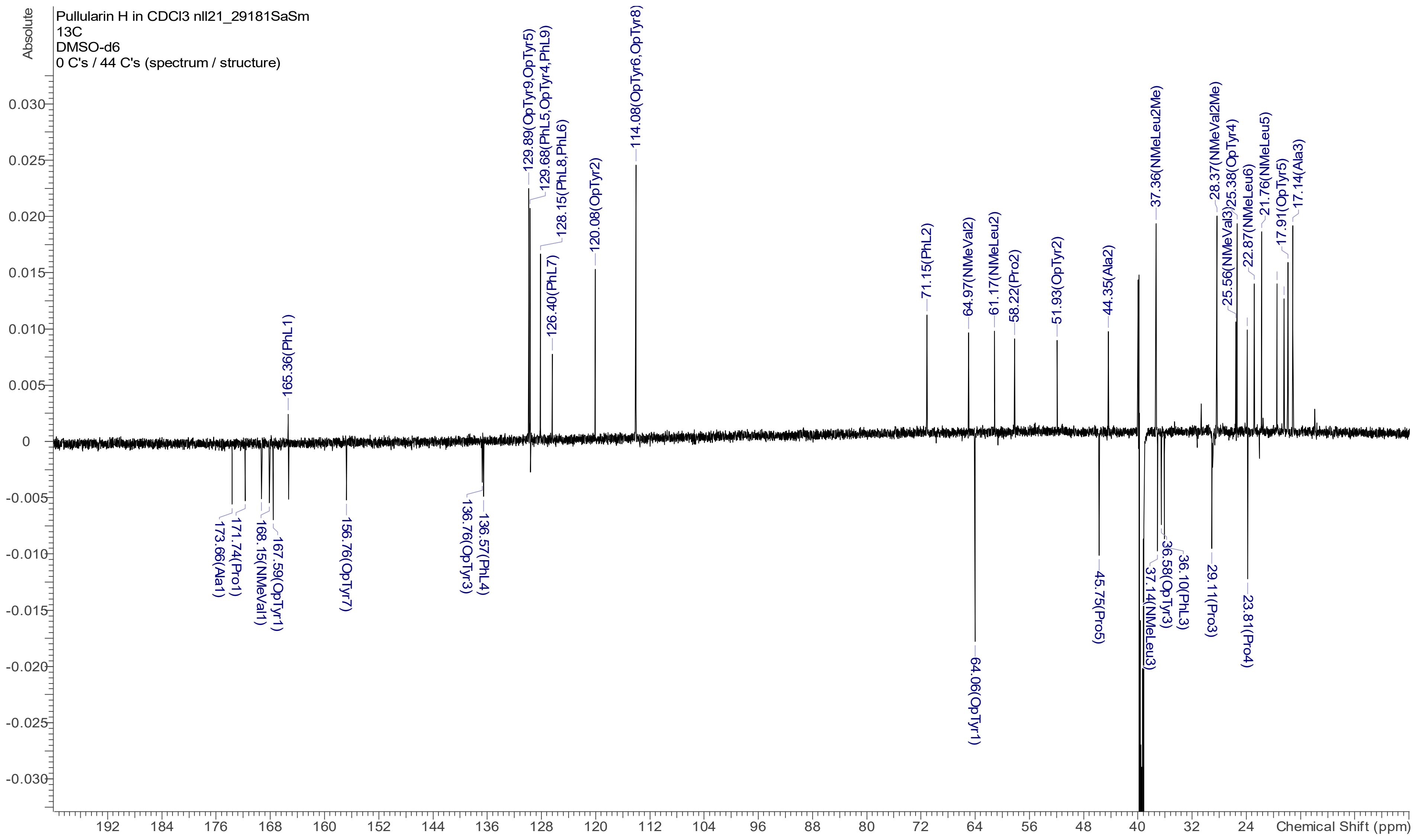

Figure S51. APT NMR spectrum of pullularin H (**7**) (175 MHz, DMSO-*d6*).

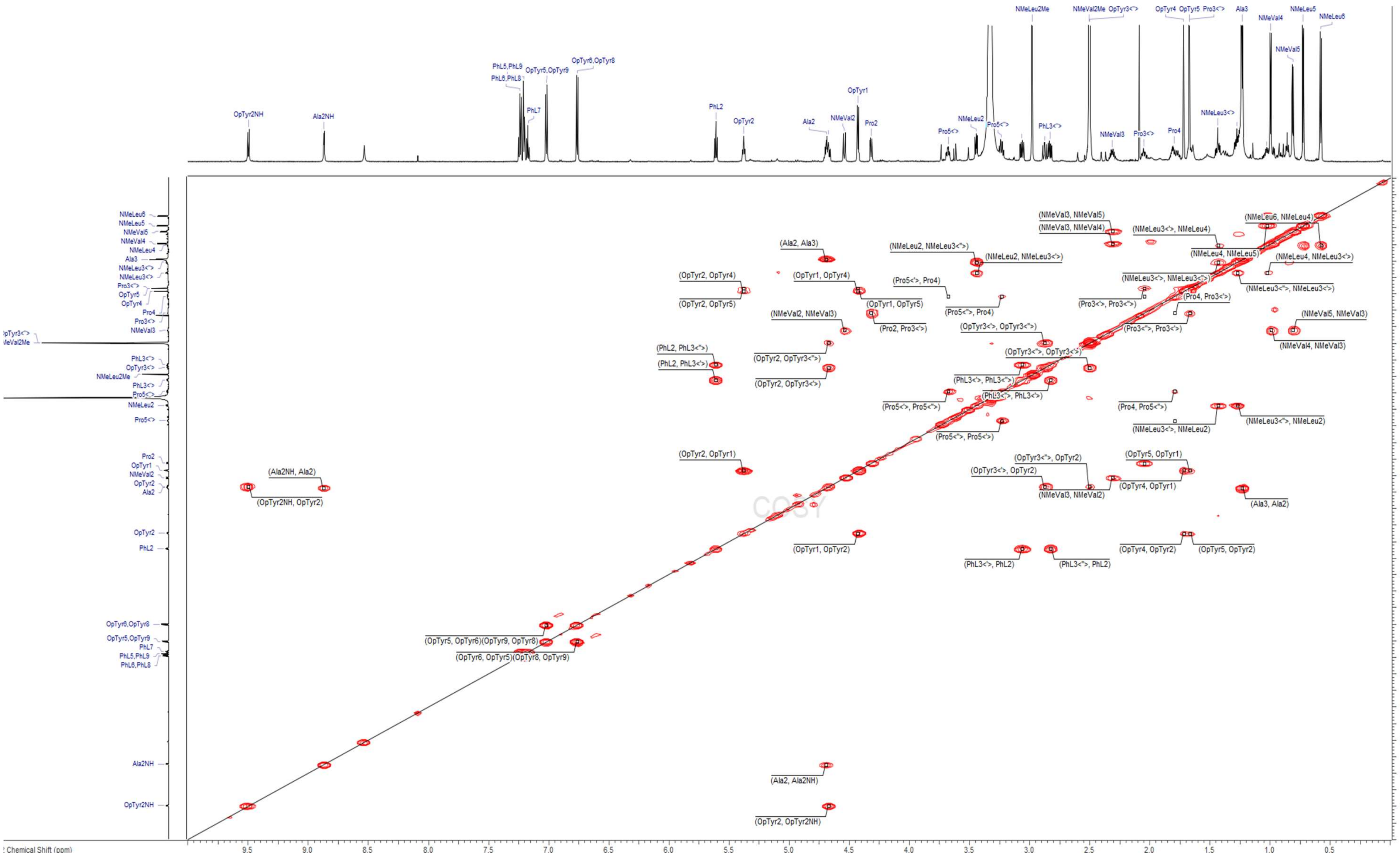

Figure 52. COSY NMR spectrum of pullularin H (**7**) (700 MHz, DMSO-*d6*).

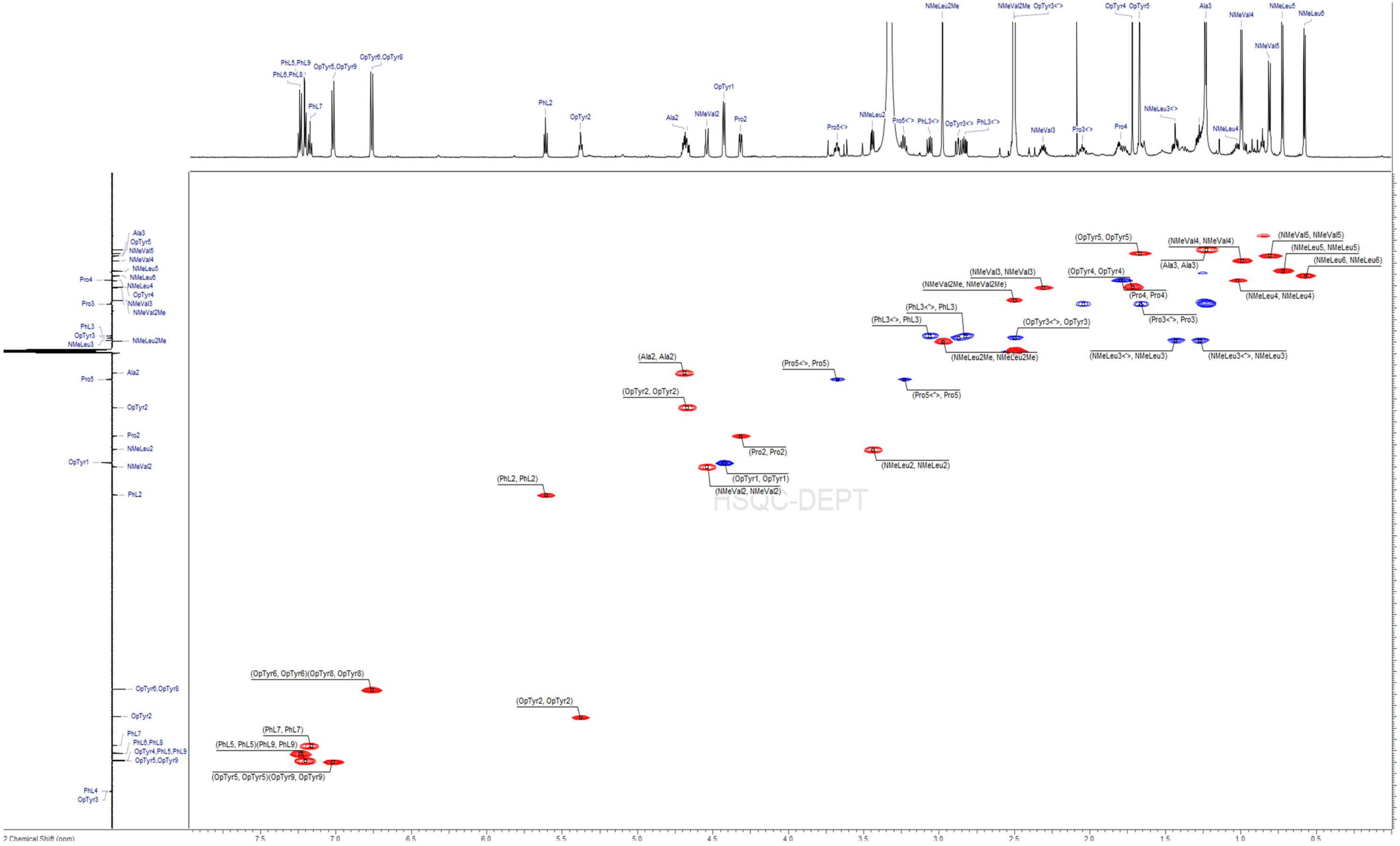

Figure S53. HSQC NMR spectrum of pullularin H (**7**) (700 MHz, DMSO-*d6*).

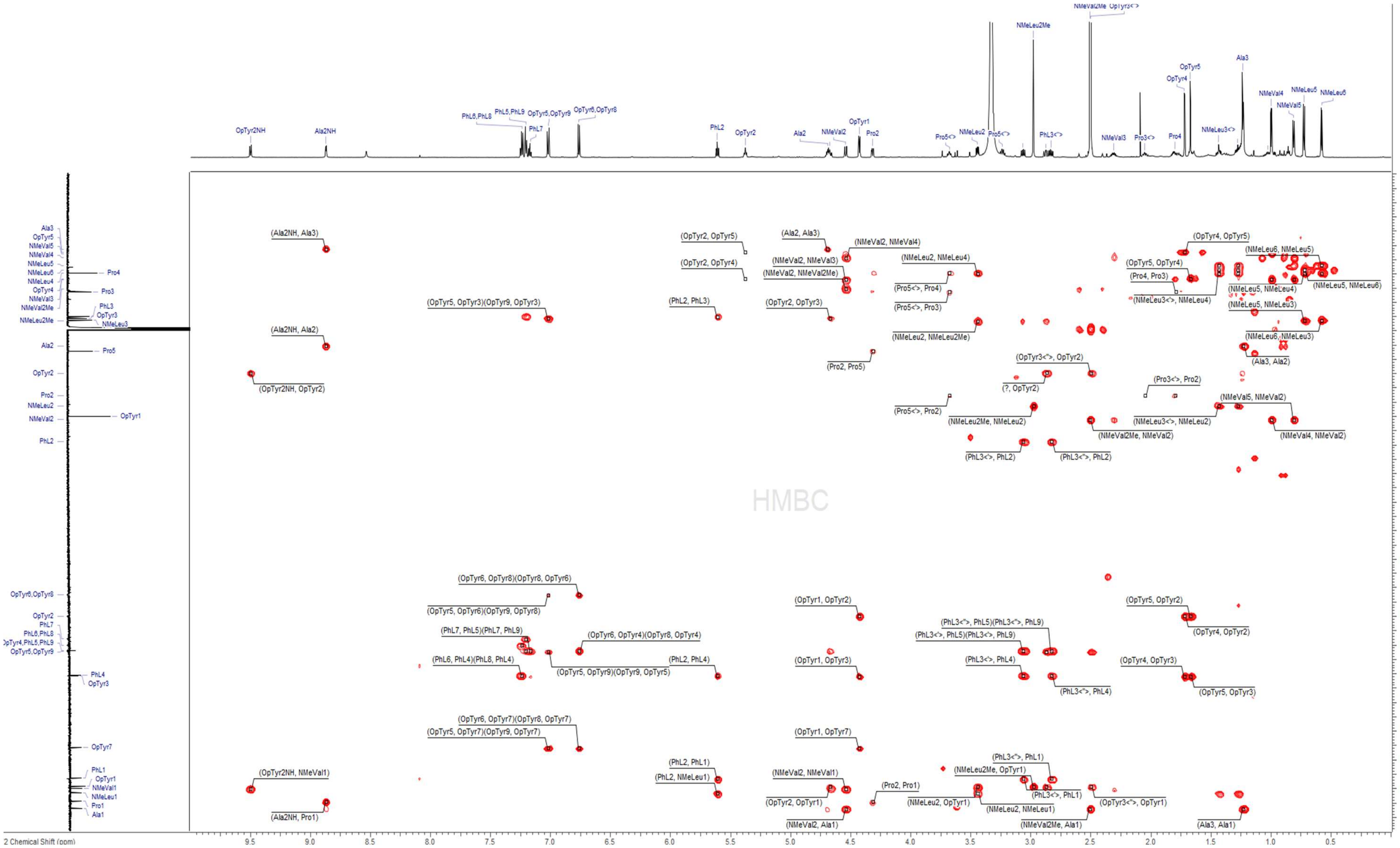

Figure S54. HMBC NMR spectrum of pullularin H (**7**) (700 MHz, DMSO-*d6*).

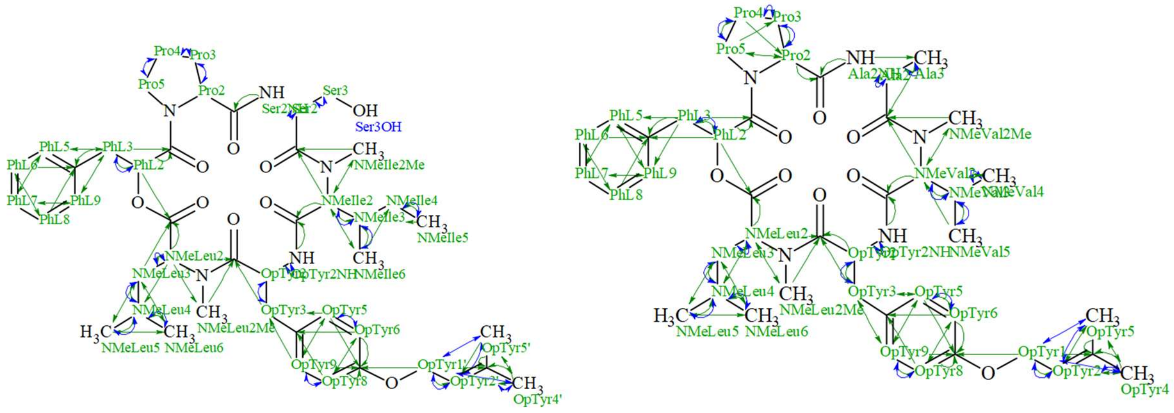

Figure S55. Key COSY (blue arrows) and HMBC (green arrows) accounting for the structure elucidation of pullularins G and H (**6** and **7**).

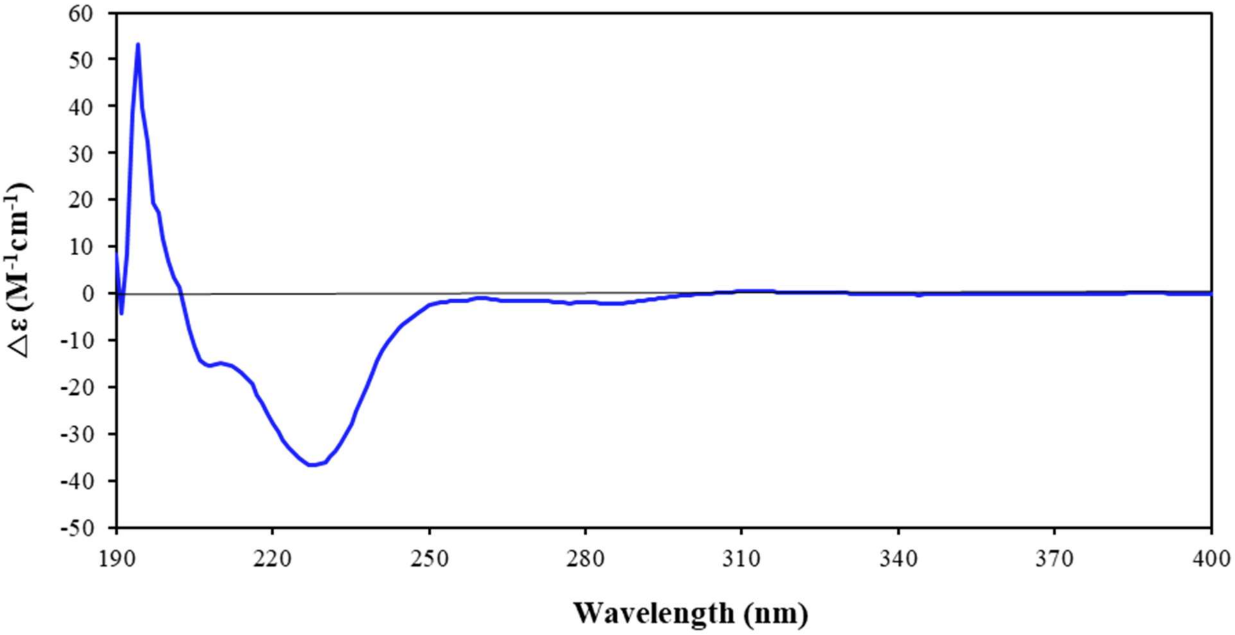

Figure S56. Experimental ECD spectrum of pullularin G (**6**) in methanol.

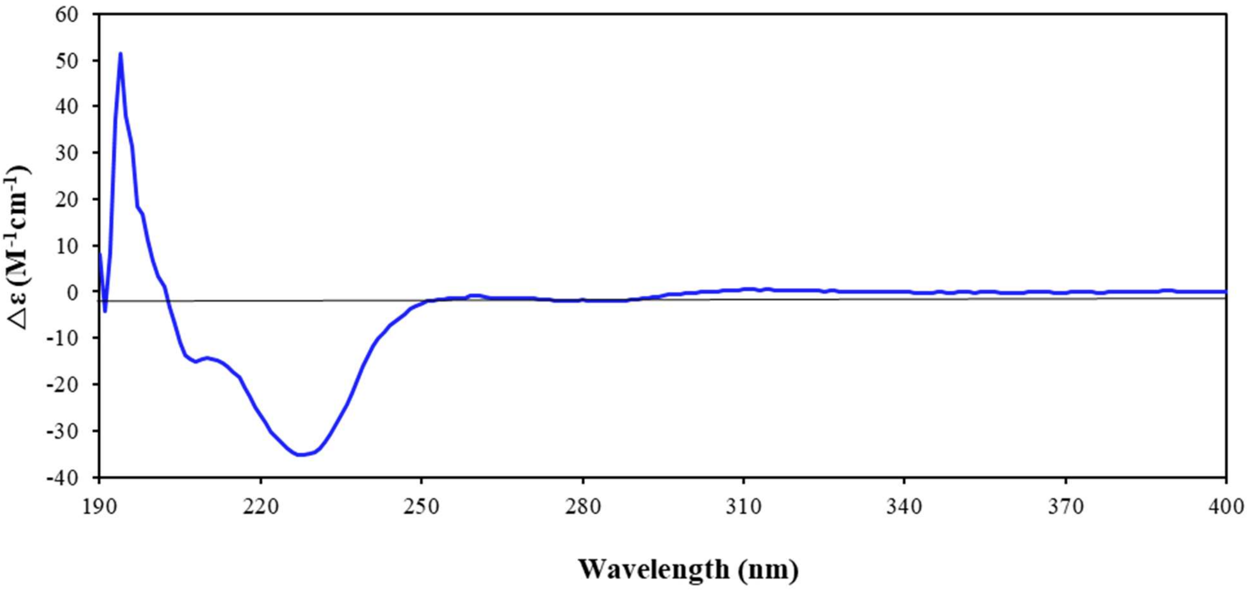

Figure S57. Experimental ECD spectrum of pullularin H (**7**) in methanol.

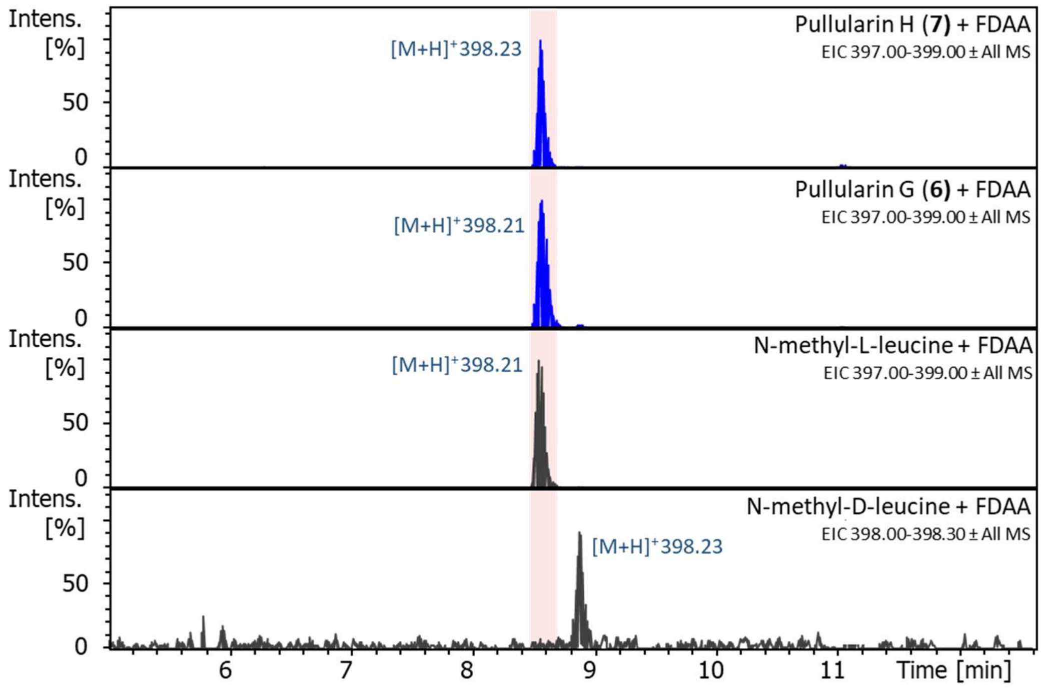

Figure S58. LC-ESI-MS spectra of pullularins G and H (**6** and **7**), N-methyl-L-leucine, and N-methyl-D-leucine with FDAA. Top-bottom: Pullularin H (**7**) + FDAA, Pullularin G (**6**) + FDAA, N-methyl-L-leucine + FDAA, N-methyl-D-leucine + FDAA. $[M+H]^+$ of the adducts is displayed in blue. Extracted ion chromatogram (*m/z*: 397-399) and UV-chromatogram at 190-600 nm are shown. Bar indicates identical MS-Peaks (pink N-methyl-L-leucine).

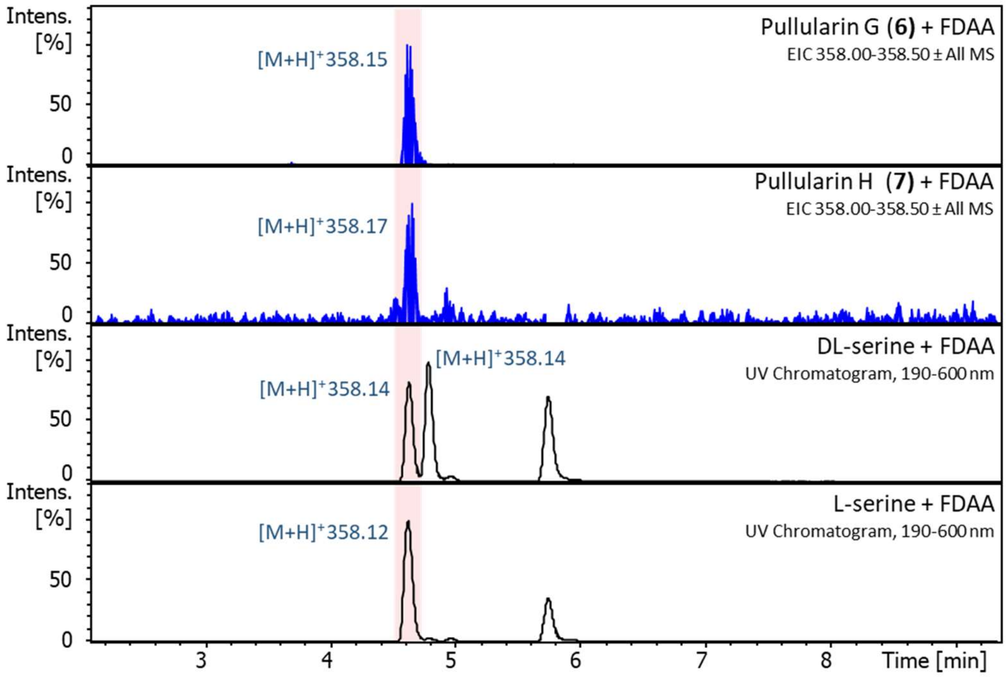

Figure S59. LC-ESI-MS spectra of pullularins G and H (**6** and **7**), DL-serine, and L-serine with FDAA. Top-bottom: Pullularin G (**6**) + FDAA, Pullularin H (**7**) + FDAA, DL-serine + FDAA, L-serine + FDAA. $[M+H]^+$ of the adducts is displayed in blue. Extracted ion chromatogram (*m/z*: 358-358.5) and UV-chromatogram at 190- 600 nm are shown. Bar indicates identical MS-Peaks (pink L-serine).

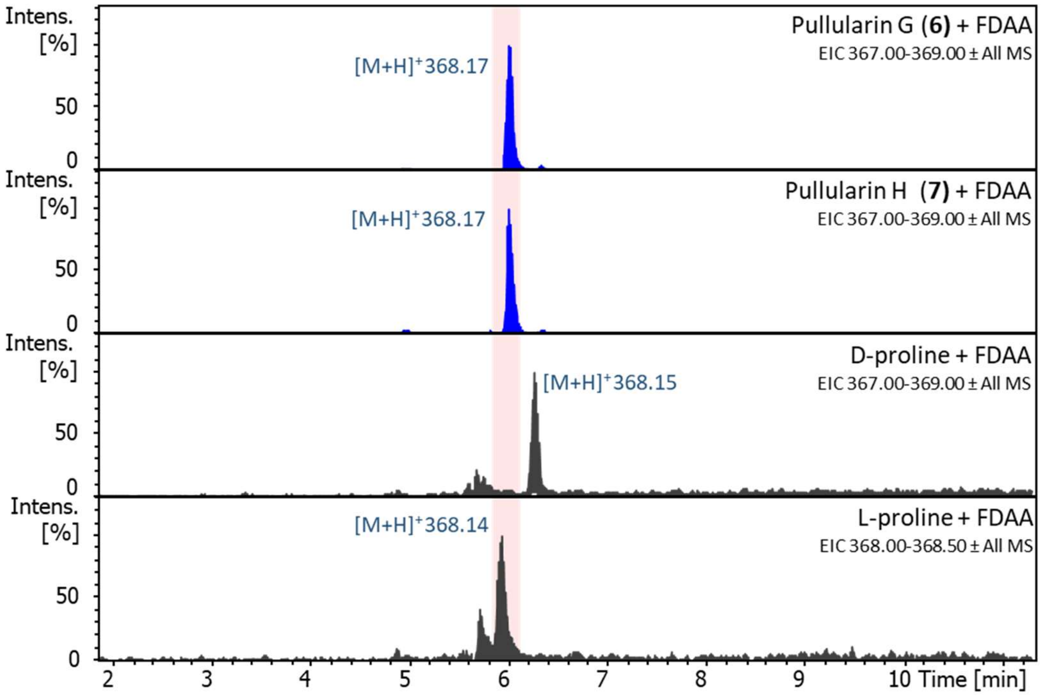

Figure S60. LC-ESI-MS spectra of pullularins G and H (**6** and **7**), D-proline, and L-proline with FDAA. Top-bottom: Pullularin G (**6**) + FDAA, Pullularin H (**7**) + FDAA, D-proline + FDAA, L-proline + FDAA. $[M+H]^+$ of the adducts is displayed in blue. Extracted ion chromatogram (*m/z*: 367-369) and UV-chromatogram at 190- 600 nm are shown. Bar indicates identical MS-Peaks (pink L-proline).

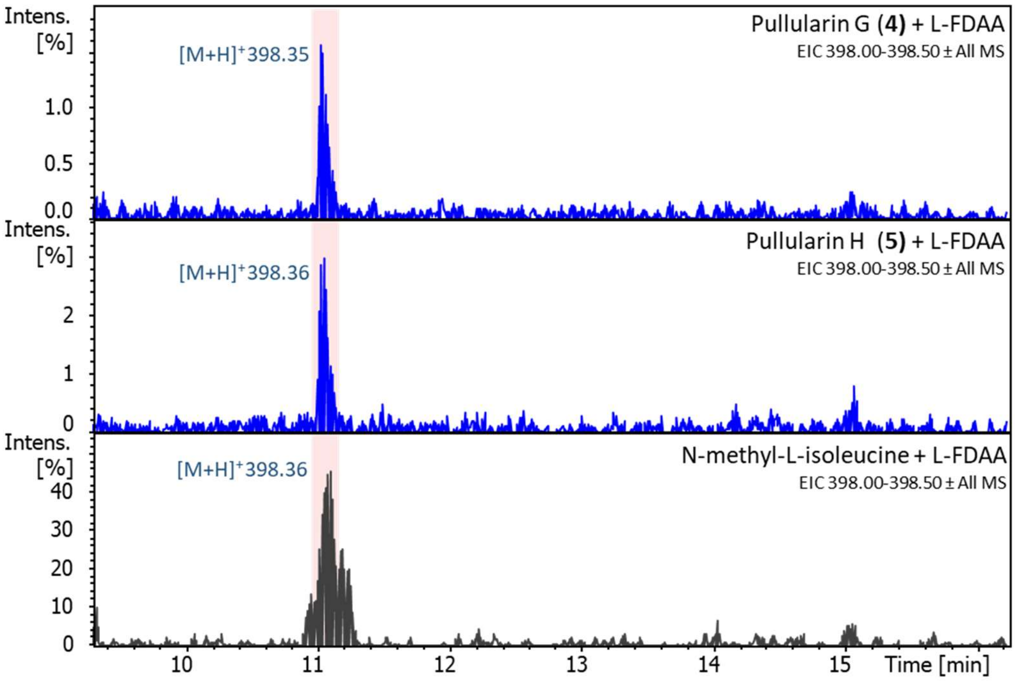

Figure S61. LC-ESI-MS spectra of pullularins G and H (**6** and **7**), and N-methyl-L-isoleucine with FDAA. Top-bottom: Pullularin G (**6**) + FDAA, Pullularin H (**7**) + FDAA, N-methyl-L-isoleucine + FDAA. $[M+H]^+$ of the adducts is displayed in blue. Extracted ion chromatogram (*m/z*: 398-398.5) and UV-chromatogram at 190- 600 nm are shown. Bar indicates identical MS-Peaks (pink N-methyl-L-isoleucine).

Isolation procedure of compounds from *P. karssenii* strain JKI 73120

The methanol extracts obtained from strain JKI 73120, grown in BRFT and WOFT for 2–6 weeks as described in Section 2.3, showed a similar metabolite profile according to HPLC-DAD/MS analyses and were therefore combined. The extract (3.2 g) was fractionated using a Büchi Grace Reveleris X2 flash chromatography system operating in normal-phase mode. An 80 g silica cartridge (FlashPure ID Silica, Büchi) was used for the separation. For elution, three solvent systems were employed: Solvent A was *n*-heptane with 0.1% FA; Solvent B contained 58% *n*-heptane, 40% tert-butyl methyl ether (TBME), 2% MeOH with 0.1% FA; and Solvent C consisted of MeOH with 0.1% FA. The flow rate was set to 60 mL/min. The separation followed a multistep gradient: starting with solvent system AB, isocratic conditions at 0% B from 0 to 3 min, then a linear increase to 100% B over 15 min, maintained at 100% B for 3 min, followed by a shift to BC, increasing to 50% C in 10 min and isocratic at 50% C for 3 min. Fractions were collected for further analysis and purification based on their retention times and chromatographic profiles. This separation yielded a fraction of 2.3 g (retention time (t_R) = 22.6–26.0 min), which was further separated using a preparative reversed-phase HPLC (Pure C-850, Büchi). For this, a Gemini 10 µm C18 110 Å column (250 x 50 mm, Phenomenex, Aschaffenburg, Germany) was used as the stationary phase. The mobile phase consisted of MilliQ water + 0.1% FA (solvent A) and MeCN + 0.1% FA (solvent B). The gradient elution started with 15% B for 3 min, then increased from 15% B to 50% B over 3 min, followed by a rise from 50% B to 60% B over 20 min, from 60% B to 80% B over 15 min, and from 80% B

to 100% B over 10 min, and finally held isocratically at 100% B for 5 min. The flow rate was maintained at 50 mL/min, and four runs were performed to complete the separation. UV detection was conducted at 210, 254, 300, and 350 nm, and major fractions (F16–F19) were collected based on chromatographic peaks observed.

Fraction F16 (217.5 mg, t_R = 46.6–48.0 min) was subjected to fractionation using a Gilson PLC 2050 (Gilson, Middleton, WI, USA) preparative HPLC system configured in reversed-phase mode. A Nucleodur C18 ec 110 Å column (150 × 40 mm, 10 µm, Macherey-Nagel, Düren, Germany) served as the stationary phase. Separation was performed at a flow rate of 40 mL/min employing a solvent system of MilliQ water with 0.1% FA as solvent A and MeCN with 0.1% FA as solvent B. The gradient elution profile was initiated with an isocratic hold at 60% B from 0 to 5 min, followed by an increase to 75% B over 40 min, then a ramp to 100% B within 5 min, and finally maintained at 100% B for an additional 5 min. This chromatographic separation resulted in the isolation of compound **2** (22 mg; t_R = 16.8 min). Fraction F17 (590 mg, t_R = 48.1–51.1 min) was further separated using the same instruments and solvent system as described for F16, employing a Nucleodur C18 HTec 110 Å column (250 × 40 mm, 10 µm, Macherey-Nagel) and beginning with isocratic conditions at 70% B for 5 min, then increased to 90% B over 40 min, followed by a 5-min increase to 100% B, and maintained at 100% B for an additional 5 min. From this separation, a fraction of 39 mg with a t_R = 48.8–49.3 min was collected and further separated to obtain compound **6** (1.8 mg, t_R = 47.1 min). The reversed-phase preparative system Büchi Pure C-850 Flash Prep was employed for the separation, and an XBridge BEH 5 µm C18 column (250 × 19 mm; Waters, Eschborn, Germany) was used as the stationary phase. Solvents A and B were used as the mobile phase, with a flow rate of 20 mL/min. The elution gradient was initiated at 52% B for 5 min, followed by an increase to 55% B over 50 min, then to 100% B over 10 min, and was held isocratically at 100% B for 5 min.

Alongside the methanol phase purification, the crude extracts from the *n*-heptane phase of strain JKI 73120, obtained from solid-state fermentation from both media, as described in Section 2.3, were combined as they exhibited a similar metabolite profile based on HPLC-DAD/MS analyses. The extract (3.6 g) was subjected to a Flash chromatography system (Grace Reveleris X2, Büchi) operating in normal-phase mode. The separation was carried out using an 80 g silica cartridge (FlashPure ID Silica, Büchi) as the stationary phase. Three solvent mixtures were used as eluents: solvent A (*n*-heptane + 0.1% FA), solvent B (58% *n*-heptane, 40% TBME, and 2% MeOH + 0.1% FA), and solvent C (37.5% acetone, 37.5% dichloromethane (DCM), and 25% MeOH + 0.1% FA). The flow rate was maintained at 60 mL/min. Separation was initiated under isocratic conditions with 0% AB for 3 min, followed by a 15-min increase to 100% AB, and then maintained at 100% AB for an additional 3 min. The mobile phase composition was then switched from 0% to 100% BC over 10 min, followed by a hold at 100% BC for 3 min. Collected fractions were monitored by UV detection (210 nm) and pooled based on their chromatographic profiles, resulting in four major pooled fractions (G8–G11).

Fractions eluting between 13.7 and 17.5 min (G8) and from 23.1 and 25.6 min (G11) were combined and dried, yielding a 539 mg sample. This sample was subjected to further separation by preparative reversed-phase HPLC (C-850, Büchi). The stationary phase was a Luna 5 μm C18 110 Å column (250 × 21.2 mm; Phenomenex, Aschaffenburg, Germany). MilliQ water with 0.1% FA (solvent A) and MeCN with 0.1% FA (solvent B) served as the mobile phase delivered at a flow rate of 20 mL/min. The gradient started at 60% B for 5 min, increased to 80% B over 20 min, then to 100% B over 5 min, followed by a final isocratic step at 100% B for 15 min. A subfraction (20 mg, t_R = 26.0–26.7 min) was collected and subjected to further separation. Compounds **3** (12 mg, t_R = 38.6 min) and **7** (0.6 mg, t_R = 43.5 min) were isolated using a Gilson PLC 2050 preparative reversed-phase system with an XBridge BEH 5 μm C18 column (250 × 19 mm; ucleo). The mobile phase remained the same (solvents A and B) with a flow rate of 20 mL/min. The elution profile included an initial isocratic step at 50% B for 5 min, a linear increase to 55% B over 5 min, a 30-minute hold at 55% B, followed by a gradient to 100% B over 5 min, and a final isocratic step at 100% B for 5 min. Fraction G9 (42 mg, t_R = 17.5–19.2 min) was further separated using a Gilson PLC 2050 preparative HPLC system with a Luna 5 μm C18 110 Å column (250 × 21.2 mm; Phenomenex). Solvents A and B were used as the eluents at a flow rate of 20 mL/min. The gradient elution started with isocratic conditions at 65% B for 30 min, then increased to 100% B over 5 min, and was maintained at 100% B for an additional 10 min. This separation yielded 8 mg of compound **5** (t_R = 35.7 min). Further details about the purification data are available in (Scheme S1 and Tables S3–S11).

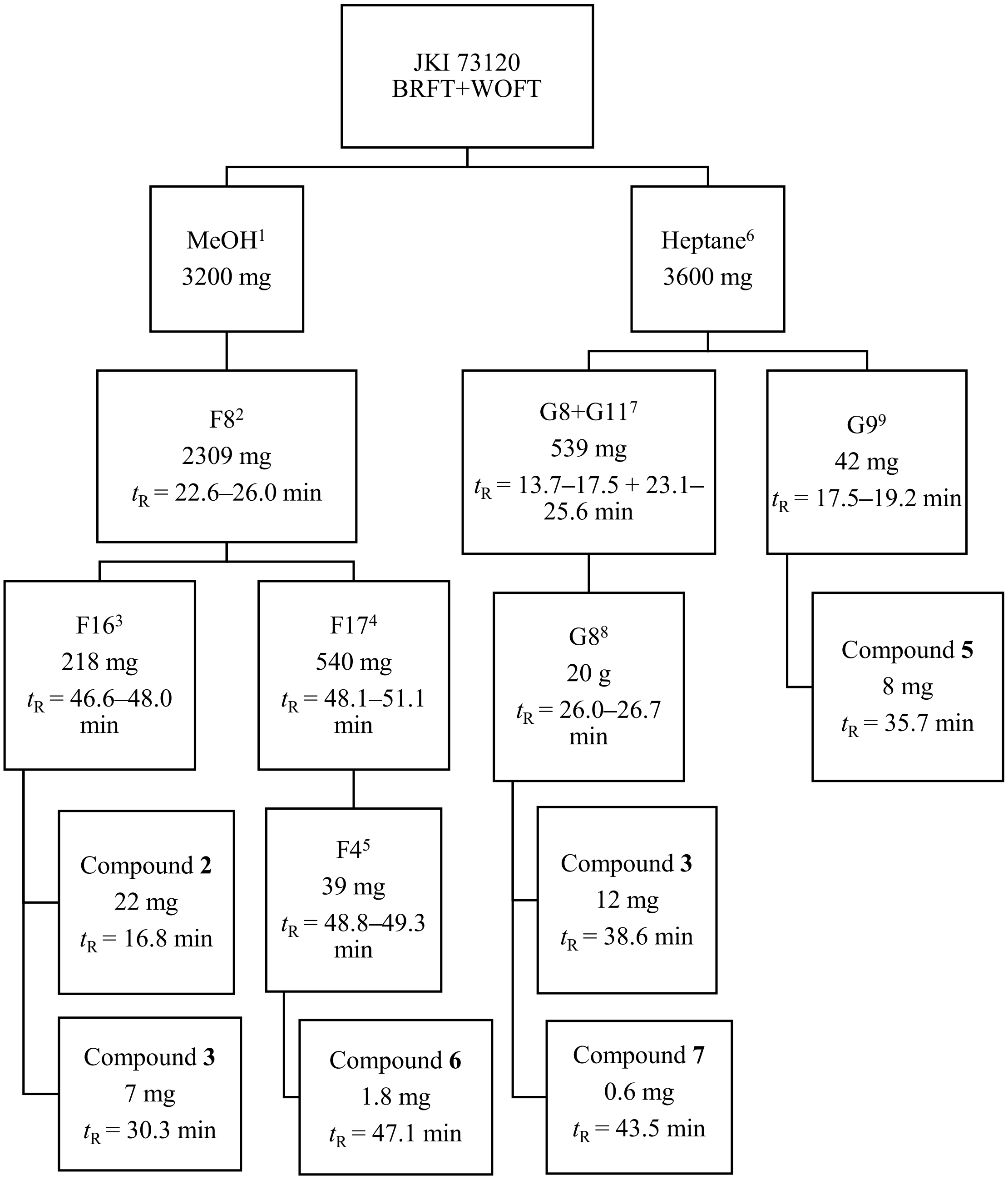

Scheme S1. Separation scheme of the isolated compounds **2**, **3**, and **5**–**7** from *P. karssenii* strain JKI 73120.

Table S3. Chromatographic separation parameters for the BRFT + WOFT methanol extract of *P. karssenii* strain JKI 73120.

Parameter	Settings
**System**	Büchi Grace Reveleris X2 flash chromatography system (Büchi Labortechnik AG, Flawil, Switzerland)
**Column**	80 g silica cartridge (FlashPure ID Silica, Büchi Labortechnik AG, Flawil, Switzerland)
**Solvent A**	*n*-heptane + 0.1% FA
**Solvent B**	58% *n*-heptane, 40% TBME, 2% MeOH + 0.1% FA

**Solvent C**	MeOH + 0.1% FA
**Flow rate [mL min^{-1}]**	60
**Fraction volume [mL]**	20
**Sample amount [mg]**	3200
**Repetitions**	1
**Gradient [t_{min}]**	t_0 = 0% A**B**, t_3 = 0% A**B**, t_{18} = 100% A**B**, t_{21} = 100% A**B**, t_{31} = 50% B**C**, t_{34} = 50% B**C**

Table S4. Chromatographic separation parameters for fraction F8 of *P. karssenii* strain JKI 73120 (BRFT + WOFT methanol extract).

**Parameter**	**Settings**
**System**	Büchi Pure C-850 Flash Prep (Büchi Labortechnik AG, Flawil, Switzerland)
**Column**	Gemini 10 µm C18 110 Å column (250 x 50 mm, Phenomenex, Aschaffenburg, Germany)
**Solvent A**	H_2O + 0,1% FA
**Solvent B**	MeCN + 0,1% FA
**Flow rate [mL min^{-1}]**	50
**Fraction volume [mL]**	7
**Sample amount [mg]**	2300
**Repetitions**	4
**Gradient [t_{min}]**	t_0 = 15% B, t_3 = 15% B, t_6 = 50% B, t_{26} = 60% B, t_{41} = 80% B, t_{51} = 100% B, t_{56} = 100% B

Table S5. Chromatographic separation parameters for fraction F16 (from F08, BRFT + WOFT methanol extract of *P. karssenii* strain JKI 73120).

**Parameter**	**Settings**
**System**	Gilson PLC 2050 (Gilson, Middleton, WI, USA)
**Column**	Nucleodur C18 ec 110 Å column (150 x 40 mm, 10 µm, Macherey-Nagel, Düren, Germany)
**Solvent A**	H_2O + 0.1% FA
**Solvent B**	MeCN + 0.1% FA
**Flow rate [mL min^{-1}]**	40
**Fraction volume [mL]**	14
**Sample amount [mg]**	434
**Repetitions**	2
**Gradient [t_{min}]**	t_0 = 60% B, t_5 = 60% B, t_{45} = 75% B, t_{50} = 100% B, t_{55} = 100% B

Table S6. Chromatographic separation parameters for fraction F17 (from F08, BRFT + WOFT methanol extract of *P. karssenii* strain JKI 73120).

**Parameter**	**Settings**
**System**	Gilson PLC 2050 (Gilson, Middleton, WI, USA)
**Column**	Nucleodur C18 HTec 110 Å column (250 × 40 mm, 10 µm, Macherey-Nagel, Düren, Germany)

**Solvent A**	H_2O + 0.1% FA
**Solvent B**	MeCN + 0.1% FA
**Flow rate [mL min^{-1}]**	40
**Fraction volume [mL]**	14
**Sample amount [mg]**	590
**Repetitions**	2
**Gradient [t_{min}]**	t_0 = 70% B, t_5 = 70% B, t_{45} = 90% B, t_{50} = 100% B, t_{55} = 100% B

Table S7. Chromatographic separation parameters for fraction F4 (from F17 → F08, BRFT + WOFT MeOH extract of *P. karssenii* strain JKI 73120).

Parameter	Settings
**System**	Büchi Pure C-850 Flash Prep (Büchi Labortechnik AG, Flawil, Switzerland)
**Column**	XBridge BEH 5 µm C18 column (250 × 19 mm; Waters, Eschborn, Germany)
**Solvent A**	H_2O + 0.1% FA
**Solvent B**	MeCN + 0.1% FA
**Flow rate [mL min^{-1}]**	20
**Fraction volume [mL]**	6
**Sample amount [mg]**	39
**Repetitions**	2
**Gradient [t_{min}]**	t_0 = 52% B, t_5 = 52% B, t_{55} = 55% B, t_{65} = 100% B, t_{70} = 100% B

Table S8. Chromatographic separation parameters for the BRFT + WOFT *n*-heptane extract of *P. karssenii* strain JKI 73120.

Parameter	Settings
**System**	Büchi Grace Reveleris X2 flash chromatography system (Büchi Labortechnik AG, Flawil, Switzerland)
**Column**	80 g silica cartridge (FlashPure ID Silica, Büchi Labortechnik AG, Flawil, Switzerland)
**Solvent A**	*n*-heptane + 0.1% FA
**Solvent B**	58% *n*-heptane, 40% TBME, 2% MeOH + 0.1% FA
**Solvent C**	37.5% acetone, 37.5% DCM, 25% MeOH + 0.1% FA
**Flow rate [mL min^{-1}]**	60
**Fraction volume [mL]**	20
**Sample amount [mg]**	3600
**Repetitions**	1
**Gradient [t_{min}]**	t_0 = 0% A**B**, t_3= 0% A**B**, t_{18} = 100% A**B**, t_{21} = 100% A**B**, t_{31} = 100% B**C**, t_{34} = 100% B**C**

Table S9. Chromatographic separation parameters for fraction G8+G11 of *P. karssenii* strain JKI 73120 (BRFT + WOFT *n*-heptane extract).

**Parameter**	**Settings**
**System**	Büchi Pure C-850 Flash Prep (Büchi Labortechnik AG, Flawil, Switzerland)
**Column**	Luna 5 µm C18 110 Å column (250 × 21.2 mm; Phenomenex, Aschaffenburg, Germany)
**Solvent A**	H_2O + 0.1% FA
**Solvent B**	MeCN + 0.1% FA
**Flow rate [mL min^{-1}]**	20
**Fraction volume [mL]**	10 peak, 20 non-peak
**Sample amount [mg]**	540 mg
**Repetitions**	6
**Gradient [t_{min}]**	t_0 = 60% B, t_5 = 60% B, t_{25} = 80% B, t_{30} = 100% B, t_{45} = 100% B

Table S10. Chromatographic separation parameters for fraction G8 (from G8+G11, BRFT + WOFT *n*-heptane extract of *P. karssenii* strain JKI 73120).

**Parameter**	**Settings**
**System**	Gilson PLC 2050 (Gilson, Middleton, WI, USA)
**Column**	XBridge BEH 5 µm C18 column (250 × 19 mm; Waters, Eschborn, Germany)
**Solvent A**	H_2O + 0.1% FA
**Solvent B**	MeCN + 0.1% FA
**Flow rate [mL min^{-1}]**	20
**Fraction volume [mL]**	7
**Sample amount [mg]**	20
**Repetitions**	1
**Gradient [t_{min}]**	t_0 = 50% B, t_5 = 50% B, t_{10} = 55% B, t_{40} = 55% B, t_{45} = 100% B, t_{50} = 100% B

Table S11. Chromatographic separation parameters for fraction G9 of *P. karssenii* strain JKI 73120 (BRFT + WOFT *n*-heptane extract).

**Parameter**	**Settings**
**System**	Gilson PLC 2050 (Gilson, Middleton, WI, USA)
**Column**	Luna 5 µm C18 110 Å column (250 × 21.2 mm; Phenomenex, Aschaffenburg, Germany)
**Solvent A**	H_2O + 0.1% FA
**Solvent B**	MeCN + 0.1% FA
**Flow rate [mL min^{-1}]**	20
**Sample amount [mg]**	42
**Repetitions**	1
**Gradient [t_{min}]**	t_0 = 65% B, t_{30} = 65% B, t_{35} = 100% B, t_{45} = 100% B

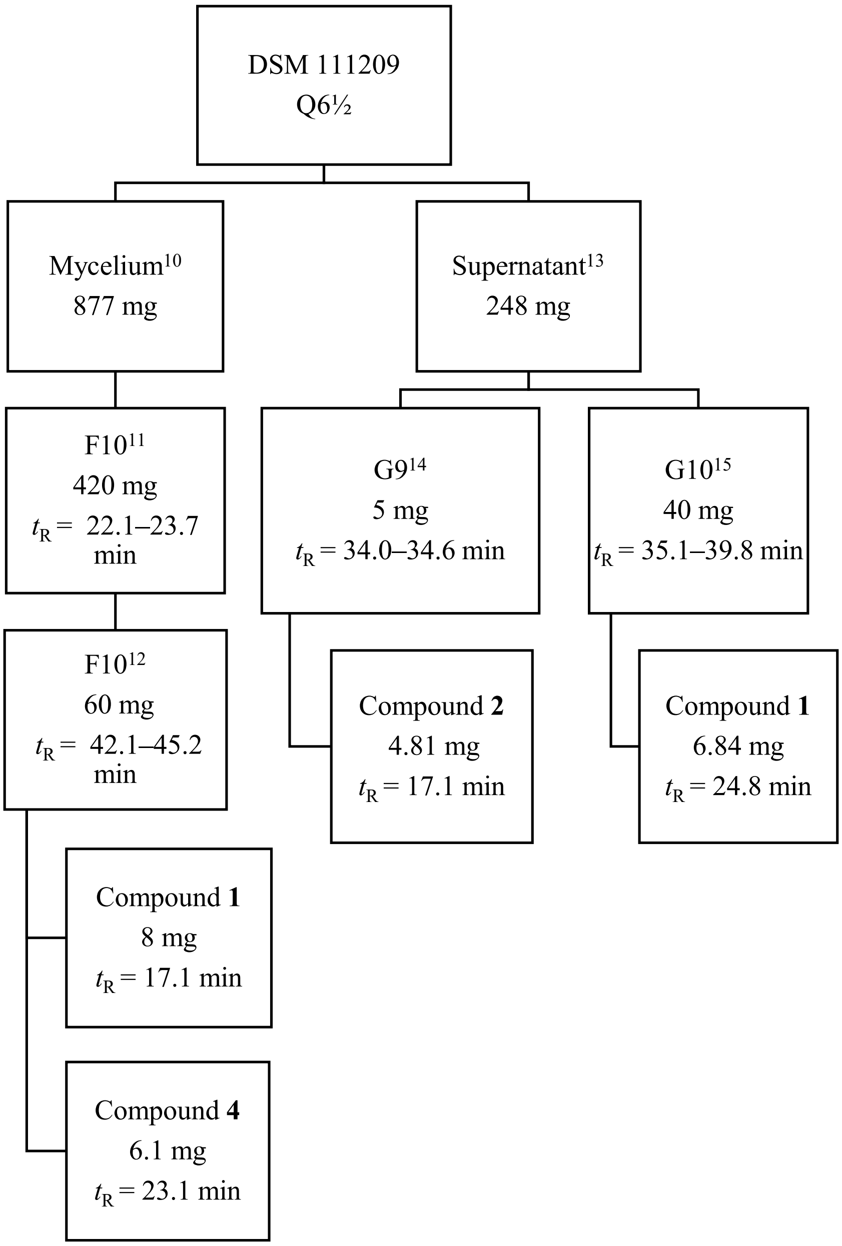

Scheme S2. Separation scheme of the isolated compounds **1**, **2**, and **4** from *P. karssenii* strain DSM 111209.

Table S12. Chromatographic separation parameters for the mycelial extract of *P. karssenii* strain DSM 111209.

Parameter	Settings
**System**	Büchi Pure C-850 Flash Prep (Büchi Labortechnik AG, Flawil, Switzerland)
**Column**	12 g silica cartridge (FlashPure ID Silica, Büchi Labortechnik AG, Flawil, Switzerland)
**Solvent A**	*n*-heptane + 0.1% FA
**Solvent B**	63% *n*-heptane, 35.5% TBME, 1.5% MeOH + 0.1% FA
**Solvent C**	MeOH + 0.1% FA
**Flow rate [mL min^{-1}]**	60

**Fraction volume [mL]**	10 peak, 20 non-peak
**Sample amount [mg]**	877
**Repetitions**	1
**Gradient [t_{min}]**	t_0 = 0% A**B**, t_3 = 0% A**B**, t_{18} = 100% A**B**, t_{21} = 100% A**B**, t_{31} = 50% B**C**, t_{33} = 50% B**C**, t_{34} = 100% B**C**, t_{39} = 100% B**C**

Table S13. Chromatographic separation parameters for fraction F10 of *P. karssenii* strain DSM 111209 (mycelial extract).

Parameter	Settings
**System**	Büchi Pure C-850 Flash Prep (Büchi Labortechnik AG, Flawil, Switzerland)
**Column**	Gemini 10 µm C18 110 Å column (250 x 50 mm, Phenomenex, Aschaffenburg, Germany)
**Solvent A**	H_2O + 0.1% FA
**Solvent B**	MeCN + 0.1% FA
**Flow rate [mL min^{-1}]**	50
**Fraction volume [mL]**	17 peak, 22 non-peak
**Sample amount [mg]**	420
**Repetitions**	2
**Gradient [t_{min}]**	t_0 = 50% B, t_5 = 50% B, t_{45} = 100% B, t_{55} = 100% B

Table S14. Chromatographic separation parameters for fraction F10 (from F10, mycelial extract of *P. karssenii* strain DSM 111209).

Parameter	Settings
**System**	Gilson PLC 2050 (Gilson, Middleton, WI, USA)
**Column**	Luna 5 µm C18 110 Å column (250 × 21.2 mm; Phenomenex, Aschaffenburg, Germany)
**Solvent A**	H_2O + 0.1% FA
**Solvent B**	MeCN + 0.1% FA
**Flow rate [mL min^{-1}]**	20
**Fraction volume [mL]**	7
**Sample amount [mg]**	60
**Repetitions**	2
**Gradient [t_{min}]**	t_0 = 65% B, t_5 = 65% B, t_{45} = 90% B, t_{47} = 100% B, t_{57} = 100% B

Table S15. Chromatographic separation parameters for the supernatant extract of *P. karssenii* strain DSM 111209.

Parameter	Settings
**System**	Büchi Pure C-850 Flash Prep (Büchi Labortechnik AG, Flawil, Switzerland)
**Column**	Gemini 10 µm C18 110 Å column (250 x 50 mm, Phenomenex, Aschaffenburg, Germany)
**Solvent A**	H_2O + 0.1% FA

**Solvent B**	MeCN + 0.1% FA
**Flow rate [mL min^{-1}]**	50
**Fraction volume [mL]**	7
**Sample amount [mg]**	248
**Repetitions**	1
**Gradient [t_{min}]**	t_0 = 50% B, t_5 = 50% B, t_{45} = 100% B, t_{55} = 100% B

Table S16. Chromatographic separation parameters for fraction G9 of *P. karssenii* strain DSM 111209 (supernatant extract).

Parameter	Settings
**System**	1200 Infinity Series (Agilent Technologies Deutschland GmbH, Böblingen, Germany)
**Column**	XBridge BEH 5 µm C18 column (250 × 10 mm; Waters, Eschborn, Germany)
**Solvent A**	H_2O + 0.1% FA
**Solvent B**	MeCN + 0.1% FA
**Flow rate [mL min^{-1}]**	5
**Sample amount [mg]**	5
**Repetitions**	1
**Gradient [t_{min}]**	t_0 = 50% B, t_3 = 50% B, t_{19} = 70% B, t_{21} = 100% B, t_{24} = 100% B

Table S17. Chromatographic separation parameters for fraction G10 of *P. karssenii* strain DSM 111209 (supernatant extract).

Parameter	Settings
**System**	Gilson PLC 2050 (Gilson, Middleton, WI, USA)
**Column**	Luna 5 µm C18 110 Å column (250 × 21.2 mm; Phenomenex, Aschaffenburg, Germany)
**Solvent A**	H_2O + 0.1% FA
**Solvent B**	MeCN + 0.1% FA
**Flow rate [mL min^{-1}]**	20
**Fraction volume [mL]**	7
**Sample amount [mg]**	40
**Repetitions**	1
**Gradient [t_{min}]**	t_0 = 60% B, t_{30} = 60% B, t_{35} = 100% B, t_{45} = 100% B

Table S18. Cytotoxicity (IC_{50}) of compounds **1**–**7**.

Test Cell Line	IC_{50} (µM)							Epothilone B (nM)
	**1**	**2**	**3**	**4**	**5**	**6**	**7**	
L929	n.a	n.a	n.a	n.a	n.a	n.a	n.a	0.65
KB3.1	n.a	n.a	n.a	n.a	**53.6**	n.a	**24.1**	0.17

n.a: No activity

Table S19. Antimicrobial activity (MIC) of compounds **1**–**7**.

Test Microorganism	MIC (µg/mL)							Reference (µg/mL)
	**1**	**2**	**3**	**4**	**5**	**6**	**7**	
S. aureus	n.i	n.i	**33.3**	n.i	n.i	n.i	**66.6**	0.21[G]
M. smegmatis	n.i	n.i	n.i	n.i	n.i	n.i	**66.6**	1.70[K]
B. subtilis	**66.6**	n.i	**16.6**	n.i	**66.6**	n.i	**66.6**	16.6[O]
A. baumannii	n.i	n.i	n.i	n.i	n.i	n.i	n.i	0.52[C]
P. aeruginosa	n.i	n.i	n.i	n.i	n.i	n.i	n.i	0.21[G]
E. coli	n.i	n.i	n.i	n.i	n.i	n.i	n.i	0.42[G]
C. violaceum	n.i	n.d	n.i	n.i	n.i	n.i	n.d	1.70[G]
C. albicans	n.i	n.i	n.i	n.i	**66.6**	n.i	n.i	8.3[N]
S. pombe	n.i	n.i	n.i	n.i	n.i	n.i	n.i	8.30[N]
M. hiemails	n.i	n.i	**66.6**	n.i	**16.6**	n.i	n.i	8.30[N]
R. toruloides	n.i	n.i	n.i	n.i	**66.6**	n.i	n.d	4.20[N]
W. anomalus	n.i	n.i	n.i	n.i	n.i	n.i	n.i	16.6[N]

n.i: No inhibition (>66.6 µg/mL). n.d: Not determined.
G: Gentamycin; O: Oxytetracycline; N: Nystatin; C: Ciprofloxacin; K: Kanamycin.

Table S20. Nematicidal activity of compounds **1**–**7**.

Compound	Corrected mortality rate [%]			Positive control
	100 µg/mL	50 µg/mL	10 µg/mL	
1	57.6 ± 10.8	29.0 ± 6.6	9.0 ± 4.6	93.4 ± 4.1
2	83.1 ± 5.2	28.5 ± 5.1	0	
3	0	0	0	
4	0	0	0	
5	12.8 ± 3.9	4.0 ± 1.3	0	
6	0	0	0	
7	13.8 ± 7.2	0	0	

Positive control: Ivermectin (1 µg/mL).
The mortality rate in the negative control (MeOH) was 14.7±3.5%, and was used to calculate the corrected mortality using the Schneider-Orelli's formula.
